# 4‑Aminoalkylquinolines as Potent Antitubercular Agents Targeting the Cytochrome bc_1_ Complex

**DOI:** 10.1021/acsmedchemlett.6c00183

**Published:** 2026-05-15

**Authors:** Estevão Silveira Grams, Alessandro Silva Ramos, Fernanda Fries da Silva, Marcia Alberton Perelló, Alexia de Matos Czeczot, Ariel Moura Maia, Higor Arruda Caetano, Stefani Altenhofen, Josiane Delgado Paz, Guilherme Arraché Gonçalves, Xinyi Grace Liu, Teresa Repasy, Luisa Dreher Klein, Gabriela Pagnoncelli Polesello, Carlos Alexandre Sanchez Ferreira, Sílvia Dias de Oliveira, Rafael Stieler, Sidnei Moura e Silva, Carla Denise Bonan, Cristiano Valim Bizarro, Luiz Augusto Basso, Tanya Parish, Pablo Machado

**Affiliations:** † Instituto Nacional de Ciência e Tecnologia em Tuberculose, Centro de Pesquisas em Biologia Molecular e Funcional, 28102 Pontifícia Universidade Católica do Rio Grande do Sul, 90616-900, Porto Alegre, Rio Grande do Sul, Brazil; ‡ Programa de Pós-Graduação em Biologia Celular e Molecular, Pontifícia Universidade Católica do Rio Grande do Sul, 90616-900, Porto Alegre, Rio Grande do Sul, Brazil; § Programa de Pós-Graduação em Medicina e Ciências da Saúde, Pontifícia Universidade Católica do Rio Grande do Sul, 90616-900, Porto Alegre, Rio Grande do Sul, Brazil; ∥ Laboratório de Neuroquímica e Psicofarmacologia, Pontifícia Universidade Católica do Rio Grande do Sul, 90619-900, Porto Alegre, RS, Rio Grande do Su, Brazil; ⊥ Laboratório de Imunologia e Microbiologia, Pontifícia Universidade Católica do Rio Grande do Sul, 90619-900, Porto Alegre, Rio Grande do Sul, Brazil; # Laboratório de Catálise Molecular, Instituto de Química, Universidade Federal do Rio Grande do Sul, 90501-970, Porto Alegre, Rio Grande do Sul, Brazil; g Laboratório de Biotecnologia de Produtos Naturais e Sintéticos, Instituto de Biotecnologia, Universidade de Caxias do Sul, 95070-560, Caxias do Sul, Rio Grande do Sul, Brazil; h Center for Global Infectious Disease Research, 549448 Seattle Children’s Research Institute, Seattle, Washington 98109, United States of America; i Department of Pediatrics, University of Washington School of Medicine, Seattle, Washington 98109, United States of America

**Keywords:** 4-aminoalkylquinolines, antitubercular agents, cytochrome bc_1_

## Abstract

The development of new chemotypes active against drug-resistant *Mycobacterium tuberculosis* remains a major priority in tuberculosis drug discovery. Herein, a series of 4-aminoalkylquinolines was designed and synthesized to improve antimycobacterial potency and permeability. Structure–activity relationship studies identified key contributions of alkyl substitution at C-2 and hydrophobic terminal phenyl substituents. The most active derivatives exhibited submicromolar to low-nanomolar activity (MIC = 0.02–0.05 μM) and retained activity against multidrug-resistant clinical isolates. Evaluation against a QcrB_T313I_ strain indicated reduced susceptibility, consistent with inhibition of the cytochrome bc_1_ complex. Single-crystal X-ray diffraction confirmed the structure of a representative compound. Selected molecules showed favorable selectivity in Vero and HepG2 cells and limited activity against non-mycobacterial bacteria. In vitro ADME profiling revealed pH-dependent solubility, good permeability, and rapid metabolic turnover. Zebrafish assays showed no detectable cardiac effects up to 0.3 μM. Overall, this chemotype represents a promising scaffold for antitubercular agents targeting mycobacterial respiration.

## Graphical abstract

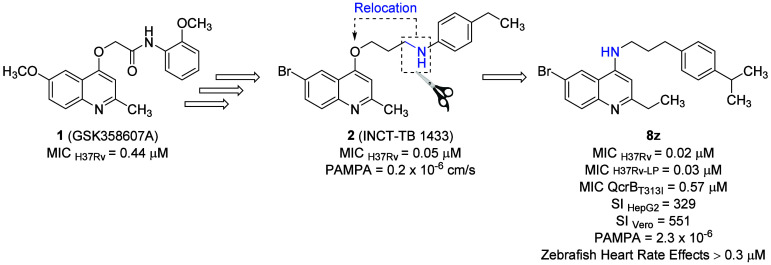

Tuberculosis (TB), caused mainly by *Mycobacterium tuberculosis*, remains a major global health threat. According to WHO, approximately 10.7 million people developed TB in 2024, corresponding to an incidence of 131 cases per 100,000 population/year.^1^ Despite advances in diagnosis and care, TB caused ∼1.23 million deaths in 2024, reaffirming its status as the deadliest infection caused by a single pathogen.^1^ Drug-resistant TB continues to challenge control efforts: of an estimated ∼390,000 cases of rifampicin-resistant or multidrug-resistant TB (RR/MDR-TB) in 2024, only ∼164,500 (≈42%) accessed appropriate treatment.^1^ Current TB chemotherapy relies on multidrug regimens that require prolonged administration and are increasingly challenged by drug-resistant strains. The approval of bedaquiline, delamanid, and pretomanid over the past decade represents the most significant advance in TB therapeutics since the 1960s.^2–4^ These agents, used in all-oral, shortened regimens such as BPaL and BPaLM, have achieved ∼90% treatment success rates in highly drug-resistant cases.^4,5^ However, their clinical impact is constrained by safety concerns (notably QT prolongation and linezolid-related toxicity), limited access in high-burden settings, and the risk of emerging resistance.^2,6^ As a result, there is a continuing need for new chemotypes that can be incorporated into safe, short, and fully oral regimens with robust activity against drug-susceptible and drug-resistant *M. tuberculosis*. In this scenario, oxidative phosphorylation is essential for *M. tuberculosis* survival in both replicating and persistent states, rendering the electron transport chain an attractive pharmacological target.^7^ The cytochrome bc_1_–aa_3_ supercomplex plays a central role in respiratory ATP generation, and its inhibition disrupts the proton motive force and cellular bioenergetics, leading to rapid bactericidal effects.^8^ Inhibition of cytochrome bc_1_ further disrupts respiration, resulting in rapid bactericidal activity and enhanced sterilizing potential.^8,9^ This vulnerability has been clinically validated by the development of cytochrome bc_1_ inhibitors such as Q203 (telacebec), which has demonstrated potent activity against drug-susceptible and drug-resistant *M. tuberculosis* strains.^8^ In line with the continuing need for alternative drugs, 2-(quinolin-4-yloxy)­acetamides were identified by high-throughput screening as a promising antitubercular chemotype^10^ and subsequently optimized by our group. Early studies established potent activity against drug-susceptible and drug-resistant *M. tuberculosis*, followed by structure–activity relationship optimization and synergistic drug-combination profiling.^11,12^ Subsequent refinements improved drug-like properties while preserving antimycobacterial potency.^13^ More recently, scaffold-hopping efforts further expanded this chemical space, while the identification of 4-alkoxyquinolines provided additional evidence of selective antimycobacterial activity and mechanistic relevance.^14,15^ Together, these studies reinforce quinoline-based compounds as a versatile platform for TB drug discovery.

In this context, a new class of 4-aminoalkylquinolines was designed, synthesized, and evaluated against the drug-susceptible *M. tuberculosis* H37Rv strain. This scaffold evolved from 4-(quinolin-4-yloxy)­acetamides, which, after three optimization campaigns, led to the 4-alkoxyquinoline series previously reported by our group (Figure 1).^15^ Relocation of the aniline nitrogen from the side chain to the C-4 position of the quinoline ring generated the present chemotype to explore whether this modification could favor passive permeability while maintaining antimycobacterial activity (Figure 1). The most promising compounds were further assessed against a panel of well-characterized multidrug-resistant strains. Their mechanism of action was investigated using spontaneously resistant mutants followed by whole-genome sequencing. Additionally, mammalian cell viability assays in Vero and HepG2 cells provided an initial evaluation of the selectivity and cytotoxicity. In parallel, in vitro ADME profiling encompassed the chemical stability, kinetic solubility, passive permeability, and metabolic stability. Finally, in vivo toxicity was evaluated by using a zebrafish model.

**Figure 1:**
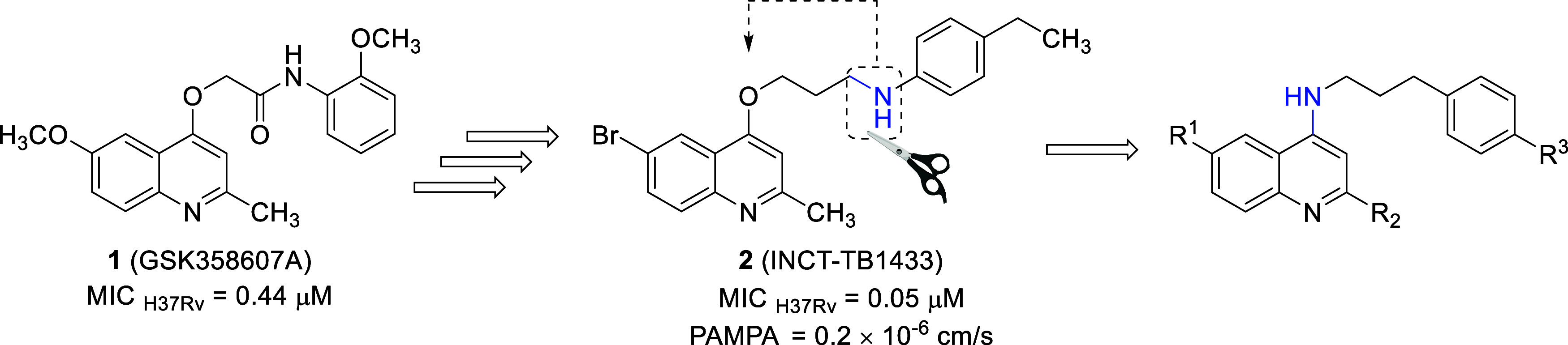
Design strategy leading to the 4-aminoalkylquinoline series. High-throughput screening identified 4-(quinolin-4-yloxy)­acetamides as initial hits, which after three optimization campaigns led to the 4-alkoxyquinoline scaffold previously reported by our group. In the present study, relocation of the aniline nitrogen from the side chain to the C-4 position of the quinoline ring generated the new 4-aminoalkylquinoline chemotype.

The target compounds were synthesized in three steps (Scheme 1). Initially, 2-alkyl-4-hydroxyquinolines (**5a**–**f**) were prepared via a Conrad–Limpach sequence from anilines (**3a**–**d**) and β-ketoesters (**4a**–**b**), following the procedure already described.^12^ In this step, condensation in ethanol with MgSO_4_ and acetic acid afforded β-aminoacrylate intermediates, which were directly cyclized in Dowtherm A at 230–250 °C for 15 min to furnish the quinoline core in 20–38% yield. Subsequently, chlorination with POCl_3_ in refluxing toluene converted compounds **5a**–**h** into the corresponding 2-alkyl-4-chloroquinolines (**6a**–**f**) in 85–93% yield; notably, substituent variation at the C-2 and C-6 positions had minimal impact on reaction efficiency. Finally, S_N_Ar coupling of **6a**–**f** with 3-phenylpropan-1-amines (**7a**–**f**) in DMSO using DIPEA under reflux afforded the target 4-aminoalkylquinolines (**8a**–**z**) in 22–58% yield, with variations in isolated yields primarily associated with purification efficiency. In addition to spectroscopy and spectrometric data, the structure of molecule **8z** was confirmed by single-crystal X-ray diffraction (Figure 2).

**Scheme 1:**
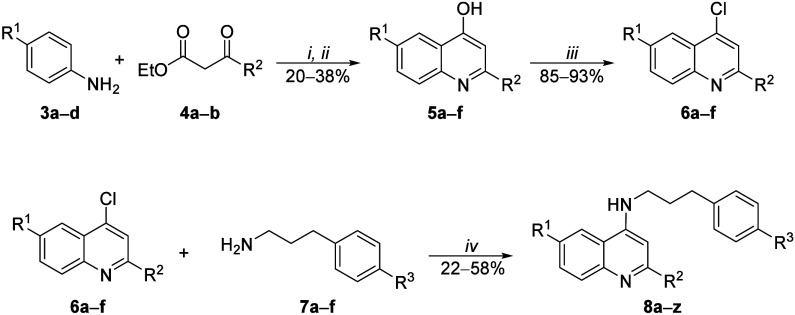
Target Compound Synthesis^a^

a Reactants and conditions: *i* = MgSO_4_, AcOH, EtOH, 90 °C, 16 h; *ii* = Dowtherm® A, 230–250 °C, 15 min; *iii* = POCl_3_, Toluene, 110 °C, 2 h; *iv* = DIPEA, DMSO, 150 °C, 20 h.

**Figure 2:**
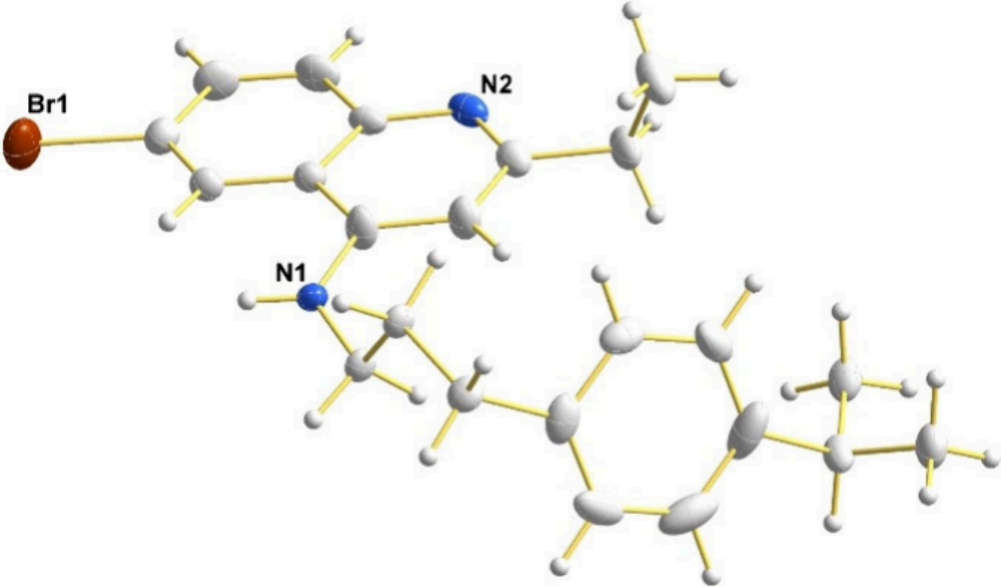
Molecular structure of **8z** with thermal ellipsoids drawn at the 50% probability level. The crystal structure contains disorder in the propyl and isopropyl groups; for clarity, only one disorder component was shown (CCDC number 2540064).

All synthesized 4-aminoalkylquinolines were evaluated for in vitro activity against the drug-susceptible *M. tuberculosis* H37Rv strain using the resazurin reduction microplate assay (REMA) (Table 1).^16^ The series displayed a broad activity range, with MIC values spanning from 0.02 to 14.86 μM. Several derivatives exhibited submicromolar potency, and the most active compounds reached nanomolar-range activity, surpassing isoniazid under the same conditions (2.26 μM). These findings indicate that the quinoline scaffold accommodates extensive structural diversification while retaining antimycobacterial activity.

**Table 1:**
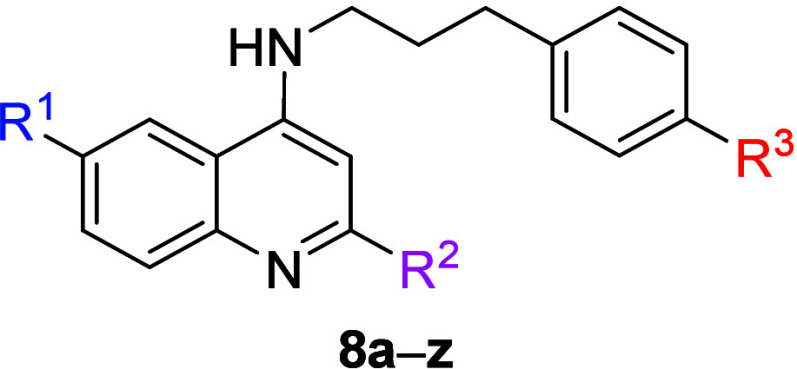
Yields of 4-Aminoalkylquinolines **8a**–**z**, ClogP Values, and In Vitro Activities Against *M. tuberculosis* H37Rv Strain

entry	R^1^	R^2^	R^3^	yields^a^ (%)	ClogP^b^	MIC H37Rv (μM)^c^
**8a**	Cl	Me	H	47	6.29	0.51
**8b**	Br	Me	H	53	6.44	0.45
**8c**	I	Me	H	29	6.70	0.77
**8d**	MeO	Me	H	58	5.79	8.16
**8e**	Cl	Me	MeO	35	6.20	0.47
**8f**	Br	Me	MeO	33	6.35	0.42
**8g**	I	Me	MeO	23	6.61	2.89
**8h**	MeO	Me	MeO	32	5.71	14.86
**8i**	Cl	Me	Me	36	6.78	0.49
**8j**	Br	Me	Me	33	6.93	0.84
**8k**	I	Me	Me	23	7.19	1.51
**8l**	MeO	Me	Me	37	6.29	3.90
**8m**	Cl	Me	*i*Pr	44	7.71	0.22
**8n**	Br	Me	*i*Pr	36	7.86	0.20
**8o**	I	Me	*i*Pr	25	8.12	0.70
**8p**	Cl	Me	*t*Bu	26	8.11	0.84
**8q**	Br	Me	*t*Bu	27	8.26	0.75
**8r**	I	Me	*t*Bu	22	8.52	0.68
**8s**	MeO	Me	*t*Bu	33	7.62	3.45
**8t**	Cl	Me	Cl	41	6.99	0.90
**8u**	Br	Me	Cl	33	7.15	0.80
**8v**	MeO	Me	Cl	31	6.50	7.33
**8x**	Cl	Et	H	29	6.81	1.94
**8y**	Br	Et	H	31	6.96	0.84
**8w**	Cl	Et	*i*Pr	24	8.24	0.11
**8z**	Br	Et	*i*Pr	25	8.39	0.02
**INH**		-		-	-	2.26

a Obtained after column purification.

b ClogP was calculated by ChemBioDraw Ultra, version 12.0.2.

c Minimal inhibitory concentration (MIC) was obtained from the resazurin reduction microplate assay (REMA). INH, isoniazid.

Structure–activity relationship analysis revealed distinct contributions from substitutions at C-6 (R^1^), C-2 (R^2^), and the terminal phenyl ring (R^3^). At C-6, halogen substitution consistently favored potency. Chloro- and bromo-derivatives generally outperformed iodo analogues, whereas methoxy substitution led to a pronounced reduction in activity. This pattern was maintained across multiple R^3^ substitution profiles, supporting a favorable electronic or steric contribution of halogens at this position. Substitutions at C-2 exerted a secondary but measurable influence. Replacement of methyl with ethyl tended to reduce activity when the terminal phenyl ring was unsubstituted; however, this effect diminished or reversed in bulkier R^3^ environments, suggesting a steric interplay between these regions. By contrast, modifications at the terminal phenyl ring (R^3^) produced the most pronounced activity shifts. Increasing hydrophobic bulk improved potency, with isopropyl-substituted derivatives emerging as the most active subgroup. Combinations of halogen substitution at C-6 and bulky hydrophobic groups at R^3^ consistently yielded submicromolar to nanomolar activity, whereas polar substituents (e.g., methoxy) markedly reduced potency. Finally, calculated lipophilicity showed a moderate association with activity. Linear regression between ClogP and *p*MIC values yielded an R^2^ of 0.35, indicating that increased hydrophobicity may contribute to enhanced antimycobacterial potency, possibly by favoring cell envelope permeation or target engagement; however, lipophilicity alone does not fully explain activity differences within the series.

Based on the potency profile observed for the H37Rv strain, 4-aminoalkylquinolines meeting a MIC threshold of ≤0.50 μM were prioritized for evaluation against multidrug-resistant clinical isolates (Table 2). Eight compounds were selected for further investigation (**8b**, **8e**, **8f**, **8i**, **8m**, **8n**, **8w**, and **8z**). It is noteworthy that molecule **8z** displayed improved antimycobacterial potency relative to structure **2**, with MIC values of 0.02 and 0.05 μM against *M. tuberculosis* H37Rv, respectively. The selected compounds were tested against three well-characterized MDR-*M. tuberculosis* strains (PT2, PT12, and PT20) whose genetic determinants underlying resistance phenotypes are well established.^17^ The isolates exhibit resistance to first-line agents including isoniazid, rifampicin, streptomycin, ethionamide, and rifabutin; notably, PT12 and PT20 are additionally resistant to pyrazinamide and ethambutol, and PT12 also displays resistance to amikacin and capreomycin.^17^ All selected molecules retained inhibitory activity against the resistant isolates, with MIC values generally comparable to or lower than those observed for the drug-susceptible H37Rv strain. In several cases, enhanced potency was observed against MDR strains, particularly for derivatives **8m**, **8n**, **8w**, and **8z**, which displayed submicromolar to nanomolar activity across all tested isolates. In contrast, reference drugs showed the expected resistance profile, with markedly reduced activity for isoniazid and rifampicin. Collectively, these findings demonstrate that the synthesized 4-aminoalkylquinolines series maintains robust activity against clinically relevant MDR-TB strains.

**Table 2:** In Vitro Antimycobacterial Activity against Drug-Susceptible and Multidrug-Resistant *M. tuberculosis* Strains and Viability of the Vero and HepG2 Cells

entry	MIC H37Rv (μM)^a^	MIC PT2 (μM)^a^	MIC PT12 (μM)^a^	MIC PT20 (μM)^a^	CC^50^ Vero^b^(μM)	CC^50^ HepG2^b^(μM)	SI Vero^e^	SI HepG2^e^
**8b**	0.45	0.11	0.11	0.23	17.5^c^8.9^d^	15.9^c^ 10.6^d^	38.8^c^ 19.8^d^	35.4^c^ 23.7^d^
**8e**	0.47	0.23	0.47	0.23	16.4^c^15.5^d^	17.1^c^ 15.9^d^	34.9^c^ 33.0^d^	36.4^c^ 33.9^d^
**8f**	0.42	0.21	0.42	0.21	16.7^c^13.0^d^	17.1^c^ 15.9^d^	39.6^c^ 31.0^d^	40.6^c^ 37.2^d^
**8i**	0.49	<0.12	0.25	0.12	19.3^c^11.4^d^	15.9^c^11.3^d^	39.5^c^23.3^d^	32.4^c^ 23.1^d^
**8m**	0.22	<0.11	<0.11	<0.11	10.4^c^3.6^d^	6.7^c^ 6.0^d^	52.0^c^15.9^d^	29.1^c^ 26.0^d^
**8n**	0.20	<0.10	<0.10	<0.10	16.0^c^9.5^d^	17.3^c^ 14.7^d^	80.0^c^42.9^d^	78.5^c^ 66.7^d^
**8w**	0.11	<0.11	0.11	<0.11	11.7^c^ 3.3^d^	6.8^c^ 6.2^d^	106^c^ 30^d^	61.8^c^ 56.4^d^
**8z**	0.02	<0.10	<0.10	<0.10	11.0^c^ 3.3^d^	6.6^c^ 5.7^d^	551^c^ 165^d^	329^c^ 283^d^
**INH**	2.3	291.67	145.83	291.67	-	-	-	-
**RIF**	0.01	>48.6	>48.6	>48.6	-	-	-	-

a Minimal inhibitory concentration.

b The toxicity and selectivity of the compounds were investigated using Vero and HepG2 cell lines. The outcomes were quantified as the concentration causing a 50% reduction in cell viability (CC_50_) using MTT and neutral red assays.

c Determined by the MTT method.

d Determined by the Neutral Red method.

e Selectivity index (SI = CC_50_/MIC H37Rv). INH, isoniazid. RIF, rifampin.

To assess the safety profile of prioritized compounds, cytotoxicity was evaluated in Vero and HepG2 cell lines using complementary viability assays (Table 2). Overall, the series showed favorable tolerability, with all derivatives exhibiting selectivity index (SI) values above the commonly accepted threshold of 10 for hit qualification. SI values derived from the MTT assay were consistently higher than those obtained using the Neutral Red uptake (NRU) method, reflecting their distinct biological end points: MTT primarily measures mitochondrial metabolic activity, whereas NRU probes lysosomal integrity and is more sensitive to early structural cell damage. Consequently, structurally compromised cells may retain sufficient mitochondrial function to be scored as viable in MTT but not in NRU, leading to lower CC_50_ values in the latter. Consistent structure–toxicity relationships emerged across the series. Compounds bearing bulky hydrophobic substituents at the terminal phenyl ring, particularly isopropyl-containing derivatives, tended to show reduced CC_50_ values, suggesting that increased lipophilic surface area may favor nonspecific membrane interactions. In contrast, balanced substitution patterns combining halogens at C-6 with moderately hydrophobic terminal groups were associated with improved tolerability. Notably, derivative **8n** maintained high CC_50_ values across both cell lines while preserving strong antimycobacterial potency, resulting in consistently elevated SI values. Among all evaluated compounds, **8w** and **8z** exhibited the most favorable overall profile, combining submicromolar and nanomolar antimycobacterial activity, respectively, with excellent selectivity indices in both assays and cell lines. Collectively, these findings indicate that careful modulation of peripheral hydrophobicity enables preservation of antimicrobial potency while maintaining an acceptable safety margin in mammalian cells.

To further refine the structure–activity relationships, an additional subset of analogues was designed and synthesized by introducing targeted modifications at R^2^ and R^3^ while retaining the most favorable C-6 substituents, namely chlorine and bromine, at R^1^. In parallel, the corresponding 4-alkoxyquinolines were synthesized to examine the effect of replacing the 4-amino functionality with oxygen at the quinoline core and to assess the impact of side-chain extension through incorporation of an aniline-containing moiety. In these analogues, the isopropyl group was maintained at R^3^, given its consistent association with improved potency in the 4-aminoalkylquinoline series. This focused design enabled direct comparison of key structural features influencing antimycobacterial activity. Commercially available 6-bromo-4-hydroxyquinoline **9a** was used as the starting material. 4-Aminoalkylquinolines **10a**–**c** were prepared following the synthetic sequence described in Scheme 1 and were obtained in yields of 21–29% (Scheme 2). Subsequently, the corresponding 4-alkoxyquinolines **11a**–**d** were obtained according to a literature-reported method,^15^ affording the desired products in 14–34% yields (Scheme 2).

**Scheme 2:**
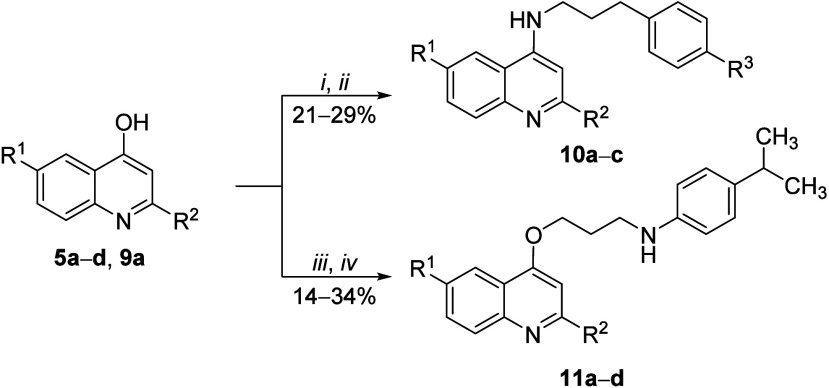
Preparation of 4-Aminoalkylquinolines^a^

a Conditions: *i* = POCl_3_, Toluene, 110 °C, 2 h; *ii* = Amines, DIPEA, DMSO, 150 °C, 20 h. *iii* = 1,3-Dibromopropane, Cs_2_CO_3_, NaI, CH_3_CN, 25 °C, 24 h; *iv* = 4-Isopropylaniline, K_2_CO_3_, NaI, toluene, 110 °C, 48 h.

To obtain quantitative data that more accurately capture the impact of subtle structural modifications on antimycobacterial activity, selected compounds were further evaluated using a sensitive absorbance/fluorescence-based assay.^18^ For this purpose, the *M. tuberculosis* H37Rv-LP strain (ATCC 25618), which constitutively expresses codon-optimized DsRed, was employed.^19^ Overall, the series displayed strong activity, with MIC values against H37Rv-LP ranging from 0.026 to 0.35 μM (Table 3). The most potent compounds were **8m**, **8n**, and **8w**, each exhibiting MIC values of 0.026 to 0.035 μM, while **8z** retained similarly high potency with an MIC of 0.056 μM. Comparison with compound **10a**, which lacks substitution at the C-2 position of the quinoline ring, highlights the importance of alkyl substitution at this position for optimal antimycobacterial activity. 4-Aminoalkylquinolines **8m** and **8n** bear a methyl substituent at C-2, whereas derivatives **8w** and **8z** contain an ethyl group at the same position. The superior potency of these derivatives relative to **10a** (MIC = 0.11 μM) suggests that the presence of a small alkyl substituent at C-2 contributes positively to antimycobacterial activity of this chemical class. Furthermore, insight into the role of the terminal phenyl substituent was obtained by examining derivatives **10b** and **10c**, which incorporate a dimethylamino group on the phenyl ring of the side chain. Introduction of this more polar substituent resulted in a measurable reduction in antimycobacterial potency. In particular, structures **10b** (MIC = 0.18 μM) and **10c** (MIC = 0.35 μM) exhibited an approximately 6-fold reduction in activity relative to their direct analogues **8w** (MIC = 0.03 μM) and **8z** (MIC = 0.056 μM), respectively. Additionally, comparison between the two structural series revealed clear SAR trends. The 4-aminoalkylquinolines **8m**, **8n**, **8w**, and **8z** consistently exhibited higher potency than their corresponding 4-alkoxyquinoline analogues **11a**–**d**, highlighting the importance of the aminoalkyl substituent at the C-4 position and the absence of aniline-derived side chain with extended linker length for potent antimycobacterial activity. While the alkoxy derivatives retained measurable activity, their MIC values were typically 3- to 10-fold higher than those observed for the corresponding 4-amino analogues. Finally, the most potent derivatives (**8m**, **8n**, and **8w**) displayed MIC values of 0.026–0.035 μM against H37Rv-LP, remaining within approximately 9–12-fold of Q203 (MIC = 0.003 μM).

**Table 3:**
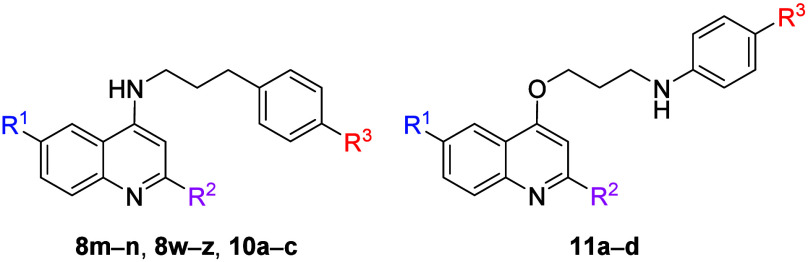
Yields and In Vitro Activity of Selected 4-Aminoalkylquinolines (**8m**–**n**, **8w**–**z**, **10a**–**c**) and 4-Alkoxyquinolines (**11a**–**d**) against *M. tuberculosis* H37Rv-LP (ATCC 25618) and QcrB_T313I_ Strains

entry	R^1^	R^2^	R^3^	yields (%)^a^	MIC H37Rv-LP (μM)^b^	MIC QcrB_T313I_ (μM)^c^
**8m**	Cl	Me	*i*Pr	44	0.026	0.56
**8n**	Br	Me	*i*Pr	36	0.035	0.73
**8w**	Cl	Et	*i*Pr	24	0.030	0.57
**8z**	Br	Et	*i*Pr	25	0.056	0.55
**10a**	Br	H	*i*Pr	21	0.11	0.88
**10b**	Cl	Et	N(Me)_2_	29	0.18	0.83
**10c**	Br	Et	N(Me)_2_	24	0.35	0.86
**11a**	Cl	Me	*i*Pr	34	0.14	0.74
**11b**	Br	Me	*i*Pr	22	0.11	0.54
**11c**	Cl	Et	*i*Pr	34	0.15	0.66
**11d**	Br	Et	*i*Pr	14	0.20	0.84
**Q203**	-	-	-	-	0.003	0.088

a Obtained after column purification.

b Minimal inhibitory concentration (MIC) against H37Rv-LP (ATCC 25618).

c Minimal inhibitory concentration against the QcrB_T313I_ mutant strain. Q203, telacebec. Data are averages of two independent runs.

Afterward, to investigate the mechanism of action, compound activity was evaluated against the QcrB_T313I_ mutant strain (Table 3). This amino acid substitution in the QcrB subunit has been reported to reduce susceptibility to several bc_1_ inhibitors at the Q_P_ site, including the clinical candidate Q203.^8,20^ Across the series, a consistent shift in potency was observed, with MIC values increasing from 0.026–0.35 μM against the parental H37Rv-LP strain to 0.54–0.88 μM in the QcrB_T313I_ mutant, corresponding to an approximately 2.5–20-fold reduction in activity. A similar effect was observed for Q203, whose MIC increased from 0.003 to 0.088 μM against the mutant strain, supporting a comparable sensitivity of the series to this resistance-conferring substitution. Together, these results support the cytochrome bc_1_ complex, specifically the QcrB subunit, as the likely molecular target of novel 4-aminoalkylquinoline-based compounds.

Furthermore, to assess the antibacterial selectivity of this chemotype, two representative 4-aminoalkylquinolines, **8w** and **8z**, were selected for evaluation against a panel of bacterial strains (Table 4). These derivatives were chosen because they combine potent activity against *M. tuberculosis* H37Rv-LP with excellent selectivity indices and represent the C-2 ethyl-substituted analogues within the most active isopropyl-containing subset. A broad-spectrum antibacterial assay was performed against *Staphylococcus aureus* ATCC 25923, *Escherichia coli* ATCC 25922, and additional Gram-positive and Gram-negative strains (Table 4). Overall, both molecules exhibited limited antibacterial activity, with MIC values exceeding 20 μM for most tested organisms. Notably, compound **8z** displayed moderate activity against *S. aureus* ATCC 25923 (MIC = 5 μM), suggesting a degree of selectivity toward this Gram-positive pathogen. These results indicate that the 4-aminoalkylquinoline scaffold primarily targets *M. tuberculosis*, with minimal activity against non-mycobacterial bacteria.

**Table 4:** Antibacterial Activity of Representative 4-Aminoalkylquinolines **8w** and **8z** against Gram-Positive and Gram-Negative Bacteria

entry	MIC _*A.baumannii*_ (μM)	MIC _*B. cereus*_ (μM)	MIC _*E. coli*_ (μM)	MIC_*E. faecium*_ (μM)	MIC _*S. aureus*_ (μM)	MIC _*K. neumoniae*_ (μM)
**8w**	>20	>20	>20	>20	20	>20
**8z**	>20	>20	>20	>20	5	>20

To further characterize the drug-like properties of this chemotype, 4-aminoalkylquinolines **8w** and **8z** were subjected to a panel of in vitro ADME assays (Table 5), including chemical stability, solubility, passive permeability, and metabolic stability. Collectively, these experiments provide an initial assessment of the physicochemical and metabolic liabilities associated with the series. First, chemical stability was evaluated under different pH conditions. In general, both compounds displayed greater stability under acidic conditions (pH 1.2), where molecule **8z** remained fully stable while **8w** retained 49% of its structure after incubation. In contrast, stability decreased markedly under neutral (pH 7.4) and basic (pH 9.1) conditions. This trend suggests that protonation of the amine functionality, likely associated with the hydrochloride form of the molecules, contributes to enhanced stability in acidic aqueous environments. Consistent with this observation, solubility also exhibited a pronounced pH dependence. Under acidic conditions, the bromo-substituted derivative **8z** showed higher solubility than the chloro-containing analogue **8w**. However, as the pH increased, the solubility of both compounds decreased substantially, indicating limited aqueous solubility at neutral and basic pH values. In addition to solubility, membrane permeability was investigated using the parallel artificial membrane permeability assay (PAMPA). Interestingly, structure **8z** displayed measurable passive permeation (2.3 × 10^–6^ cm/s), whereas **8w** remained essentially confined to the aqueous phase under the assay conditions. Although this permeability remains lower than that of the positive control alprenolol (9.2 × 10^–6^ cm/s), the result suggests that the bromine substituent may contribute favorably to membrane transport within this scaffold. Important to mention that **8z** showed enhanced passive permeability in the PAMPA assay compared with compound **2** (0.2 × 10^–6^ cm/s), supporting the original scaffold modification strategy. Finally, metabolic stability was evaluated using rat liver microsomes. Under these conditions, both derivatives exhibited relatively rapid metabolic turnover, with intrinsic clearance values of 58 and 62 mL/min/kg for **8w** and **8z**, respectively, and corresponding half-lives of 6.9 and 5.4 min. Notably, these values are comparable to those observed for the reference compound verapamil, indicating that this chemotype may be susceptible to hepatic metabolism.

**Table 5:** In Vitro ADME Profile of 4-Aminoalkylquinolines **8w** and **8z**

	chemical stability (%)^a^			solubility (μM)^b^					
entry	pH 1.2^b^	pH 7.4^c^	pH 9.1^d^	pH 1.2^c^	pH 7.4^d^	pH 9.1^e^	PAMPA (cm/s)	Cl_int_ ^e^ (mL/min/kg)	*t*_1/2_ ^f^ (min)
**8w**	49.0	8.0	4.0	8.99	0.51	0.24	0.0 × 10^–6^	58.0	6.9
**8z**	100	28.0	7.3	12.15	0.17	0.17	2.3 × 10^–6^	62.0	5.4

a Percentage of remaining compound after incubation at 37 °C for 24 h. Concentration of remaining compound after incubation at 25 °C for 4 h.

b 0.1 M HCl solution.

c PBS 1×.

d 0.1 M NH_4_HCO_3_ solution was used.

e Intrinsic clearance of rat liver microsomes.

f Half-life

Following the in vitro ADME characterization, the safety profile of the **8w** and **8z** was further investigated using an in vivo zebrafish embryo model (Table 6).^21^ Early evaluation of cardiotoxicity is a critical component of drug discovery, particularly for antitubercular agents, as several clinically used drugs—including bedaquiline and delamanid—have been associated with QT interval prolongation and increased risk of cardiac dysfunction.^2,3^ Consequently, zebrafish embryos provide a valuable platform for early toxicity screening due to their sensitivity to cardiotoxic effects and their suitability for rapid phenotypic assessment. Embryos were exposed to concentrations of 0.1, 0.3, and 1.0 μM of compounds **8w** and **8z**, and heart rate was measured at 2- and 5-days post fertilization (dpf). At 2 dpf, both compounds produced heart rates comparable to control groups at 0.1 and 0.3 μM, indicating an absence of detectable cardiotoxic effects at these concentrations. However, exposure to 1 μM resulted in a significant reduction in heart rate for both molecules. Consistent with this observation, embryos exposed to 1 μM did not survive to 5 dpf, indicating that this concentration exceeds the tolerated exposure range. Importantly, no alterations in heart rate were observed at 0.1 or 0.3 μM at either time point, whereas clear cardiotoxic effects emerged at 1.0 μM. Thus, the threshold for detectable cardiotoxicity lies above 0.3 μM under the conditions tested. Considering the MIC values of 0.03 μM for **8w** and 0.056 μM for **8z** against the H37Rv-LP strain, these data indicate an in vivo safety window of at least >10-fold and >5-fold, respectively. Furthermore, morphological evaluation revealed no significant changes in body length, ocular distance, or ocular surface area, and embryo survival remained unaffected at the tested submicromolar concentrations (data not shown). Taken together, these findings indicate that the 4-aminoalkylquinoline scaffold can maintain potent antimycobacterial activity while exhibiting a measurable therapeutic window in vivo.

**Table 6:** Zebrafish Heart Rate Assessment of 4-Aminoalkylquinolines **8w** and **8z**

entry	control	0.5% DMSO	0.1 μM	0.3 μM	1 μM
Zebrafish Heart Rate (Mean ± SD/min) – Embryos 2 dpf					
**8w**	125.9 ± 22.1	125.4 ± 21.5	130.1 ± 25.5	125.2 ± 24.0	83.3 ± 17.1^a^/^b^
**8z**	125.9 ± 22.1	125.4 ± 21.5	127.9 ± 25.1	125.8 ± 24.1	82.3 ± 26.2^a^/^b^
Zebrafish Heart Rate (Mean ± SD/min) – Embryos 5 dpf					
**8w**	175.9 ± 10.3	179.1 ± 5.8	177.5 ± 4.4	174.8 ± 7.5	-
**8z**	175.9 ± 10.3	179.1 ± 5.8	176.5 ± 5.3	174.0 ± 9.5	-

a ****P < 0.0001 compared with control group (Tukey post-test).

b ^####^ *P* < 0.01 compared with the 1% DMSO group (Tukey post-test).

In summary, this study identifies 4-aminoalkylquinolines as a promising chemotype for antitubercular drug discovery. Structure–activity relationship studies revealed key contributions of alkyl substitution at C-2 and hydrophobic substituents on the terminal phenyl ring for optimal potency. The most active derivatives displayed submicromolar to low-nanomolar activity against *M. tuberculosis* in both the H37Rv and H37Rv-LP assays and retained strong inhibitory activity against multidrug-resistant clinical isolates. Mechanistic studies using the QcrB_T313I_ mutant indicated a consistent reduction in potency, supporting inhibition of the cytochrome bc_1_ complex as the likely molecular target. Importantly, the most active compounds showed favorable selectivity indices in mammalian Vero and HepG2 cells and limited antibacterial activity against non-mycobacterial species. In vitro ADME profiling revealed moderate membrane permeability and rapid metabolic turnover, while zebrafish cardiotoxicity studies demonstrated a measurable in vivo safety window relative to antimycobacterial potency. Importantly, structural assignment was further supported by single-crystal X-ray diffraction. Collectively, these results highlight the 4-aminoalkylquinoline scaffold as a promising platform for the development of new inhibitors targeting mycobacterial respiration and provide a foundation for further optimization in tuberculosis drug discovery campaigns.

## Supplementary Material



## Notes

Synthetic and biological procedures, analytical and crystallographic data (PDF)

## ∇

(T.P.) Liverpool School of Tropical Medicine, Liverpool, UK
