# Supplementary material for: 4‑Aminoalkylquinolines as Potent Antitubercular Agents Targeting the Cytochrome bc1 Complex
Source: ACS Med Chem Lett. 2026 May 15;17(6):1384–92. doi: 10.1021/acsmedchemlett.6c00183 (PMC13266640; doi:10.1021/acsmedchemlett.6c00183)
Supplement: Supplementary file 1 [file ml6c00183_si_002.pdf]

Support Information

4-Aminoalkylquinolines as Potent Antitubercular Agents Targeting the Cytochrome bc_1 Complex

Estevão Silveira Grams, Alessandro Silva Ramos, Fernanda Fries da Silva, Marcia Alberton Perelló, Alexia de Matos Czeczot, Ariel Moura Maia, Higor Arruda Caetano, Stefani Altenhofen, Josiane Delgado Paz, Guilherme Arraché Gonçalves, Xinyi Grace Liuh, Teresa Repasyh, Luisa Dreher Klein, Gabriela Pagnoncelli Polesello, Carlos Alexandre Sanchez Ferreira, Sílvia Dias de Oliveira, Rafael Stieler, Sidnei Moura e Silva, Carla Denise Bonan, Cristiano Valim Bizarro, Luiz Augusto Basso, Tanya Parish, Pablo Machado*

Table of contents:

1. Experimental Section
2. Spectroscopic and spectrometric data
3. Biological procedures
4. ^{1}H and ^{13}C spectra of synthesized compounds
5. Crystallographic data for **8z**

1. Experimental Section

1.1 *Chemistry section*

All solvents, reactants and reagents, including the 6-bromo-4-hydroxyquinoline **9a** precursor, were obtained from commercial sources and used without further purification. The elucidation process of the chemical structure synthesized by NMR analysis performed in an Avance III HD Bruker spectrometer (Fällanden, Switzerland), which the ^{1}H and ^{13}C spectra were obtained. The chemical shifts were expressed by in parts per million (ppm) relative to DMSO-d_6 or $CDCl_3$, as the solvent, and to TMS, as the internal standard. High-resolution mass spectra (HRMS) analyses were performed on Bruker MicroTOF-QII using electrospray ionization (ESI) (University of Caxias do Sul, Brazil).The melting points were measured using a Microquímica MQAPF- 302 equipment. The compounds' purities were determined using a Dionex Ultimate 3000 UHPLC chromatograph (Dionex Corporation, Germering, Germany). Chromeleon software (version 6.80 SR11 Build 3160 (183147)) was used for data acquisition and processing. The liquid chromatography conditions were as follows: RP column, 5 mm Nucleodur C-18ec (250 × 4.6 mm); flow rate, 1.5 mL/min; UV detection, 260–280 nm; 100% water (0.1% acetic acid) was maintained from 0 to 7 min, followed by a linear gradient from 100% water (0.1% acetic acid) to 90% acetonitrile/ methanol (1:1, v/v) from 7 to 15 min (15–30 min), and subsequently returned to 100% water (0.1% acetic acid) in 5 min (30–35 min) and maintained for an additional 10 min (35–45 min). All the evaluated compounds were ≥ 95% pure. It is essential to note that no unexpected or unusually high safety hazards were encountered during the synthetic procedures.

1.2 General procedure for the synthesis of 4-hydroxyquinolines (**5a**–**f**).

The synthesis of 2-alkyl-4-hydroxyquinolines was performed using the reaction between the substituted anilines (25 mmol) and *β*-ketoesters (29.4 mmol) in the presence of magnesium sulfate (30 mmol) and acetic acid (7.5 mmol) using ethanol (30 mL) as solvent. The mixture was heated under stirring at 90 °C for 16 h. Subsequently, the magnesium sulfate was removed by filtration and the ethanol was evaporated under reduced pressure providing the corresponding intermediate-acrylate. Thermal cyclization of the intermediary was performed by heating in Dowtherm® A (30 mL) at a temperature range of 230–250 °C for 15 minutes. Then, the reactional mixture formed was washed with hexane (100 mL). Finally, the solid formed was washed with chloroform (100 mL) and then dried under reduced pressure.[1]

1.3 General procedure for the synthesis of 4-chloroquinolines (**6a**–**f**).

In a two neck round bottom flask containing the 4-hydroxyquinoline (1 mmol) dissolved in toluene (10 mL) was added phosphorus (V) oxychloride ($POCl_3$) (2.5 mmol) also dissolved in toluene. The reaction was kept at 110 °C under stirring for 2 h. After cooling, excess $POCl_3$ and solvent were removed under reduced pressure. Finally, the reaction mixture was neutralized with saturated sodium bicarbonate (60 mL). The product was extracted with ethyl acetate (3x30 mL), the organic phase was dried by magnesium sulfate, filtered, and concentrated under reduced pressure. The solvent was evaporated on reduced pressure and the product was purified on silica gel column chromatography (hexane:ethyl acetate 80:20).[1]

1.4 General procedure for the synthesis of (**8a**–**z; 10a**–**c**).

First the 4-chloroquinolines were obtained according to above chlorination method. Afterward, in a Schlenk tube were added 2-alkyl-4-chloroquinoline (1.0 mmol) *N,N*-diisopropylethylamine (DIPEA) (3.0 mmol), dimethylsulfoxide (5 mL), and 3-Phenylpropan-1-amines of interest (1.4 mmol). The reaction was heated at a temperature of 150 °C for 20 h. After cooling, the product was extracted with ethyl acetate (3x30 mL). The organic phase was neutralized with saturated sodium bicarbonate (2x30mL), then, the organic phase was washed with brine (3x50 mL), dried over magnesium sulfate and the solvent removed under vacuum system. The product was purified on silica gel column chromatography with hexane:ethyl acetate (70:30 to 0:100). For some compounds, the eluent used was ethyl acetate:methanol (100:0 to 94:6), follow by recristalization with hexane pure.[2]

1.4 General procedure for the synthesis of (**11a**–**c**).

The 4-aminoalkylquinolines **11a**–**c** were synthesized according to reported protocol.[3] The first reaction step was carried out in a round-bottom flask containing the respective 4-hydroxyquinoline (2 mmol), 1,3-dibromopropane (1.21 g, 6 mmol), cesium carbonate (1.95 g, 6 mmol), sodium iodide (0.075 g, 0.5 mmol) in acetonitrile (50 mL). The reaction mixture was stirred at room temperature (25 °C) for 24 h. Subsequently, extraction was performed using chloroform (3 × 50 mL) and saturated ammonium chloride solution. The organic layer was dried over magnesium sulfate and then concentrated under reduced pressure. The purification of 4-bromoalkoxyquinolines **11a**–**d** was accomplished by chromatography using silica gel as the stationary phase and a mobile phase consisting of either 100% ethyl acetate or a mixture of ethyl acetate and hexane in a 3:7 ratio.

In the second reaction step the desired compounds were synthesized from alkylation involving appropriately substituted anilines. The reactions were conducted in a round-bottom flasks containing the 4-bromoalkoxyquinoline of interest (1 mmol), the respective aniline (2 mmol), potassium carbonate (0.41 g, 3 mmol), sodium iodide (0.15 g, 1 mmol), and toluene (20 mL). The reaction mixture was heated at 110 °C for 48-72 h. Afterward, extraction was performed using chloroform (3x50 mL) and saturated ammonium chloride solution. The organic layer was dried over magnesium sulfate and then concentrated under reduced pressure. The purification of the products was conducted employing chromatographic separation with silica gel as the stationary phase and a mobile phase comprising mixtures of hexane and ethyl acetate in ratios of 1:9 and 3:7 or recrystallization from ethyl acetate.

6-Chloro-2-methyl-*N*-(3-phenylpropyl)quinolin-4-amine (**8a**)

Column chromatography on silica gel hexane – Ethyl acetate (6:4), light yellow solid, Yield: 47%; mp.:118-119 °C; HPLC: 99% (t_R = 14.68 min); ^{1}H NMR (400 MHz, DMSO-d_6) *δ* 1.98 (p, *J* = 7.7 Hz, 2H), 2.44 (s, 3H), 2.72 (t, *J* = 7.6 Hz, 2H), 3.20 – 3.30 (m, 2H), 6.29 (s, 1H), 7.12 (t, *J* = 5.3 Hz, 1H), 7.17 – 7.23 (m, 1H), 7.23 – 7.35 (m, 4H), f7.55 (dd, *J* = 8.9, 2.3 Hz, 1H), 7.69 (d, *J* = 8.9 Hz, 1H), 8.33 (d, *J* = 2.3 Hz, 1H); ^{13}C NMR (101 MHz, DMSO-d_6) δ 25.1, 29.4, 32.6, 41.8, 98.6, 118.3, 120.8, 125.7, 127.4, 128.2 (2C), 128.3 (2C), 128.9, 130.3, 141.6, 146.6, 149.3, 159.2; FTMS (ESI) m/z: 311.1302 [M+H]+; calc for $C_{19}H_{20}ClN_2$: 311.1310.

6-Bromo-2-methyl-*N*-(3-phenylpropyl) quinolin-4-amine (**8b**)

Column chromatography on silica gel hexane – Ethyl acetate (1:1), white ice solid, Yield: 53%; mp.: 125-126 °C; HPLC: 98% (t_R = 14.82 min); ^{1}H NMR (400 MHz, Chloroform-*d*) *δ* 2.11 (p, *J* = 7.0 Hz, 2H), 2.56 (s, 3H), 2.81 (t, *J* = 7.2 Hz, 2H), 3.27 – 3.36 (m, 2H), 4.72 (s, 1H), 6.23 (s, 1H), 7.21 – 7.29 (m, 3H), 7.31 – 7.38 (m, 2H), 7.54 (d, *J* = 2.2 Hz, 1H), 7.61 (dd, *J* = 8.9, 2.1 Hz, 1H), 7.74 (d, *J* = 8.9 Hz, 1H); ^{13}C NMR (101 MHz, Chloroform-*d*) *δ* 25.7, 30.0, 33.7, 43.0, 99.6, 117.3, 118.7, 121.9, 126.6, 128.4 (2C), 128.8 (2C), 130.9, 132.2, 141.1, 147.0, 148.7, 160.0; FTMS (ESI) m/z: 355.0802 [M+H]+; calc for $C_{19}H_{20}BrN_2$: 355.0804.

6-Iodo-2-methyl-*N*-(3-phenylpropyl)quinolin-4-amine (**8c**)

Column chromatography on silica gel hexane – Ethyl acetate (6:4), white ice solid, Yield: 29%; mp.: 125–126 °C; HPLC: 97% (t_R =15.40 min); ^{1}H NMR (400 MHz, Chloroform-*d*) *δ* 2.11 (t, *J* = 7.0 Hz, 2H), 2.55 (s, 3H), 2.81 (t, *J* = 7.2 Hz, 2H), 3.31 (s, 2H), 4.76 (s, 1H), 6.22 (s, 1H), 7.21 – 7.28 (m, 3H), 7.29 – 7.36 (m, 2H), 7.60 (d, *J* = 8.7 Hz, 1H), 7.75 – 7.80 (m, 2H); ^{13}C NMR (101 MHz, Chloroform-*d*) *δ* 25.7, 30.0, 33.7, 43.0, 88.2, 99.5, 119.5, 126.6, 128.3, 128.4 (2C), 128.8 (2C), 130.9, 137.6, 141.1, 147.3, 148.5, 160.2; FTMS (ESI) m/z: 403.0675 [M+H]+; calc for $C_{19}H_{20}IN_2$: 403.0666.

6-Methoxy-2-methyl-*N*-(3-phenylpropyl)quinolin-4-amine (**8d**)

Column chromatography on silica gel hexane – Ethyl acetate (2:8), pastel yellow solid Yield: 58%; mp.: 138-139 °C; UHPLC: 98% (t_R =14.67 min); ^{1}H NMR (400 MHz, DMSO-d_6) *δ* 1.99 (p, *J* = 7.7 Hz, 2H), 2.43 (s, 3H), 2.73 (t, *J* = 7.6 Hz, 2H),

3.29 (q, J = 6.7 Hz, 2H), 3.87 (s, 3H), 6.25 (s, 1H), 7.13 (t, J = 4.9 Hz, 1H), 7.16 – 7.35 (m, 6H), 7.59 (d, J = 2.9 Hz, 1H), 7.64 (d, J = 9.1 Hz, 1H); ^{13}C NMR (101 MHz, DMSO-d_6) δ 24.4, 29.6, 32.6, 41.9, 55.6, 98.0, 101.2, 117.7, 120.2, 125.7, 128.2 (2C), 128.3 (2C), 128.8, 141.6, 142.5, 149.6, 155.5, 155.6; FTMS (ESI) m/z: 307.1786 [M+H]+; calc for $C_{20}H_{23}N_2O$: 307.1805.

6-Chloro-*N*-(3-(4-methoxyphenyl)propyl)-2-methylquinolin-4-amine (**8e**)

Column chromatography on silica gel hexane – Ethyl acetate (3:7) followed by recrystallisation from hexane, off-white solid. Yield: 35%; mp.: 120-121 °C; UHPLC: 95% (t_R =14.84 min); ^{1}H NMR (400 MHz, DMSO-d_6) δ 1.94 (p, J = 8.4, 7.8 Hz, 2H), 2.43 (s, 3H), 2.65 (t, J = 7.6 Hz, 2H), 3.23 (q, J = 6.5 Hz, 2H), 3.72 (s, 3H), 6.28 (s, 1H), 6.83 – 6.90 (m, 2H), 7.12 (t, J = 5.4 Hz, 1H), 7.14 – 7.20 (m, 2H), 7.55 (dd, J = 9.1, 2.3 Hz, 1H), 7.69 (d, J = 9.0 Hz, 1H), 8.33 (d, J = 2.4 Hz, 1H); ^{13}C NMR (101 MHz, DMSO-d_6) δ 25.1, 29.6, 31.7, 41.8, 54.9, 98.6, 113.7 (2C), 118.3, 120.8, 127.4, 128.9, 129.2 (2C), 130.3, 133.4, 146.5, 149.4, 157.4, 159.2; FTMS (ESI) m/z: 341.1417 [M+H]+; calc for $C_{20}H_{22}ClN_2O$: 341.1415.

6-Bromo-*N*-(3-(4-methoxyphenyl)propyl)-2-methylquinolin-4-amine (**8f**)

Column chromatography on silica gel hexane – Ethyl acetate (3:7) followed by recrystallisation by n-hexane, off-white solid. Yield: 33%; mp.:116-117 °C; HPLC: 99% (t_R =15.63 min); ^{1}H NMR (400 MHz, DMSO-d_6) δ 1.94 (p, J = 7.3 Hz, 2H), 2.43 (s, 3H), 2.65 (t, J = 7.5 Hz, 2H), 3.23 (q, J = 6.7 Hz, 2H), 3.72 (s, 3H), 6.28 (s, 1H), 6.82 – 6.90 (m, 2H), 7.10 – 7.21 (m, 3H), 7.58 – 7.69 (m, 2H), 8.47 (d, J = 2.1 Hz, 1H); ^{13}C NMR (101 MHz, DMSO-d_6) δ 25.1, 29.6, 31.7, 41.8, 54.9, 98.6, 113.6

(2C), 115.7, 118.9, 123.9, 129.2 (2C), 130.4, 131.5, 133.4, 146.7, 149.2, 157.4, 159.3; FTMS (ESI) m/z: 385.0909 [M+H]+; calc for $C_{20}H_{22}BrN_2O$: 385.0910.

6-Iodo-*N*-(3-(4-methoxyphenyl)propyl)-2-methylquinolin-4-amine (**8g**)

Column chromatography on silica gel hexane – Ethyl acetate (2:8) followed by recrystallisation from hexane, off-white solid. Yield: 23%; mp.: 116–117 °C; UHPLC: 96% (t_R =15.68 min); ^{1}H NMR (400 MHz, Chloroform-*d*) δ 2.07 (p, J = 7.2 Hz, 2H), 2.56 (s, 3H), 2.74 (t, J = 7.3 Hz, 2H), 3.30 (q, J = 6.5 Hz, 2H), 3.79 (s, 3H), 4.85 (s, 1H), 6.22 (s, 1H), 6.86 (d, J = 8.6 Hz, 2H), 7.14 (d, J = 8.3 Hz, 2H), 7.60 (d, J = 8.9 Hz, 1H), 7.78 (dd, J = 8.9, 1.8 Hz, 1H), 7.86 (d, J = 1.6 Hz, 1H); ^{13}C NMR (101 MHz, Chloroform-*d*) δ 25.7, 30.2, 32.6, 42.8, 55.3, 88.2, 99.5, 114.1 (2C), 119.5, 128.4, 129.3 (2C), 130.9, 133.0, 137.6, 147.2, 148.5, 158.2, 160.1 ; FTMS (ESI) m/z: 433.0779 [M+H]+; calc for$C_{20}H_{22}IN_2O$: 433.0771.

6-Methoxy-*N*-(3-(4-methoxyphenyl)propyl)-2-methylquinolin-4-amine (**8h**)

Column chromatography on silica gel hexane – Ethyl acetate (2:8) followed by recrystallisation from hexane, off-white solid. Yield: 32%; mp.: 107–108 °C; UHPLC: 95% (t_R =14.42 min); ^{1}H NMR (400 MHz, Chloroform-*d*) δ 2.06 (p, J = 7.2, 5.6 Hz, 2H), 2.56 (s, 3H), 2.73 (t, J = 7.3 Hz, 2H), 3.31 (q, J = 6.7 Hz, 2H), 3.78 (s, 3H), 3.83 (s, 3H), 4.87 (s, 1H), 6.23 (s, 1H), 6.78 – 6.89 (m, 4H), 7.13 (d, J = 8.4 Hz, 1H), 7.24 (dd, J = 9.2, 2.6 Hz, 1H), 7.83 (d, J = 9.2 Hz, 1H); ^{13}C NMR (101 MHz, Chloroform-*d*) δ 25.4, 30.4, 32.6, 42.8, 55.2, 55.6, 74.8 – 79.6 (m), 77.3, 99.2, 99.3, 114.0 (2C), 117.8, 120.0, 129.3 (2C), 130.5, 133.2, 143.8, 148.9, 156.3, 157.0, 158.1; FTMS (ESI) m/z: 337.1920 [M+H]+; calc for $C_{21}H_{25}N_2O_2$: 337.1911.

6-Chloro-2-methyl-*N*-(3-(*p*-tolyl)propyl)quinolin-4-amine (**8i**)

Yield: 36%; mp.: 108-109 °C; UHPLC: 95% (t_R =15.45 min); ^{1}H NMR (400 MHz, Chloroform-*d*) *δ* 2.09 (p, *J* = 7.0 Hz, 2H), 2.35 (s, 3H), 2.55 (s, 3H), 2.77 (t, *J* = 7.2 Hz, 2H), 3.31 (q, *J* = 6.7 Hz, 2H), 4.78 (s, 1H), 6.22 (s, 1H), 7.13 (d, *J* = 1.8 Hz, 4H), 7.60 (d, *J* = 8.8 Hz, 1H), 7.75 – 7.84 (m, 2H); ^{13}C NMR (101 MHz, Chloroform-*d*) δ 21.2, 25.7, 30.1, 33.2, 42.9, 88.2, 99.5, 119.5, 128.3 (2C), 128.4, 129.4 (2C), 130.9, 135.9, 137.6, 137.9, 147.2, 148.5, 160.1; FTMS (ESI) m/z: 325.1462 [M+H]+; calc for $C_{20}H_{22}ClN_2$: 325.1466.

6-Bromo-2-methyl-*N*-(3-(p-tolyl)propyl)quinolin-4-amine (**8j**)

Column chromatography on silica gel hexane – Ethyl acetate (1:1), white ice solid Yield: 33%; mp.: 121–122 °C; UHPLC: 95% (t_R = 15.36 min); ^{1}H NMR (400 MHz, Chloroform-*d*) *δ* 2.09 (p, *J* = 7.1 Hz, 2H), 2.34 (s, 3H), 2.55 (s, 3H), 2.77 (t, *J* = 7.2 Hz, 2H), 3.31 (q, *J* = 6.5 Hz, 2H), 4.73 (s, 1H), 6.22 (s, 1H), 7.11 (d, *J* = 8.6 Hz, 4H), 7.56 (d, *J* = 2.1 Hz, 1H), 7.61 (dd, *J* = 8.9, 2.1 Hz, 1H), 7.74 (d, *J* = 8.9 Hz, 1H); ^{13}C NMR (101 MHz, Chloroform-*d*) *δ* 21.1, 25.7, 30.0, 33.2, 43.0, 99.5, 117.2, 118.7, 121.9, 128.3 (2C), 129.4 (2C), 130.9, 132.2, 135.9, 138.0, 146.9, 148.7, 160.0.; FTMS (ESI) m/z: 369.0961 [M+H]+; calc for $C_{20}H_{22}BrN_2$: 369.0961.

6-Iodo-2-methyl-*N*-(3-(*p*-tolyl)propyl)quinolin-4-amine (**8k**)

Column chromatography on silica gel hexane – Ethyl acetate (2:8) followed by recrystallisation from hexane, off-white solid. Yield: 23%; mp.: 119-120 °◦C;

UHPLC: 97% (t_R =15.78 min); ^{1}H NMR (400 MHz, Chloroform-*d*) δ 2.09 (p, *J* = 7.0 Hz, 2H), 2.35 (s, 3H), 2.55 (s, 3H), 2.77 (t, *J* = 7.2 Hz, 2H), 3.31 (q, *J* = 6.7 Hz, 2H), 4.78 (s, 1H), 6.22 (s, 1H), 7.08 – 7.17 (m, 4H), 7.60 (d, *J* = 8.8 Hz, 1H), 7.78 (dd, *J* = 8.8, 1.9 Hz, 1H), 7.82 (d, *J* = 1.9 Hz, 1H); ^{13}C NMR (101 MHz, Chloroform-*d*) *δ* 21.2, 25.7, 30.1, 33.2, 42.9, 88.2, 99.5, 119.5, 128.3 (2C), 128.4, 129.4 (2C), 130.9, 135.9, 137.6, 137.9, 147.2, 148.5, 160.1. FTMS (ESI) m/z: 417.0828 [M+H]+; calc for $C_{20}H_{22}IN_2$: 417.0822.

6-Methoxy-2-methyl-*N*-(3-(*p*-tolyl)propyl)quinolin-4-amine (**8l**):

Column chromatography on silica gel hexane – Ethyl acetate (3:7) followed by recrystallisation from hexane, off-white solid. Yield: 37%; mp.: 136 – 137 °C; UHPLC: 95% (t_R = 14.62 min); ^{1}H NMR (400 MHz, Chloroform-*d*) *δ* 2.08 (p, *J* = 7.2 Hz, 2H), 2.33 (s, 3H), 2.56 (s, 3H), 2.76 (t, *J* = 7.3 Hz, 2H), 3.32 (q, *J* = 6.6 Hz, 2H), 3.85 (s, 3H), 4.76 (s, 1H), 6.23 (s, 1H), 6.82 (d, *J* = 2.7 Hz, 1H), 7.12 (s, 4H), 7.21 – 7.29 (m, 1H), 7.83 (d, *J* = 9.2 Hz, 1H); ^{13}C NMR (101 MHz, Chloroform-*d*) δ 21.0, 25.4, 30.3, 33.1, 42.9, 55.6, 99.3, 99.3, 117.8, 119.9, 128.3 (2C), 129.3 (2C), 130.6, 135.7, 138.1, 143.9, 148.9, 156.3, 157.1; FTMS (ESI) m/z: 321.1969 [M+H]+; calc for $C_{21}H_{25}N_2O$: 321.1961.

6-Chloro-*N*-(3-(4-isopropylphenyl)propyl)-2-methylquinolin-4-amine (**8m**):

Column chromatography on silica gel hexane – Ethyl acetate (1:1) followed by recrystallisation from hexane, off-white solid. Yield:44%; mp.: 117-118 °C; UHPLC: 96% (t_R =15.58 min); ^{1}H NMR (400 MHz, Chloroform-*d*) *δ* 1.2 (d, *J* = 6.9 Hz, 6H), 2.1 (p, *J* = 7.1 Hz, 2H), 2.6 (s, 3H), 2.8 (t, *J* = 7.3 Hz, 2H), 2.9 (hept, *J* = 6.2 Hz,

1H), 3.3 (q, *J* = 6.6 Hz, 2H), 4.8 (s, 1H), 6.2 (s, 1H), 7.2 (q, *J* = 8.0 Hz, 4H), 7.5 (d, *J* = 8.5 Hz, 2H), 7.8 (d, *J* = 8.8 Hz, 1H); ^{13}C NMR (101 MHz, Chloroform-*d*) *δ* 24.0 (2C), 25.7, 30.1, 33.0, 33.7, 42.8, 99.6, 118.2, 118.6, 126.7 (2C), 128.3 (2C), 129.4, 129.5, 129.6, 130.8, 138.3, 146.7, 146.9, 148.8, 159.8.; FTMS (ESI) m/z:353.1770 [M+H]+; calc for $C_{22}H_{26}ClN_2$: 353.1779.

6-Bromo-*N*-(3-(4-isopropylphenyl)propyl)-2-methylquinolin-4-amine (**8n**):

Column chromatography on silica gel hexane – Ethyl acetate (4:6) followed by recrystallisation from hexane, off-white solid. Yield: 36%; mp.: 121–122 °C; UHPLC: 99% (t_R =15.48 min); ^{1}H NMR (400 MHz, DMSO-*d*6) *δ* 1.18 (d, *J* = 6.2 Hz, 6H), 1.95 (p, *J* = 7.4 Hz, 2H), 2.42 (s, 3H), 2.67 (t, *J* = 7.7 Hz, 2H), 2.84 (p, *J* = 7.1 Hz, 1H), 3.24 (q, *J* = 6.7 Hz, 2H), 6.28 (s, 1H), 7.12 – 7.18 (m, 5H), 7.58 – 7.69 (m, 2H), 8.47 (s, 1H); ^{13}C NMR (101 MHz, DMSO-d_6) δ 23.87, 25.10, 29.39, 32.15, 32.94, 41.82, 98.57, 115.69, 118.90, 123.91, 126.08 (2C), 128.17 (2C), 130.46, 131.46, 138.87, 145.68, 146.75, 149.22, 159.32; FTMS (ESI) m/z: 397.1267 [M+H]+; calc for $C_{22}H_{26}BrN_2$: 397.1274.

6-Iodo-*N*-(3-(4-isopropylphenyl)propyl)-2-methylquinolin-4-amine (**8o**):

Column chromatography on silica gel hexane – Ethyl acetate (1:1) followed by recrystallisation from hexane, off-white solid. Yield: 25%; mp.: 119–120 °C; UHPLC: 98% (t_R =16.12 min); ^{1}H NMR (400 MHz, Chloroform-*d*) *δ* 1.2 (s, 3H), 1.2 (s, 3H), 2.1 (t, *J* = 7.4 Hz, 2H), 2.6 (s, 3H), 2.7 (t, *J* = 7.3 Hz, 2H), 2.9 (p, *J* = 6.9 Hz, 1H), 3.4 (d, *J* = 7.5 Hz, 2H), 6.0 (s, 1H), 7.1 (d, *J* = 3.6 Hz, 4H), 7.7 (dd, *J* = 8.8, 1.7 Hz, 1H), 7.8 (d, *J* = 8.8 Hz, 1H), 8.7 – 8.7 (m, 1H); ^{13}C NMR (101 MHz,

Chloroform-*d*) δ 22.3, 24.0 (2C), 29.5, 32.7, 33.7, 42.9, 89.7, 98.5, 118.4, 124.8, 126.6 (2C), 128.4 (2C), 131.0, 138.1, 139.9, 140.8, 146.9, 152.1, 155.8 ; FTMS (ESI) m/z: 445.1140 [M+H]+; calc for $C_{22}H_{26}IN_2$: 445.1135.

N-(3-(4-(Tert-butyl)phenyl)propyl)-6-chloro-2-methylquinolin-4-amine (**8p**):

Column chromatography on silica gel hexane – Ethyl acetate (1:1) followed by recrystallisation from hexane, off-white solid. Yield: 26%; mp.: 115-116 °C; UHPLC: 97% (t_R = 15.94 min); ^{1}H NMR (400 MHz, Chloroform-*d*) δ 1.32 (s, 9H), 2.09 (p, *J* = 7.1 Hz, 2H), 2.57 (s, 3H), 2.77 (t, *J* = 7.4 Hz, 2H), 3.32 (q, *J* = 6.6 Hz, 2H), 4.82 (s, 1H), 6.25 (s, 1H), 7.16 (d, *J* = 8.0 Hz, 2H), 7.34 (d, *J* = 7.9 Hz, 2H), 7.49 (dd, *J* = 8.9, 2.2 Hz, 1H), 7.56 (d, *J* = 2.2 Hz, 1H), 7.82 (d, *J* = 8.9 Hz, 1H); ^{13}C NMR (101 MHz, Chloroform-*d*) δ 25.7, 30.1, 31.4 (3C), 32.8, 34.4, 42.8, 99.7, 118.2, 118.6, 125.5 (2C), 128.1 (2C), 129.4, 129.6, 130.8, 137.9, 146.7, 148.8, 149.2, 159.9. FTMS (ESI) m/z: 367.1937 [M+H]+; calc for $C_{23}H_{28}N_2O$: 367.1936.

6-Bromo-*N*-(3-(4-(tert-butyl)phenyl)propyl)-2-methylquinolin-4-amine (**8q**):

Column chromatography on silica gel hexane – Ethyl acetate (1:1) followed by recrystallisation from hexane, off-white solid. Yield: 27%; mp.: 121-122 °C; UHPLC: 96% (t_R =15.80 min); ^{1}H NMR (400 MHz, Chloroform-*d*) δ 1.32 (s, 9H), 2.08 (p, *J* = 7.3 Hz, 2H), 2.56 (s, 3H), 2.77 (t, *J* = 7.4 Hz, 2H), 3.31 (q, *J* = 6.7 Hz, 2H), 4.86 (s, 1H), 6.25 (s, 1H), 7.16 (d, *J* = 7.9 Hz, 2H), 7.34 (d, *J* = 8.1 Hz, 2H), 7.62 (dd, *J* = 9.0, 2.0 Hz, 1H), 7.71 – 7.79 (m, 2H); ^{13}C NMR (101 MHz, Chloroform-*d*) δ 25.7, 30.1, 31.4 (3C), 32.8, 34.4, 42.8, 99.7, 117.3, 118.8, 121.9, 125.5 (2C), 128.1 (2C), 130.9, 132.3, 137.9, 146.9, 148.8, 149.2, 160.0; FTMS (ESI) m/z: 411.1427 [M+H]+; calc for $C_{23}H_{28}BrN_2$: 411.1430.

N-(3-(4-(tert-butyl)phenyl)propyl)-6-iodo-2-methylquinolin-4-amine (**8r**):

Column chromatography on silica gel hexane – Ethyl acetate (1:1) followed by recrystallisation from hexane, off-white solid. Yield: 22%; mp.: 116-117 °C; UHPLC: 98% (t_R =16.20 min); ^{1}H NMR (400 MHz, Chloroform-*d*) *δ* 1.32 (s, 9H), 2.09 (p, *J* = 7.3 Hz, 2H), 2.56 (s, 3H), 2.77 (t, *J* = 7.4 Hz, 2H), 3.31 (q, *J* = 6.7 Hz, 2H), 4.90 (s, 1H), 6.23 (s, 1H), 7.16 (d, *J* = 8.0 Hz, 2H), 7.34 (d, *J* = 8.2 Hz, 2H), 7.61 (d, *J* = 8.9 Hz, 1H), 7.76 – 7.82 (m, 1H), 7.97 (d, *J* = 1.5 Hz, 1H); ^{13}C NMR (101 MHz, Chloroform-*d*) *δ* 25.6, 30.1, 31.4 (3C), 32.7, 34.4, 42.7, 88.2, 99.6, 119.5, 125.5 (2C), 128.0 (2C), 128.4, 130.9, 137.6, 137.8, 147.2, 148.5, 149.2, 160.1; FTMS (ESI) m/z: 459.1290 [M+H]+; calc for $C_{23}H_{28}IN_2$: 459.1292.

N-(3-(4-(Tert-butyl)phenyl)propyl)-6-methoxy-2-methylquinolin-4-amine (**8s**):

Column chromatography on silica gel hexane – Ethyl acetate (3:7) followed by recrystallization by n-hexane, white solid. Yield: 33%; mp.: 118–119 °C; UHPLC: 96% (t_R =15.89 min); ^{1}H NMR (400 MHz, Chloroform-*d*) *δ* ^{1}H NMR (400 MHz, Chloroform-*d*) δ 1.31 (s, 9H), 2.09 (p, *J* = 7.4 Hz, 2H), 2.56 (s, 3H), 2.76 (t, *J* = 7.5 Hz, 2H), 3.34 (q, *J* = 6.7 Hz, 2H), 3.86 (s, 3H), 4.86 (s, 1H), 6.24 (s, 1H), 6.90 (d, *J* = 2.7 Hz, 1H), 7.16 (d, *J* = 7.9 Hz, 2H), 7.23 – 7.27 (m, 1H), 7.33 (d, *J* = 8.0 Hz, 2H), 7.84 (d, *J* = 9.2 Hz, 1H); ^{13}C NMR (101 MHz, Chloroform-*d*) *δ* 25.4, 30.3, 31.4 (3C), 32.8, 34.4, 42.8, 55.6, 99.3, 99.4, 117.8, 119.9, 125.5 (2C), 128.1 (2C), 130.6, 138.1, 143.8, 148.9, 149.1, 156.3, 157.1; FTMS (ESI) m/z: 363.2431 [M+H]+; calc for $C_{24}H_{31}N_2O$: 363.2431.

6-Chloro-*N*-(3-(4-chlorophenyl)propyl)-2-methylquinolin-4-amine (**8t**):

Column chromatography on silica gel hexane – Ethyl acetate (4:6) followed by recrystallization from hexane, off-white solid. Yield: 41%; mp.: 124-125 °C; UHPLC: 95% (t_R =15.46 min); ^{1}H NMR (400 MHz, DMSO-d_6) 1.96 (p, *J* = 7.6 Hz, 2H), 2.44 (s, 3H), 2.72 (t, *J* = 7.8 Hz, 2H), 3.24 (q, *J* = 6.6 Hz, 2H), 6.30 (s, 1H), 7.12 (t, *J* = 5.3 Hz, 1H), 7.25 – 7.39 (m, 4H), 7.55 (dd, *J* = 8.9, 2.3 Hz, 1H), 7.69 (d, *J* = 8.9 Hz, 1H), 8.32 (d, *J* = 2.4 Hz, 1H); ^{13}C NMR (101 MHz, DMSO-d_6) δ 25.1, 29.2, 31.8, 41.7, 98.6, 118.3, 120.8, 127.4, 128.1 (2C), 128.9, 130.2 (2C), 130.3, 130.3, 140.6, 146.5, 149.3, 159.2 ; FTMS (ESI) m/z: 345.0907 [M+H]+; calc for $C_{19}H_{19}Cl_2N_2$: 345.0920.

6-Bromo-*N*-(3-(4-chlorophenyl)propyl)-2-methylquinolin-4-amine (**8u**):

Column chromatography on silica gel hexane – Ethyl acetate (1:1) followed by recrystallization from hexane, off-white solid. Yield: 33%; mp.: 117-118 °C; UHPLC: 96% (t_R =15.05 min); ^{1}H NMR (400 MHz, DMSO-d_6) *δ* 2.0 (p, *J* = 7.3 Hz, 2H), 2.43 (s, 3H), 2.7 (t, *J* = 7.6 Hz, 2H), 3.2 (t, *J* = 6.1 Hz, 2H), 6.29 (s, 1H), 7.14 (t, *J* = 5.3 Hz, 1H), 7.25 – 7.38 (m, 4H), 7.58 – 7.69 (m, 2H), 8.46 (d, *J* = 2.1 Hz, 1H); ^{13}C NMR (101 MHz, DMSO-d_6) *δ* 25.1, 29.2, 31.8, 41.7, 98.6, 115.7, 118.9, 123.9, 128.1 (2C), 130.2 (2C), 130.3, 130.4, 131.5, 140.6, 146.7, 149.2, 159.3; FTMS (ESI) m/z: 389.0414 [M+H]+; calc for $C_{19}H_{19}ClBrN_2$: 389.0415.

N-(3-(4-chlorophenyl)propyl)-6-methoxy-2-methylquinolin-4-amine (**8v**):

Column chromatography on silica gel hexane – Ethyl acetate (3:7) followed by recrystallization by n-hexane, off-white solid. Yield: 32 %; mp.: 138-139 ◦C;

UHPLC: 96% (t_R =14.92 min); ^{1}H NMR (400 MHz, Chloroform-*d*) *δ* 2.07 (p, *J* = 7.3 Hz, 2H), 2.56 (s, 3H), 2.76 (t, *J* = 7.5 Hz, 2H), 3.32 (q, *J* = 6.7 Hz, 2H), 3.86 (s, 3H), 5.01 (s, 1H), 6.21 (s, 1H), 6.92 (d, *J* = 2.5 Hz, 1H), 7.13 (d, *J* = 8.1 Hz, 2H), 7.22 – 7.28 (m, 3H), 7.84 (d, *J* = 9.2 Hz, 1H); ^{13}C NMR (101 MHz, Chloroform-*d*) δ 25.2, 30.2, 32.8, 42.7, 55.7, 99.3 (2C), 117.8, 120.2, 128.7 (2C), 129.8 (2C), 130.3, 132.0, 139.7, 143.5, 149.0, 156.4, 156.9; FTMS (ESI) m/z: 341.1415 [M+H]+; calc for $C_{20}H_{22}ClN_2O$: 341.1415.

6-Chloro-2-ethyl-*N*-(3-phenylpropyl)quinolin-4-amine (**8x**):

Column chromatography on silica gel hexane – Ethyl acetate (1:1) followed by recrystallization from hexane, pale yellow solid. Yield: 29%; mp.: 119-120 °C; UHPLC: 95% (t_R =14.95 min); ^{1}H NMR (400 MHz, Chloroform-*d*) *δ* 1.3 (t, *J* = 7.6 Hz, 3H), 2.1 (p, *J* = 7.1 Hz, 2H), 2.8 – 2.9 (m, 4H), 3.3 (q, *J* = 6.5 Hz, 2H), 4.8 (s, 0H), 6.2 (s, 1H), 7.2 – 7.4 (m, 6H), 7.4 (s, 1H), 7.5 (dd, *J* = 9.0, 2.2 Hz, 1H), 7.8 (d, *J* = 9.0 Hz, 1H); ^{13}C NMR (101 MHz, Chloroform-*d*) *δ* 14.1, 30.1, 32.6, 33.7, 42.9, 98.3, 118.3, 118.6, 126.5, 128.4 (2C), 128.8 (2C), 129.4, 129.6, 130.8, 141.2, 146.7, 149.0, 165.0; FTMS (ESI) m/z: 352.1469 [M+H]+; calc. for $C_{20}H_{22}ClN_2$: 352.1466.

6-Bromo-2-ethyl-*N*-(3-phenylpropyl)quinolin-4-amine (**8y**):

Column chromatography on silica gel hexane – Ethyl acetate (1:1) followed by recrystallization from hexane, white solid. Yield: 31 %; mp.: 124–125 °C; UHPLC: 98% (t_R =15.06 min); ^{1}H NMR (400 MHz, Chloroform-*d*) *δ* 1.33 (t, *J* = 7.6 Hz, 3H), 2.11 (p, *J* = 7.1 Hz, 2H), 2.76 – 2.86 (m, 4H), 3.33 (q, *J* = 6.5 Hz, 2H), 4.76 (t, *J* = 5.1 Hz, 1H), 6.25 (s, 1H), 7.20 – 7.37 (m, 5H), 7.56 (d, *J* = 2.1 Hz, 1H), 7.61 (dd, *J*

= 8.9, 2.1 Hz, 1H), 7.76 (d, *J* = 8.9 Hz, 1H); ^{13}C NMR (101 MHz, Chloroform-*d*) *δ* 14.1, 30.1, 32.7, 33.7, 42.9, 98.4, 117.3, 119.0, 121.9, 126.5, 128.4 (2C), 128.8 (2C), 131.1, 132.2, 141.1, 147.0, 148.9, 165.2. FTMS (ESI) m/z: 369.0941 [M+H]+; calc for $C_{20}H_{22}BrN_2$: 369.0961.

6-Chloro-2-ethyl-*N*-(3-(4-isopropylphenyl)propyl)quinolin-4-amine (**8w**):

Column chromatography on silica gel hexane – Ethyl acetate (1:1) followed by recrystallization from hexane, off-white solid. Yield: 24%; mp.: 120–121 °C; UHPLC: 98% (t_R =15.25 min); ^{1}H NMR (400 MHz, Chloroform-*d*) *δ* 1.24 (d, *J* = 6.9 Hz, 6H), 1.33 (t, *J* = 7.6 Hz, 3H), 2.09 (p, *J* = 7.2 Hz, 2H), 2.75 – 2.95 (m, 5H), 3.33 (q, *J* = 6.6 Hz, 2H), 4.78 (s, 1H), 6.26 (s, 1H), 7.12 – 7.22 (m, 4H), 7.45 – 7.54 (m, 2H), 7.84 (d, *J* = 8.8 Hz, 1H); ^{13}C NMR (101 MHz, Chloroform-*d*) *δ* 14.1, 24.0 (2C), 30.2, 32.7, 33.1, 33.7, 42.9, 98.5, 118.4, 118.6, 126.7 (2C), 128.3 (2C), 129.5, 129.6, 130.9, 138.3, 146.7, 147.0, 149.0, 165.0; FTMS (ESI) m/z: 367.1920 [M+H]+; calc for $C_{23}H_{28}ClN_2$: 367.1936.

6- Bromo-2-ethyl-*N*-(3-(4-isopropylphenyl)propyl)quinolin-4-amine (**8z**):

Column chromatography on silica gel hexane – Ethyl acetate (1:1) followed by recrystallization from hexane, pale yellow solid. Yield: 25%; mp.: 121–122 °C; UHPLC: 98% (t_R =15.30 min); ^{1}H NMR (400 MHz, Chloroform-*d*) δ 1.24 (d, *J* = 7.0 Hz, 6H), 1.33 (t, *J* = 7.6 Hz, 3H), 2.09 (p, *J* = 7.1 Hz, 2H), 2.76 – 2.95 (m, 5H), 3.33 (q, *J* = 6.8 Hz, 2H), 4.80 (s, 1H), 6.26 (s, 1H), 7.17 (q, *J* = 7.9 Hz, 4H), 7.62 (dd, *J* = 8.9, 2.1 Hz, 1H), 7.70 (d, *J* = 2.1 Hz, 1H), 7.77 (d, *J* = 8.9 Hz, 1H); ^{13}C NMR (101 MHz, Chloroform-*d*) δ 14.0, 24.0 (2C), 30.2, 32.7, 33.0, 33.7, 42.8, 98.5, 117.3,

119.0, 121.9, 126.7 (2C), 128.3 (2C), 131.1, 132.1, 138.3, 146.9, 147.0, 148.9, 165.2; FTMS (ESI) m/z: 411.1415 [M+H]+; calc for $C_{23}H_{28}BrN_2$: 411.1430.

6- Bromo-2-ethyl-*N*-(3-(4-isopropylphenyl)propyl)quinolin-4-amine (**10a**):

Column chromatography on silica gel hexane – Ethyl acetate (7:3) to hexane – Ethyl acetate (1:1), off-white solid. Yield: 21%; mp.: 96 – 98 °C; UHPLC:98% (t_R = 16.17 min); ^{1}H NMR (400 MHz, Chloroform-*d*) *δ* 1.24 (d, *J* = 6.9 Hz, 6H), 2.11 (q, *J* = 7.2 Hz, 2H), 2.77 (t, *J* = 7.3 Hz, 2H), 2.91 (p, *J* = 6.9 Hz, 1H), 3.34 (t, *J* = 6.3 Hz, 2H), 4.93 (d, *J* = 5.5 Hz, 1H), 6.36 (d, *J* = 5.4 Hz, 1H), 7.17 (q, *J* = 8.0 Hz, 4H), 7.66 (dd, *J* = 8.9, 2.1 Hz, 1H), 7.76 (d, *J* = 2.1 Hz, 1H), 7.83 (d, *J* = 8.9 Hz, 1H), 8.51 (d, *J* = 5.4 Hz, 1H); ^{13}C NMR (101 MHz, Chloroform-*d*) *δ* 24.0 (2C), 30.1, 33.0, 33.7, 42.9, 99.4, 118.2, 120.1, 122.1, 126.7 (2C), 128.3 (2C), 131.7, 132.3, 138.2, 147.0, 147.1, 148.8, 151.3;; FTMS (ESI) m/z: 383.1131 [M+H]+; calc for $C_{21}H_{24}BrN_2$: 383.1117.

6-Chloro-*N*-(3-(4-(dimethylamino)phenyl)propyl)-2-ethylquinolin-4-amine (**10b**):

Column chromatography on silica gel hexane – Ethyl acetate (1:1), pale yellow solid. Yield: 29%; mp.: 56 – 58 °C; UHPLC: 97% (t_R = 14.23 min); ^{1}H NMR (400 MHz, Chloroform-*d*) *δ* 1.33 (t, *J* = 7.6 Hz, 3H), 2.07 (p, *J* = 7.0 Hz, 2H), 2.72 (t, *J* = 7.0 Hz, 2H), 2.82 (q, *J* = 7.6 Hz, 2H), 2.93 (s, 6H), 3.28 – 3.37 (m, 2H), 4.82 (t, *J* = 5.3 Hz, 1H), 6.25 (s, 1H), 6.67 – 6.75 (m, 2H), 7.10 (d, *J* = 8.3 Hz, 2H), 7.39 (d, *J* = 2.2 Hz, 1H), 7.48 (dd, *J* = 9.0, 2.3 Hz, 1H), 7.83 (d, *J* = 8.9 Hz, 1H); ^{13}C NMR (101 MHz, Chloroform-*d*) δ 14.1, 30.1, 32.6, 32.7, 40.7 (2C), 43.1, 98.3, 113.0

(2C), 118.4, 118.7, 128.8, 129.0 (2C), 129.4, 129.6, 130.7, 146.6, 149.2, 149.4, 165.0; FTMS (ESI) m/z: 368.1898 [M+H]+; calc for $C_{22}H_{27}ClN_3$: 368.1888.

6-Bromo-*N*-(3-(4-(dimethylamino)phenyl)propyl)-2-ethylquinolin-4-amine (**10c**):

Column chromatography on silica gel hexane – Ethyl acetate (1:1), pale yellow solid. Yield: 24 %; mp.: 61 – 63 °C; UHPLC: 98% (t_R = 14.56 min.); ^{1}H NMR (400 MHz, Chloroform-*d*) δ 1.33 (t, *J* = 7.6 Hz, 3H), 2.08 (q, *J* = 7.0 Hz, 2H), 2.72 (t, *J* = 7.1 Hz, 2H), 2.81 (q, *J* = 7.6 Hz, 2H), 2.93 (s, 6H), 3.32 (q, *J* = 6.2 Hz, 2H), 4.89 (s, 1H), 6.24 (s, 1H), 6.68 – 6.75 (m, 2H), 7.07 – 7.14 (m, 2H), 7.61 (td, *J* = 5.1, 4.7, 2.1 Hz, 2H), 7.74 – 7.81 (m, 1H); ^{13}C NMR (101 MHz, Chloroform-*d*) δ 14.1, 30.2, 32.5, 32.6, 40.8 (2C), 43.0, 98.3, 113.0 (2C), 117.3, 118.9, 122.0, 128.8, 129.0 (2C), 130.8, 132.2, 146.7, 149.1, 149.4, 165.0; FTMS (ESI) m/z: 412.1390 [M+H]+; calc for $C_{22}H_{27}BrN_3$: 412.1383.

N-(3-((6-chloro-2-methylquinolin-4-yl)oxy)propyl)-4-isopropylaniline (**11a**):

Column chromatography on silica gel hexane – Ethyl acetate (9:1) to hexane – Ethyl acetate (7:3), yellow solid. Yield: 34%; mp.:160-163 °C; UHPLC: 98% (t_R = 20.19 min); ^{1}H NMR (400 MHz, Chloroform-*d*) *δ* 1.18 (d, *J* = 6.9 Hz, 6H), 2.38 (p, *J* = 6.3 Hz, 2H), 2.78 (h, *J* = 6.9 Hz, 1H), 3.00 (s, 3H), 3.46 (t, *J* = 6.4 Hz, 2H), 4.68 (t, *J* = 6.2 Hz, 2H), 6.72 – 6.79 (m, 2H), 7.02 – 7.08 (m, 2H), 7.18 (s, 1H), 7.68 – 7.77 (m, 2H), 8.14 (d, *J* = 2.3 Hz, 1H), 8.60 (d, *J* = 9.1 Hz, 1H); ^{13}C NMR (101 MHz, Chloroform-*d*) *δ* 21.30, 24.18 (2C), 28.07, 33.19, 41.38, 69.61, 104.07, 114.07 (2C), 120.35, 121.85, 122.62, 127.31 (2C), 134.32, 134.43, 138.11, 139.70,

144.40, 159.02, 165.92 ; FTMS (ESI) m/z: 368.1670 [M+H]+; calc for $C_{22}H_{26}ClN_2O$: 368.1655.

N-(3-((6-bromo-2-methylquinolin-4-yl)oxy)propyl)-4-isopropylaniline (**11b**):

Recrystallization by ethyl acetate, translucent red solid. Yield: 22%; mp.:132-133 °C; UHPLC: 97% (t_R = 21.18 min); ^{1}H NMR (400 MHz, Chloroform-*d*) *δ* 1.20 (d, *J* = 6.9, 1.2 Hz, 6H), 2.23 (p, *J* = 6.0 Hz, 2H), 2.64 (s, 2H), 2.80 (hept, *J* = 6.9 Hz, 1H), 3.42 (t, *J* = 6.6 Hz, 2H), 4.26 (t, *J* = 5.9 Hz, 2H), 6.56 – 6.66 (m, 3H), 7.03 – 7.09 (m, 2H), 7.67 – 7.74 (m, 1H), 7.80 (d, *J* = 8.9 Hz, 1H), 8.27 (q, *J* = 2.1 Hz, 1H); ^{13}C NMR (101 MHz, Chloroform-*d*) *δ* 24.24 (2C), 25.92, 28.93, 33.17, 41.11, 66.46, 101.83, 112.89 (2C), 118.62, 121.06, 124.05, 127.24 (2C), 130.01, 133.12, 138.25, 145.99, 147.44, 160.48, 160.65; FTMS (ESI) m/z: 412.1157 [M+H]+; calc for $C_{22}H_{26}BrN_2O$: 412.1150.

N-(3-((6-chloro-2-ethylquinolin-4-yl)oxy)propyl)-4-isopropylaniline (**11c**):

Column chromatography on silica gel hexane – Ethyl acetate (9:1) to hexane – Ethyl acetate (7:3), brown solid. Yield: 34%; mp.: 94-97 °C; UHPLC: 95% (t_R = 21.01 min); ^{1}H NMR (400 MHz, Chloroform-*d*) *δ* 1.20 (d, *J* = 6.9 Hz, 6H), 1.36 (t, *J* = 7.6 Hz, 3H), 2.24 (p, *J* = 6.3 Hz, 2H), 2.80 (hept, *J* = 6.8 Hz, 1H), 2.90 (q, *J* = 7.6 Hz, 2H), 3.43 (t, *J* = 6.6 Hz, 2H), 4.29 (t, *J* = 5.9 Hz, 2H), 6.58 – 6.65 (m, 3H), 7.03 – 7.09 (m, 2H), 7.58 (dd, *J* = 9.0, 2.4 Hz, 1H), 7.89 (d, *J* = 9.0 Hz, 1H), 8.11 (d, *J* = 2.4 Hz, 1H); ^{13}C NMR (101 MHz, Chloroform-*d*) *δ* 13.9, 24.2 (2C), 28.9, 32.8, 33.2, 41.1, 66.4, 100.7, 112.9 (2C), 120.6, 127.2 (2C), 130.0, 130.5, 130.6, 138.2, 146.0, 147.2, 160.8, 165.6; FTMS (ESI) m/z: 382.1804 [M+H]+; calc for $C_{23}H_{28}ClN_2O$: 382.1812.

N-(3-((6-bromo-2-ethylquinolin-4-yl)oxy)propyl)-4-isopropylaniline (**11d**):

Column chromatography on silica gel hexane – Ethyl acetate (9:1) to hexane – Ethyl acetate (7:3), dark brown solid. Yield: 14%; mp.: 89-91 °C; UHPLC: 95% (t_R =18.92 min); ^{1}H NMR (400 MHz, Chloroform-*d*) δ 1.21 (d, J = 6.9 Hz, 6H), 1.36 (t, J = 7.6 Hz, 3H), 2.25 (p, J = 6.3 Hz, 2H), 2.81 (hept, J = 6.9 Hz, 1H), 2.91 (q, J = 7.7 Hz, 2H), 3.43 (t, J = 6.6 Hz, 2H), 4.30 (t, J = 5.9 Hz, 2H), 6.58 – 6.66 (m, 3H), 7.02 – 7.11 (m, 2H), 7.72 (dd, J = 8.9, 2.3 Hz, 1H), 7.84 (d, J = 8.9 Hz, 1H), 8.29 (d, J = 2.2 Hz, 1H); ^{13}C NMR (101 MHz, Chloroform-*d*) δ 13.89, 24.24 (2C), 28.94, 32.76, 33.17, 41.14, 66.50, 100.67, 112.91 (2C), 118.69, 121.27, 124.06, 127.24 (2C), 130.07, 133.13, 138.28, 145.98, 147.30, 160.77, 165.73; FTMS (ESI) m/z: 426.1314 [M+H]+; calc for $C_{23}H_{28}BrN_2O$: 426.1307.

2. *Drug susceptibility assay*

2.1 *Inhibition assay for H37Rv strain of Mtb*

The determination of minimum inhibitory concentrations (MICs) for each synthesized compound was carried out in 96-well microplates. Isoniazid and rifampin served as positive controls, and compound solutions were prepared at concentrations of 2 mg/mL in DMSO. These solutions were then diluted in Middlebrook 7H9 medium containing 10% ADC (albumin, dextrose, and catalase) to achieve a concentration of 20 µg/mL for each compound, containing 2% DMSO (Sigma Aldrich). The presence of crystals was assessed, and if present, the solution was further diluted to half the previous concentration. Only molecules capable of forming a true solution were evaluated. Serial two-fold dilutions of each drug in 100 µL of Middlebrook 7H9 medium containing 10% ADC (BD co.) were prepared directly in 96-well plates, starting with the maximum concentration

allowed by the solubility of each compound. Growth controls without antibiotic and sterility controls without inoculation were included. Mycobacterial strains were cultivated in Middlebrook 7H9 containing 10% OADC (oleic acid, albumin, dextrose, and catalase), 0.05% Tween 80, and 0.2% glycerol. Cells were vortexed with sterile glass beads (4 mm) for 5 min to disrupt clumps and then allowed to settle for 20 min. The supernatants were measured spectrophotometrically at an absorbance of 600 nm. Mtb suspensions were aliquoted and stored at -20 °C. Each suspension was appropriately diluted in Middlebrook 7H9 broth containing 10% ADC to achieve an optical density of 0.6 at 600 nm, and 100 μL was added to each well of the plate except for the sterility controls. The plates were covered, sealed, and incubated at 37 °C. After 7 days of incubation, 60 μL of 0.01% resazurin solution was added to each well, and the plate was incubated for an additional 48 h at 37 °C. The MIC assay is based on a color change from blue to pink, indicating bacterial growth. The MIC was established as the lowest compound concentration before the color change. Three independent tests were performed for each chemical structure, and the MIC values reported represent the highest value among the three assays.[4]

2.2 *Inhibition assay for MDR clinical isolates of M. tuberculosis*

The selected aminoalkylquinolines were further tested by the REMA assay for their inhibitory potential against three MDR clinical isolates of *M. tuberculosis*. The clinical isolates (named PT2, PT12, and PT20) were obtained from patients in the Lisbon Health Region, Lisbon, Portugal.[5] INH and RIF were used as control drugs to demonstrate the MDR phenotype of these isolates. This assay was performed following the protocol described in the inhibition assay for H37Rv strain.

2.3 Determination of minimum inhibitory concentration (ATCC 25618)

The *M. tuberculosis* H37Rv-LP (ATCC 25618) constitutively expressing codon-optimized DsRed (DREAM8)[6] was cultured at 37 °C in Middlebrook 7H9 medium (Difco™) supplemented with 10% v/v oleic acid, albumin, dextrose, and catalase (OADC) (BD), and 0.05% w/v Tween 80 (7H9-Tw-OADC). Assay plates were prepared using a Multidrop Combi (model: 5840300) for medium addition and a VIAFLO Assist Plus (Integra) for adding controls and compounds. The compounds were tested in 10-point 2-fold serial dilutions starting at 100 µM. The final DMSO concentration in the assay was 1%. Growth was measured by optical density (OD) at 590 nm and relative fluorescence intensity (RFU) after 5 days of culture. Plates were read using a Synergy H4 plate reader (BioTek). The MIC was defined as the compound concentration required to inhibit 90% of bacillary growth. Q203 was included as positive control of experiment. Bacterial viability was measured in logarithmic phase cells (OD_{590} = 0.4 – 0.8).

2.4 Determination of minimum inhibitory concentration in the mutant LP-0106898-RM1 (QcrB T313I) of *M. tuberculosis*

The *M. tuberculosis* LP-0106898-RM1 (QcrB T313I) strain was cultured at 37 °C in Middlebrook 7H9 medium (Difco™) supplemented with 10% v/v oleic acid, albumin, dextrose, and catalase (OADC) (BD), and 0.05% w/v Tween 80 (7H9-Tw-OADC). H37Rv-LP (ATCC 25618) is the parental strain. Assay plates were prepared using a Multidrop Combi (model: 5840300) for medium addition and a VIAFLO Assist Plus (Integra) for adding controls and compounds. The compounds were tested in 10-point 2-fold serial dilutions starting at 100 µM. The final DMSO concentration in the assay was 1%. Growth was measured by optical density at 590 nm after 5 days of culture. Plates were read using a Synergy H4 microplate

reader (BioTek). The MIC was defined as the compound concentration required to inhibit 90% of bacillary growth. Bacterial viability was measured in logarithmic phase cells (OD_{590} = 0.4 – 0.8).

2.5 *Evaluation of spectrum activity*

The compounds were screened in vitro against reference strains *Staphylococcus aureus* ATCC 25923, *Escherichia coli* ATCC 25922, and *Bacillus cereus* ATCC 33019, as well as extensively drug-resistant clinical isolates of *Acinetobacter baumannii* and *Klebsiella pneumoniae*, and an *Enterococcus faecium* isolate obtained from the hospital environment. Minimum inhibitory concentrations were determined in triplicate using the broth microdilution method for the compounds **8w** and **8z**, following the guidelines of the European Committee on Antimicrobial Susceptibility Testing.[7]

3. *Cytotoxicity investigation*

The evaluation of Vero and HepG2 cell viability was conducted using MTT (3-[4,5-dimethylthiazol-2-yl]-2,5 diphenyl tetrazolium bromide) and neutral red uptake (NRU) assays.[8,9] Both cell lines were grown in DMEM medium (Dulbecco's Modified Eagle Medium, Gibco, Grand Island, NY, USA) supplemented with 10% inactivated fetal bovine serum from Invitrogen, 1% antibiotic (penicillin and streptomycin) from Gibco, and 0.1% fungizone from Gibco. Cells were seeded in a 96-well culture plate (5×10^3 HepG2 and 4×10^3 Vero) and incubated overnight at 37 °C to allow for cell attachment. The media was then removed and replaced with fresh media containing each compound at a final concentration of 0,0655 µM, 0,1638 µM, 0.4096 µM, 1.024 µM, 2.56 µM, 16 µM and 40 µM using 0.5% DMSO, except for the 40 µM dilution was used 1% DMSO due to crystal formation. Cells were incubated during 48 h at 37 °C under a 5% CO_2 atmosphere. After the

incubation, medium was removed and replaced with MTT reagent (5 mg/mL), followed by additional 3 h incubation. Then, 100 μL of DMSO was added to dissolve the formazan crystals and the absorbance was measured at 562 nm (EZ Read 400 microplate reader, Biochrom, Cambridge, UK). Precipitated formazan crystals were used as a direct measure of living cells with active mitochondrial metabolism. For the NRU assay, after 48 h of cell incubation with the treatments, the cells were washed with PBS and 200 μL of neutral red dye solution (40 μg/mL, Sigma) prepared in serum-free medium was added to each well. The plate was then incubated for 2 h at 37 °C under 5% CO_2. Afterward, cells were washed with PBS, and 150 μL of a desorb solution (ethanol/acetic acid/water, 50:1:49) was added to each well and shaken gently for 30 min to extract the neutral red dye from viable cells. The absorbance was measured at 562 nm (EZ Read 400 microplate reader (Biochrom, Holliston, MA, USA)). The percentage of cell viability was calculated using the vehicle control wells (1% DMSO) as the maximum cell viability for 40 μM dilution and using the vehicle control wells (0.5% DMSO) as the maximum cell viability for other dilution. The results were presented as concentration required to reduce cell viability by 50% (CC_{50}) using three independent experiments performed in triplicate.

4. *Solubility assay*

The solubility tests were performed according to a previously published protocol with slight modifications.[10] Accordingly, 1 mL of a prepared solution (0.1 M NH_4HCO_3, pH 9.1; 1 ×PBS, pH 7.4; 0.1 M HCl, pH 1.0) was added to 1 mg of compound (in triplicate). The final solutions were vortexed (1 min), and the resulting suspensions were shaken for 4 h at 25 ◦C. Then, the suspensions were centrifuged (13000 rpm for 20 min at 25 ◦C) obtaining a pellet and the remaining

solutions were quantified by liquid chromatography (as per the conditions already described for UHPLC experiments) using single-point calibration of a known concentration of the compounds in DMSO.

5. Chemical Stability assay

The experiment was carried out by the Center for Applied Mass Spectrometry (CEMSA), São Paulo, Brazil. The test compounds (10 μM) were incubated at 37 °C for 24 h in the presence of pH-controlled buffer solution at pH 1.2 (simulating the pH of the stomach—0.1 M HCl), pH 7.4 (simulating plasma pH—phosphate buffer) and pH 9.1 (simulating intestinal pH—0.1 M NH_4HCO_3). The compounds were quantified by HPLC–MS/MS. The analytical control was Alprenolol. The results were presented as the percentage, comparing the signal at time zero of the assay (100%) with the signal produced by concentrations after the incubation period.

6. Permeability assay

The experiment was carried out by the Center for Applied Mass Spectrometry (CEMSA), São Paulo, Brazil. The Parallel Artificial Membrane Permeability Assay (PAMPA) assay consists of quantifying the test compound (via HPLC–MS/MS) after an incubation period, in two solutions separated by an artificial lipid membrane. The result of this test is expressed in units of diffusion rate (permeation). The procedure comprised the following steps: (1) preparing the membrane, which contains lipids, with specific activating solutions, creating a hydrophobic surface that simulates the intestinal epithelial cell; (2) adding the test compound at a concentration of 10 μM to the donor aqueous phase (buffered pH 7.4); (3) after 5 h at room temperature, an aliquot of the solution receptor (buffered

at pH 7.4) is removed, in which the compound is transported by passive diffusion, for quantification by HPLC–MS/MS.

7. Metabolic Stability

The experiment was carried out by the Center for Applied Mass Spectrometry (CEMSA), São Paulo, Brazil. In brief, the metabolic stability assay was performed in the presence of rat liver microsomes. Compounds were incubated at 37 °C in a buffered solution containing 1 mg/mL of microsomal protein and nicotinamide adenine dinucleotide (NADH). The compound concentration was determined by HPLC–MS/MS at each incubation time (0, 5, 15, and 30 min) and the percentage remaining versus time curve was determined. Verapamil was used as an analytical control.

8. Zebrafish Maintenance

Embryos and larvae (0-5 days post-fertilization (dpf)), *Danio rerio*, from the AB background were obtained from our breeding colony. The animals were maintained in recirculating systems (Zebtec, Tecniplast, Italy) with reverse osmosis filtered water equilibrated to reach the species standard temperature (28 °C ± 2° C), pH (7.0 and 7.5), and ammonia, nitrite, nitrate and chloride levels. Animals were subjected to light/dark cycle of 14/10 hours, respectively (Westerfield, 2000).[11] For breeding, female and males (1:2) were placed in breeding tanks overnight separated by a transparent barrier that was removed after the lights went on the following morning. The fertilized eggs retained in the fitted tank bottom were collected, sanitized and immediately subjected to the treatment. Water used in the experiments was obtained from a reverse osmosis apparatus (18 MOhm/cm) and was reconstituted with marine salt (Crystal Sea™,

Marinemix, Baltimore, USA) at 0.4 ppt. The total organic carbon concentration was 0.33 mg/L. The total alkalinity (as CO_3^{2-}) was 0.030 mEq/L. During fish maintenance, water parameters were monitored daily and maintained in the following ranges: pH: 6.5 to 7.5, conductivity: 400 to 600 μS, ammonium concentration: < 0.004 ppm, and temperature: 25 to 28 °C. All protocols were approved by the Animal Care Committee from Pontifícia Universidade Católica do Rio Grande do Sul (7249, CEUA/PUCRS).

8.1 *Treatment*

Embryos were placed in Petri dishes (30 embryos per dish), for morphological and cardiotoxicity experiments, and exposed to system water (control group), 0.05% DMSO (vehicle), 0.1, 0.3, 1, 3, and 10 μM for five days (from 1-hour post fertilization (hpf) to 5 dpf). Animals were monitored daily for survival rate using an inverted stereomicroscope (Nikon, Melville, EUA).

8.2 *Morphological evaluation.*

Morphological evaluation larvae were daily monitored and registered at 5 dpf under a stereomicroscopy (3x) (n = 30) The body length (μm), ocular distance (μm) and surface area of the eyes (μm^2) was measured after photographical registration using the software NIS-Elements D 3.2 for Windows, supplied by Nikon Instruments Inc. (Melville, USA). Body length was defined as the distance from the larval mouth to the pigmented tip of the tail, the ocular distance was evaluated by the distance between the inner edge of the two eyes (similar to the inner intercantal distance in humans), and the size of the eyes was determined by measuring the surface area of the eyes.[12,13]

8.3 *Cardiotoxicity and cardiac evaluation*

Animals were monitored daily for cardiac alterations, and the heartbeat rate were analyzed at 2 and 5 dpf under a stereomicroscope (n = 20-40). Treated larvae and controls were placed in 96 well plates with their heart rate were monitored for 60 seconds. For all procedures, temperature was kept stable at 28 ºC (Martinelli et al., 2017).[14]

8.4 *Statistical analysis*

Data normality was analyzed by Kolmogorov-Smirnov. Data normally distributed were expressed as mean ± standard deviation (S.D) and further analyzed by one-way ANOVA followed by a post hoc Tukey's test. Nonparametric data were expressed as mean ± standard deviation (S.D) and analyzed by Kruskal-Wallis test followed by a post hoc Dunn's test. The significance level was set at $p < 0.05$. Survival rates throughout the 5 experimental days of exposure were analyzed by a Kaplan-Meier test.

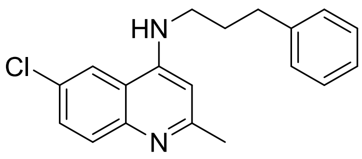

6-Chloro-2-methyl-*N*-(3-phenylpropyl)quinolin-4-amine (**8a**)

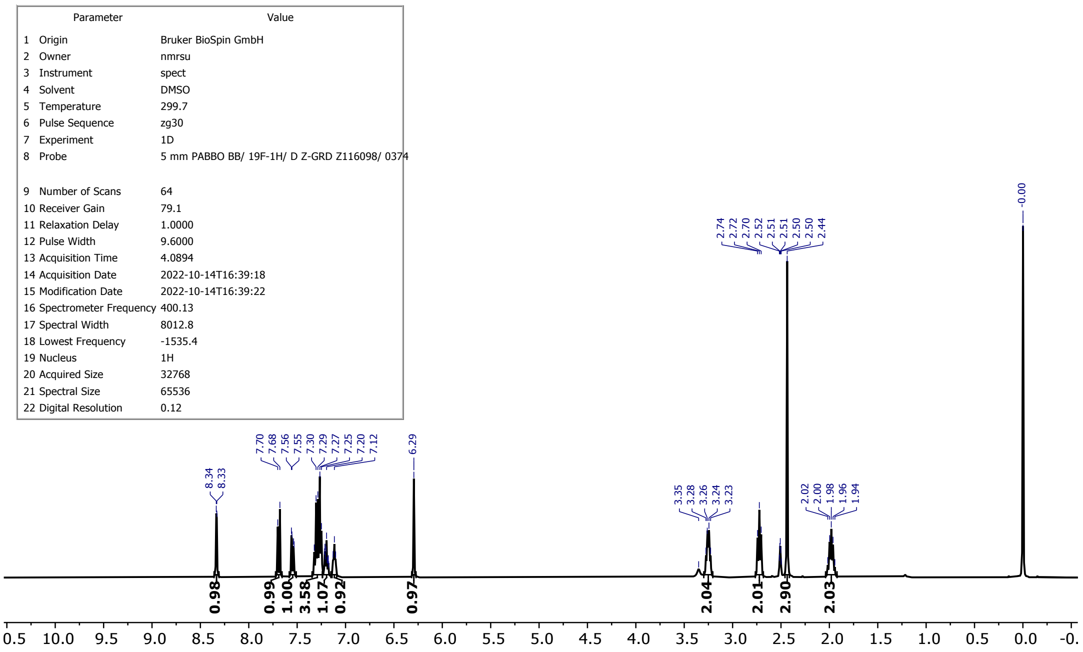

Figure S1. ^{1}H NMR (400 MHz, DMSO-d_6) spectrum of compound **8a**.

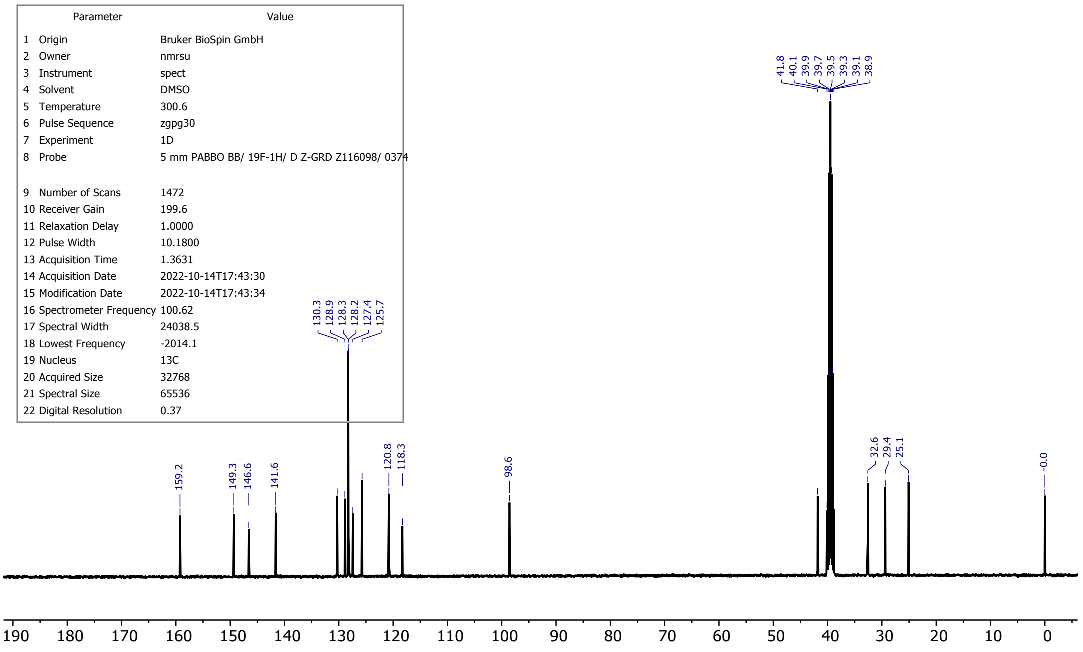

Figure S2. ^{13}C NMR (101 MHz, DMSO-d_6) spectrum of compound **8a**.

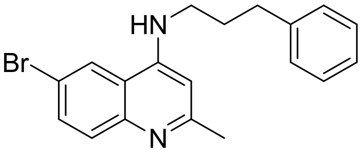

6-Bromo-2-methyl-*N*-(3-phenylpropyl)quinolin-4-amine (**8b**)

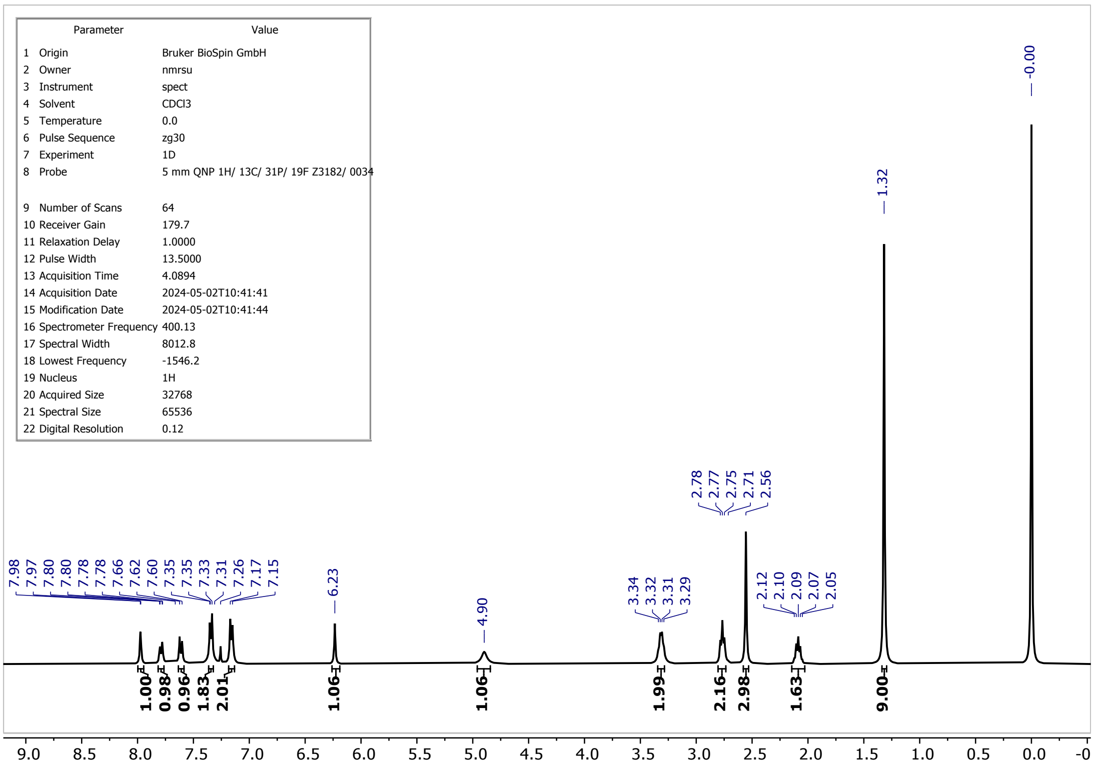

Figure S3. ^{1}H NMR (400 MHz, Chloroform-*d*) spectrum of compound **8b**.

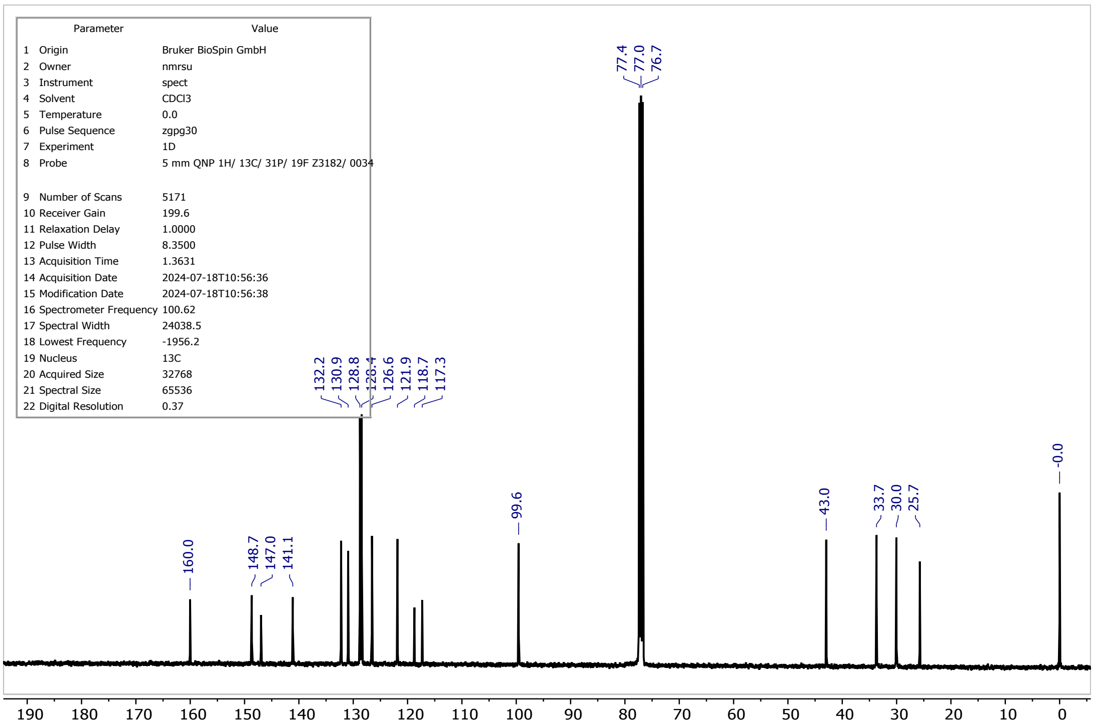

Figure S4. ^{13}C NMR (101 MHz, Chloroform-*d*) spectrum of compound **8b**.

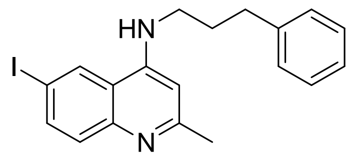

6-Iodo-2-methyl-*N*-(3-phenylpropyl)quinolin-4-amine (**8c**)

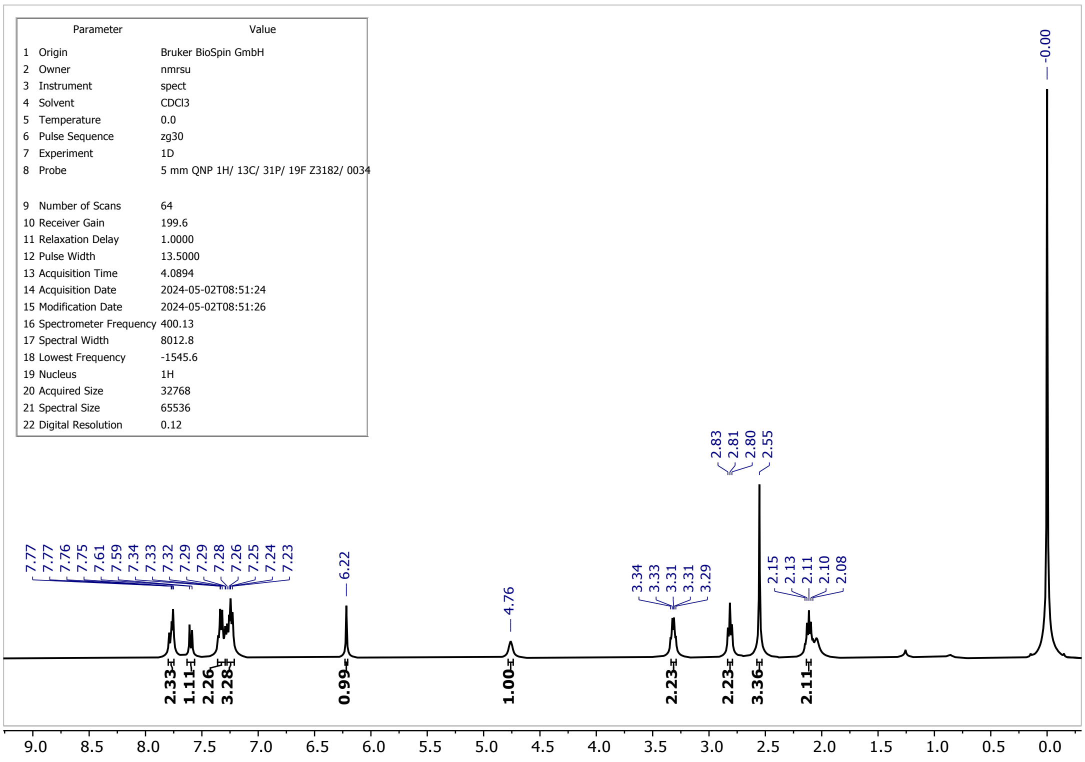

Figure S5. ^{1}H NMR (400 MHz, Chloroform-*d*) spectrum of compound **8c**.

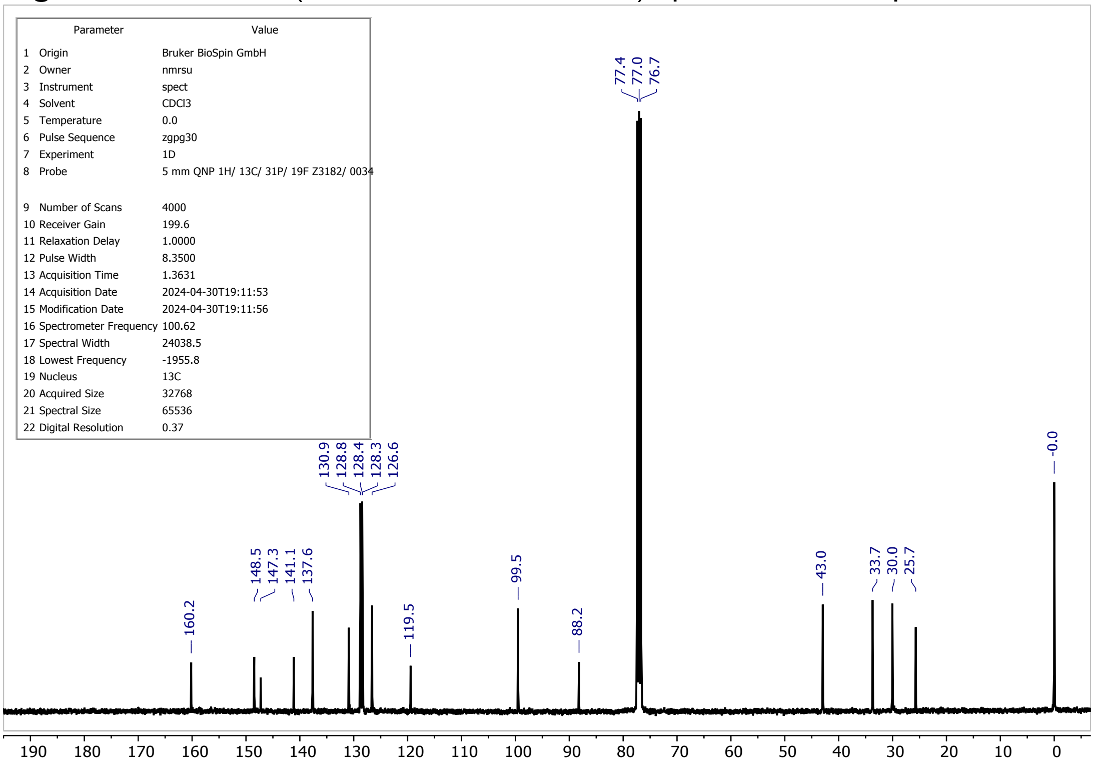

Figure S6. ^{13}C NMR (101 MHz, Chloroform-*d*) spectrum of compound **8c**.

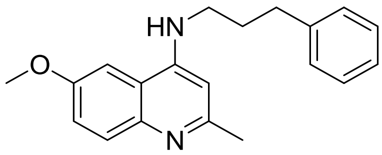

6-Methoxy-2-methyl-*N*-(3-phenylpropyl)quinolin-4-amine (**8d**)

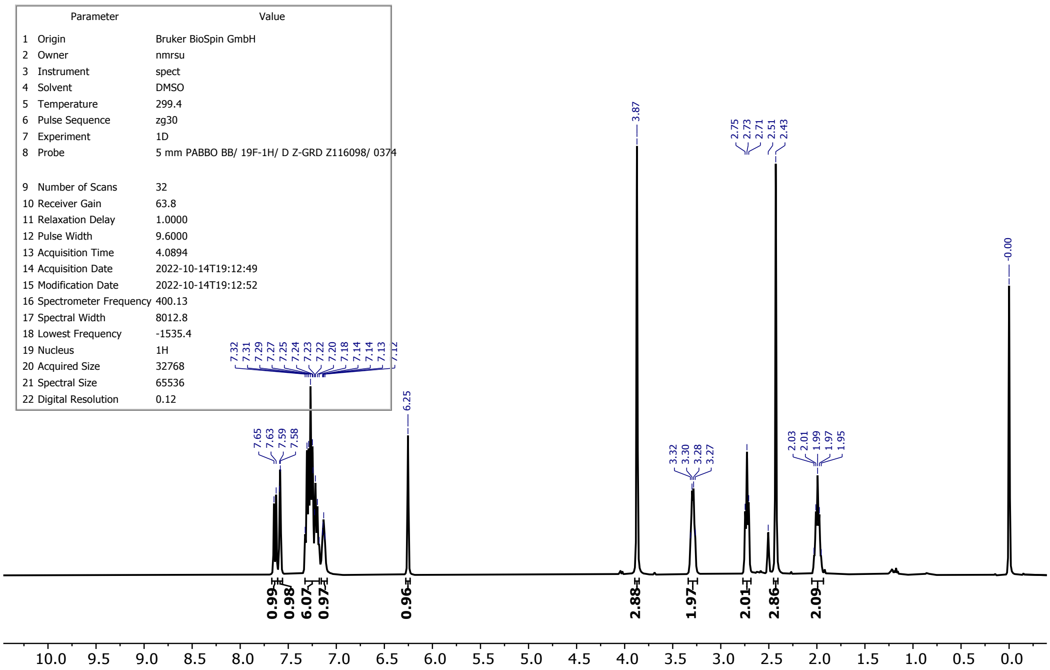

Figure S7. ^{1}H NMR (400 MHz, DMSO-d_6) spectrum of compound **8d**.

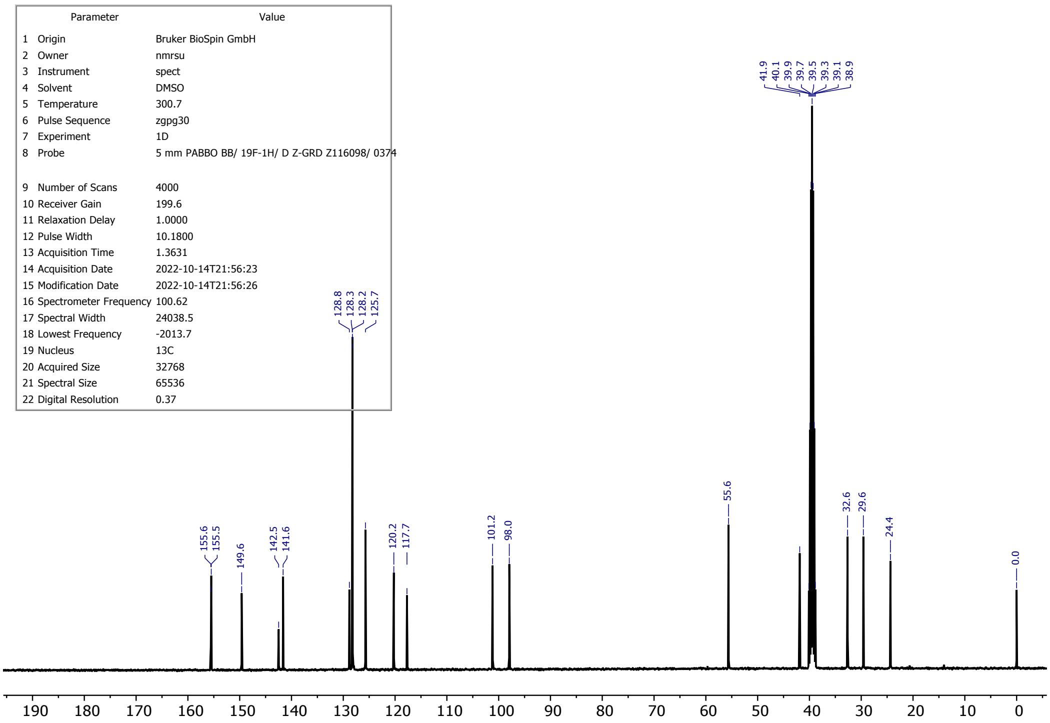

Figure S8. ^{13}C NMR (101 MHz, DMSO-d_6) spectrum of compound **8d**.

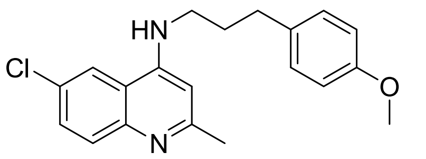

6-Chloro-*N*-(3-(4-methoxyphenyl)propyl)-2-methylquinolin-4-amine (**8e**)

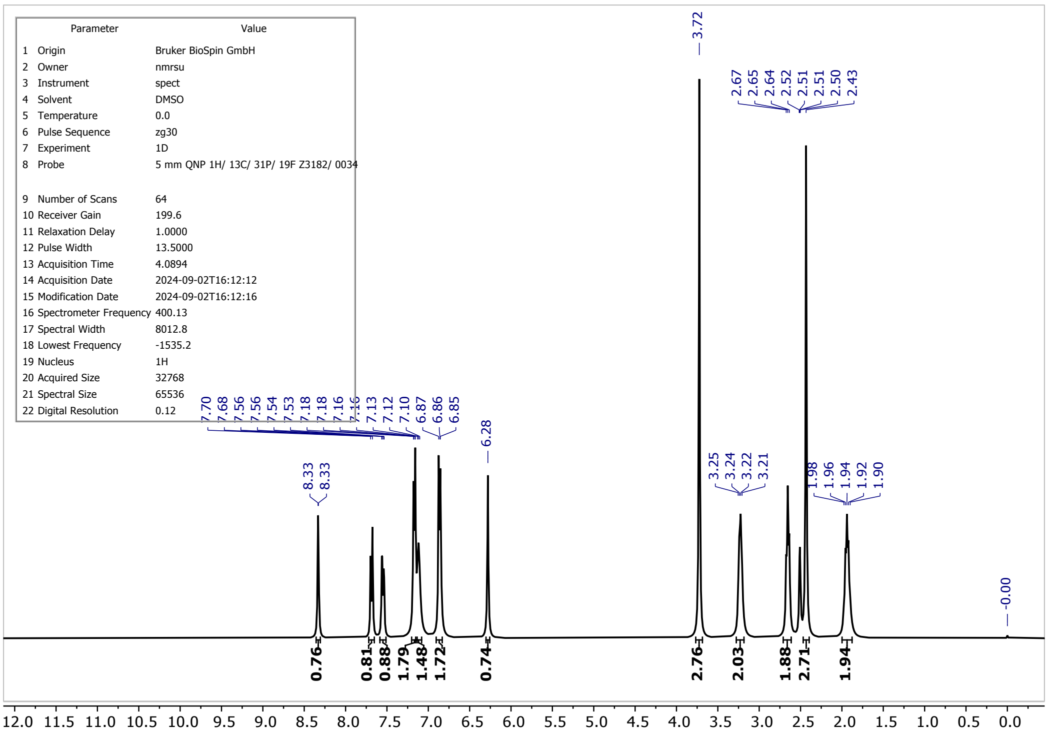

Figure S9. ^{1}H NMR (400 MHz, DMSO-d_6) spectrum of compound **8e**.

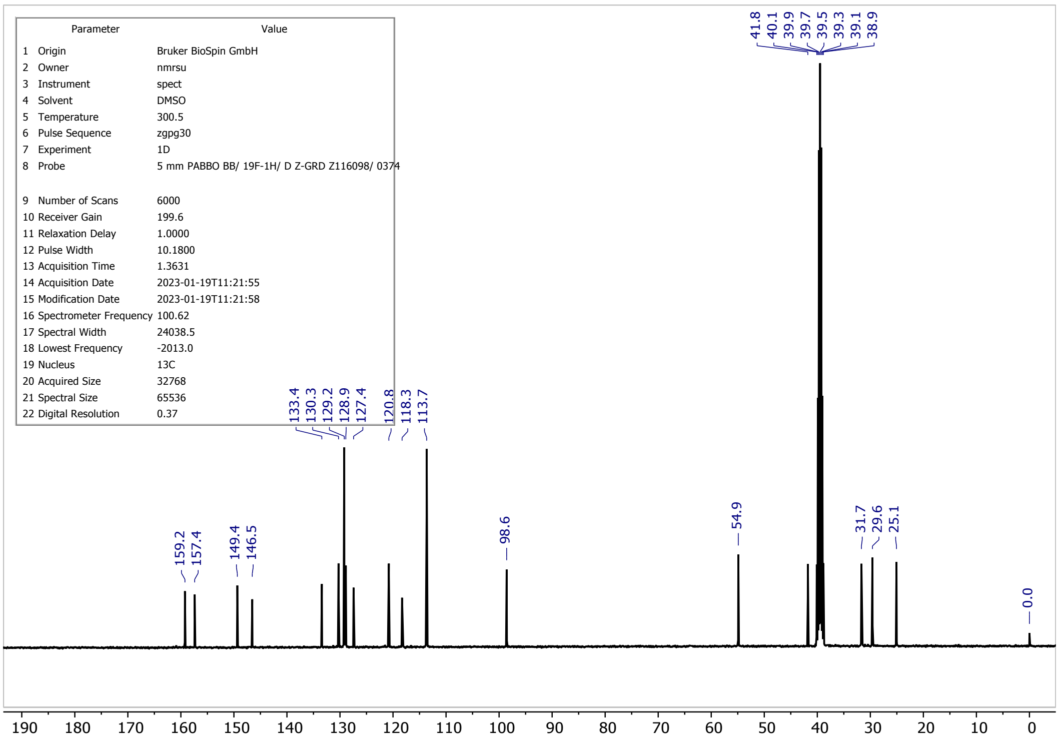

Figure S10. ^{13}C NMR (101 MHz, DMSO-d_6) spectrum of compound **8e**.

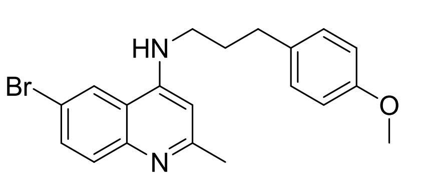

6-Bromo-*N*-(3-(4-methoxyphenyl)propyl)-2-methylquinolin-4-amine (**6f**)

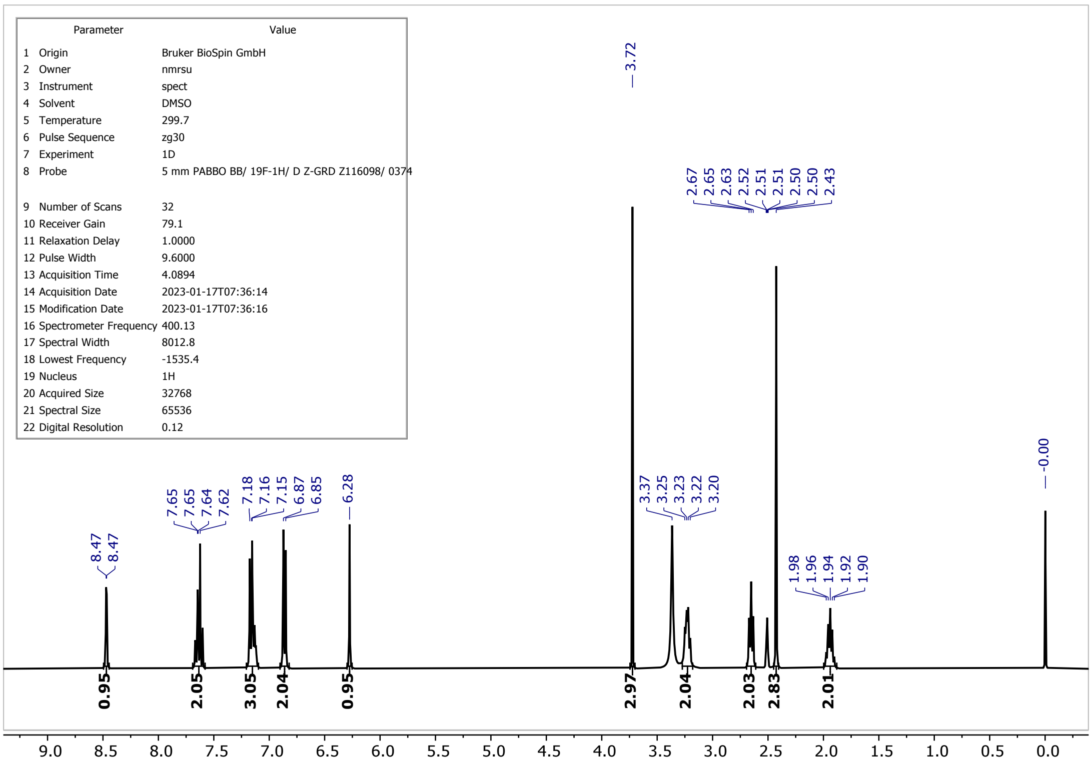

Figure S11. ^{1}H NMR (400 MHz, DMSO-d_6) spectrum of compound **8f**.

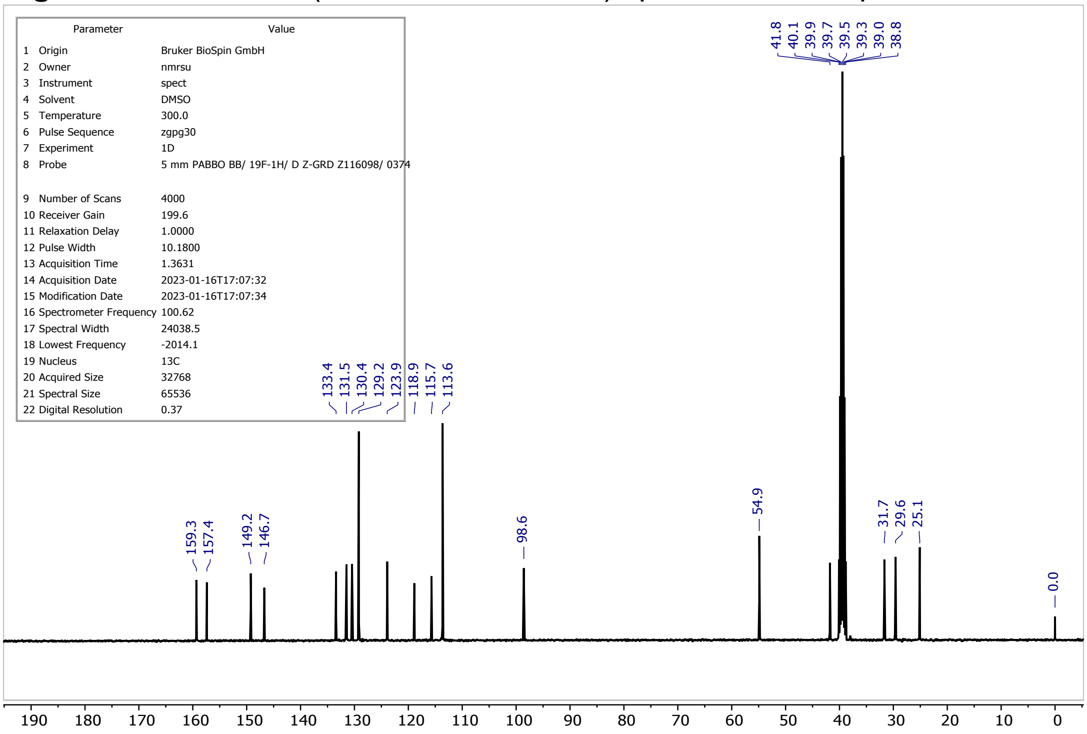

Figure S12. ^{13}C NMR (101 MHz, DMSO-d_6) spectrum of compound **8f**.

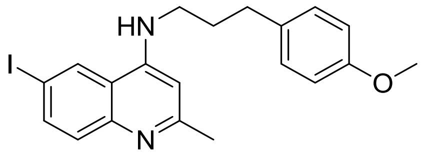

6-Iodo-*N*-(3-(4-methoxyphenyl)propyl)-2-methylquinolin-4-amine (**8g**)

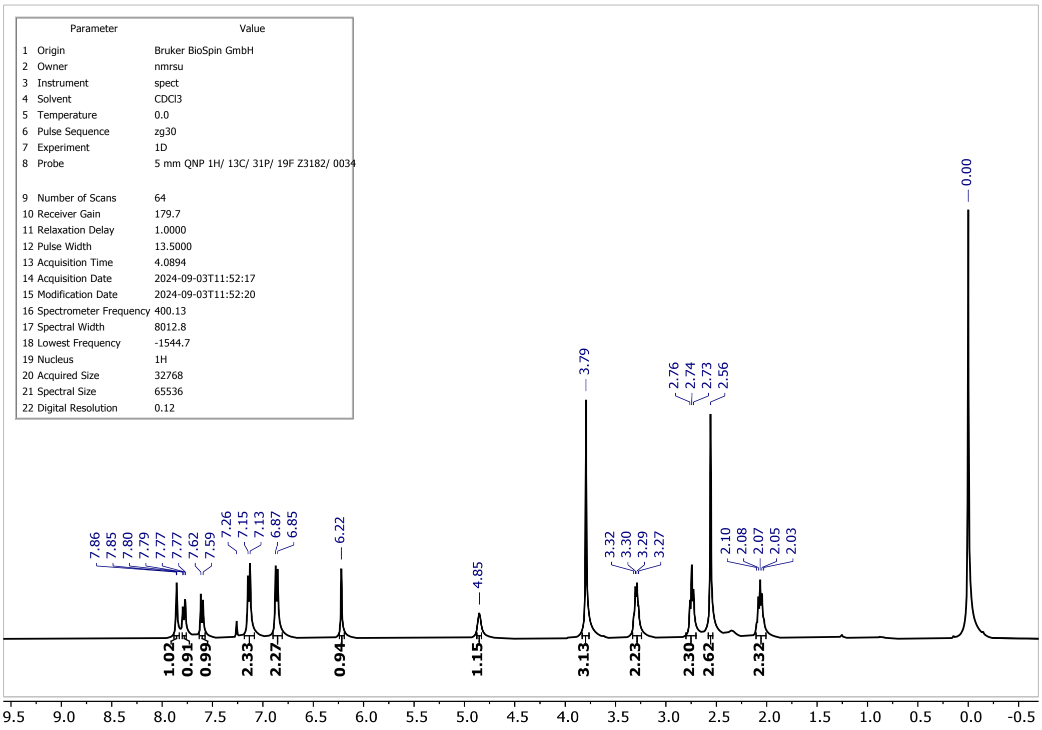

Figure S13. ^{1}H NMR (400 MHz, Chloroform-*d*) spectrum of compound **8g**.

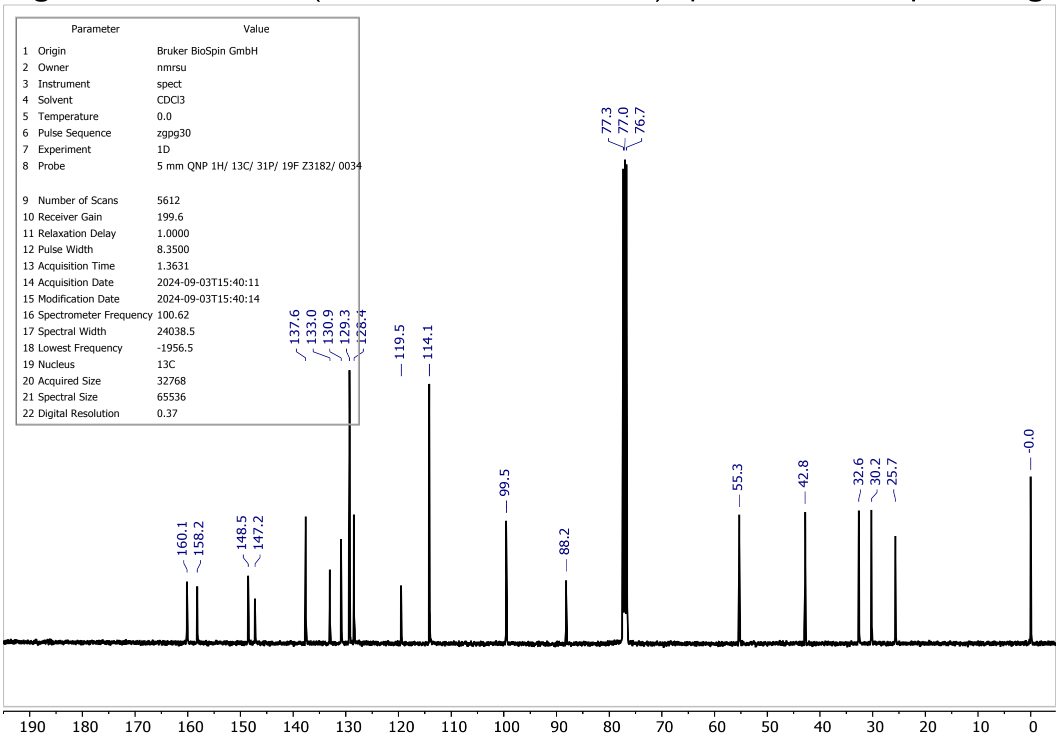

Figure S14. ^{13}C NMR (101 MHz, Chloroform-*d*) spectrum of compound **8g**.

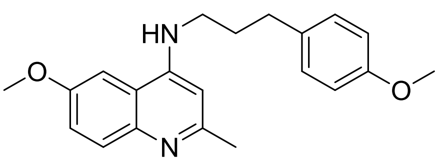

6-Methoxy-*N*-(3-(4-methoxyphenyl)propyl)-2-methylquinolin-4-amine (**8h**)

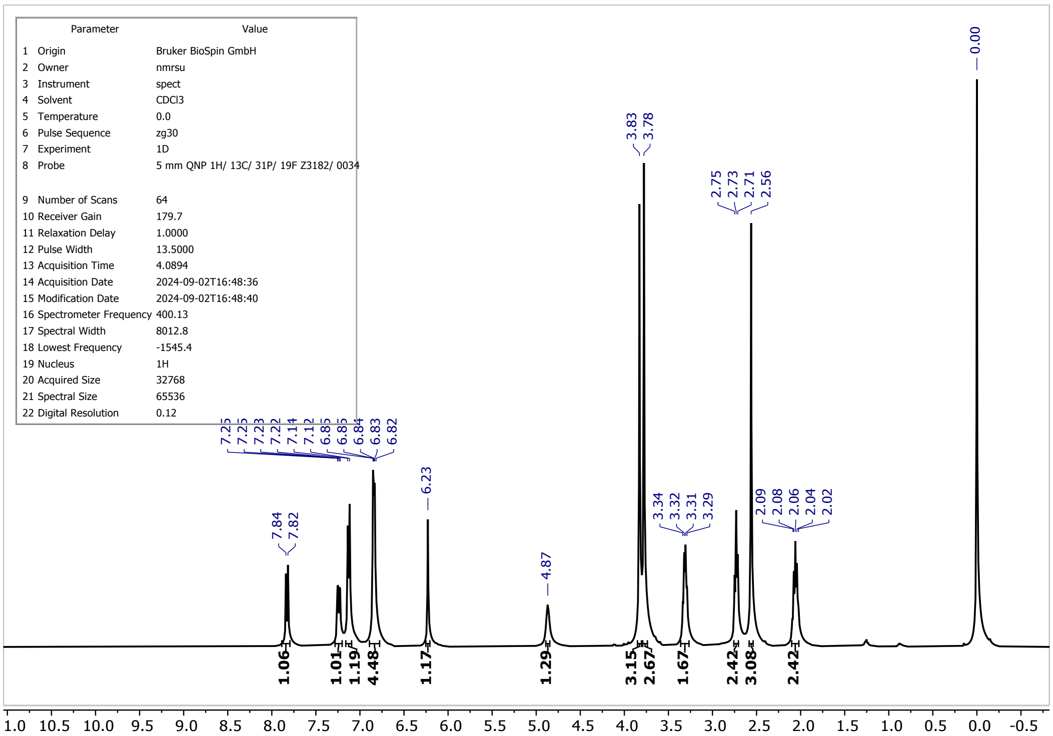

Figure S15. ^{1}H NMR (400 MHz, Chloroform-*d*) spectrum of compound **8h**

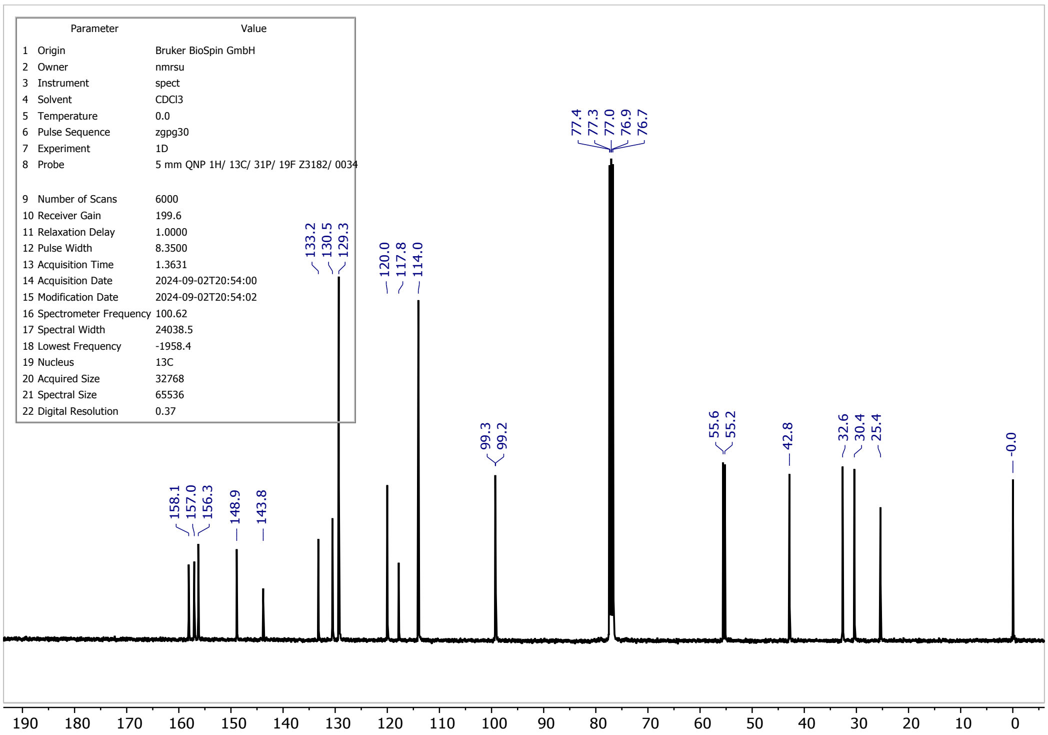

Figure S16. ^{13}C NMR (101 MHz, Chloroform-*d*) spectrum of compound **8h**.

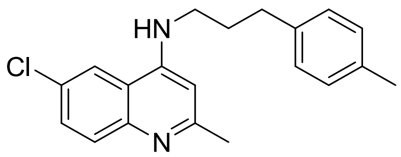

6-Chloro-2-methyl-*N*-(3-(*p*-tolyl)propyl)quinolin-4-amine (**6i**)

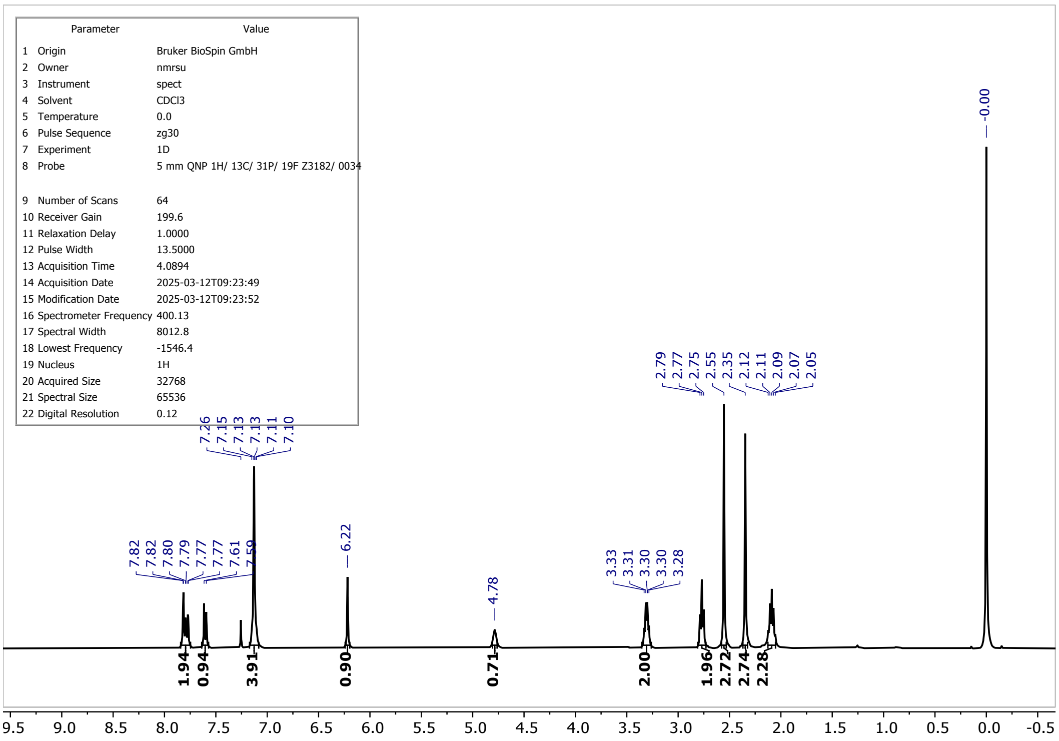

Figure S17. ^{1}H NMR (400 MHz, Chloroform-*d*) spectrum of compound **8i**.

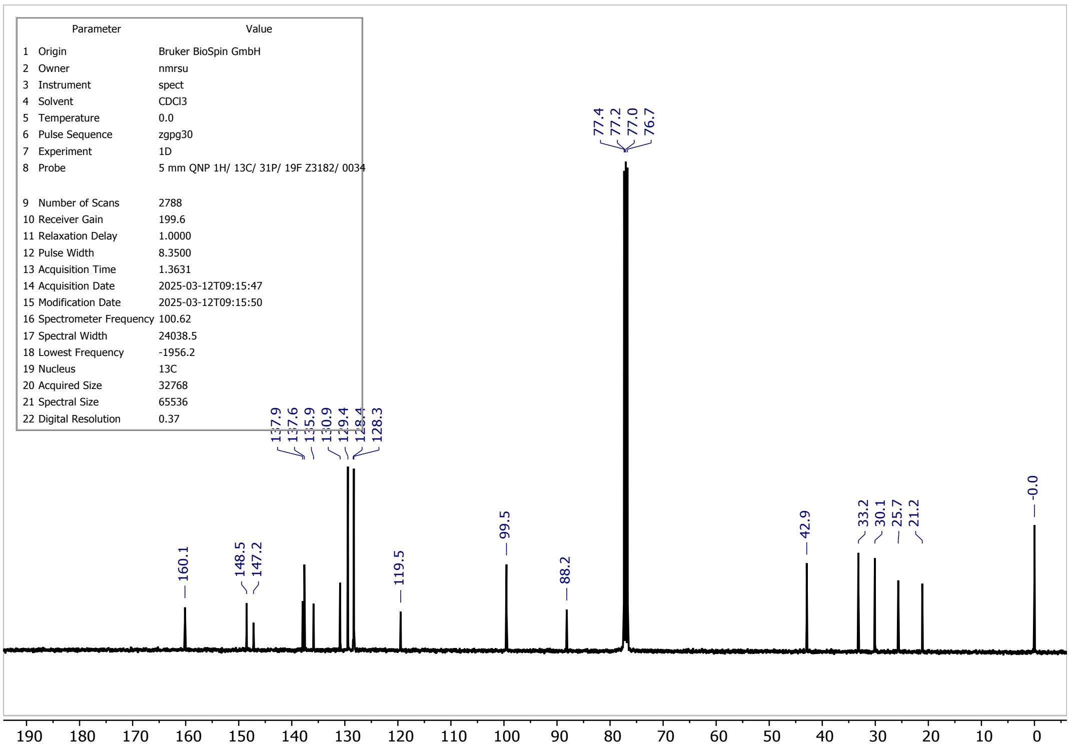

Figure S18. ^{13}C NMR (101 MHz, Chloroform-*d*) spectrum of compound **8i**.

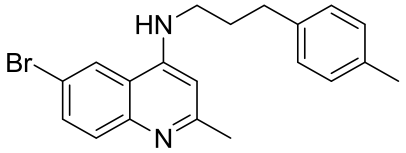

6-Bromo-2-methyl-*N*-(3-(*p*-tolyl)propyl)quinolin-4-amine (**8j**)

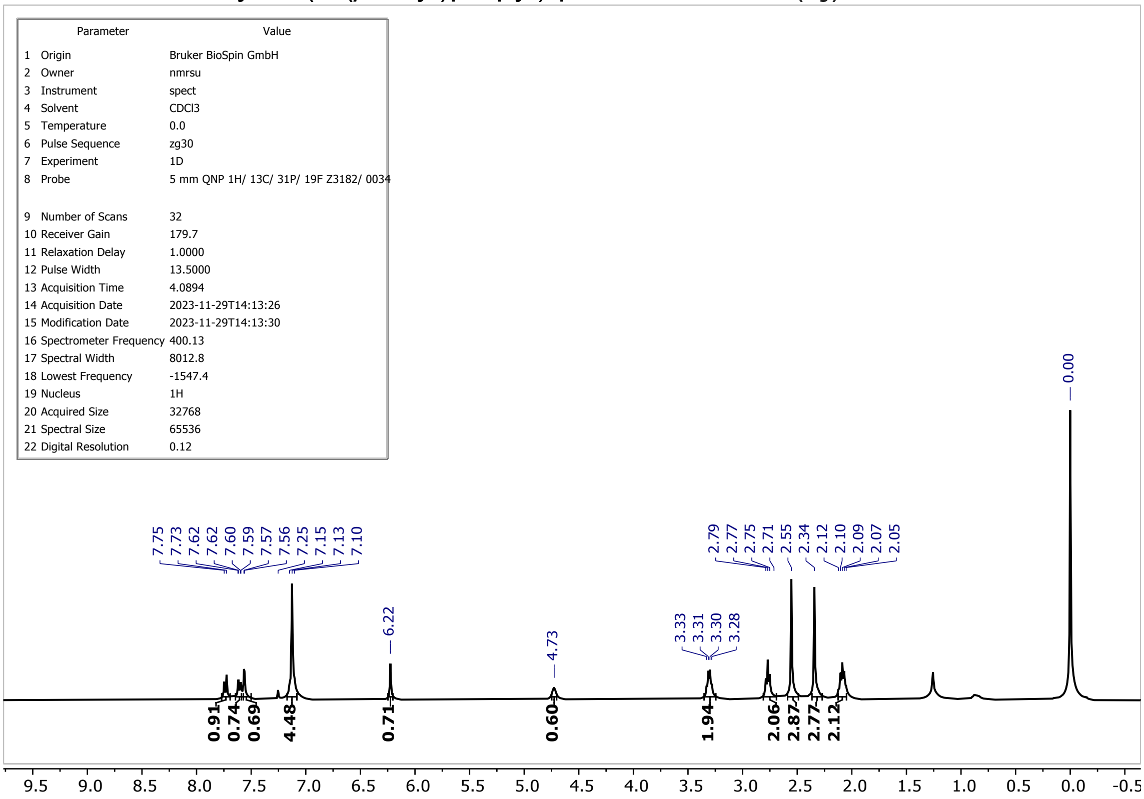

Figure S19. ^{1}H NMR (400 MHz, Chloroform-*d*) spectrum of compound **8j**.

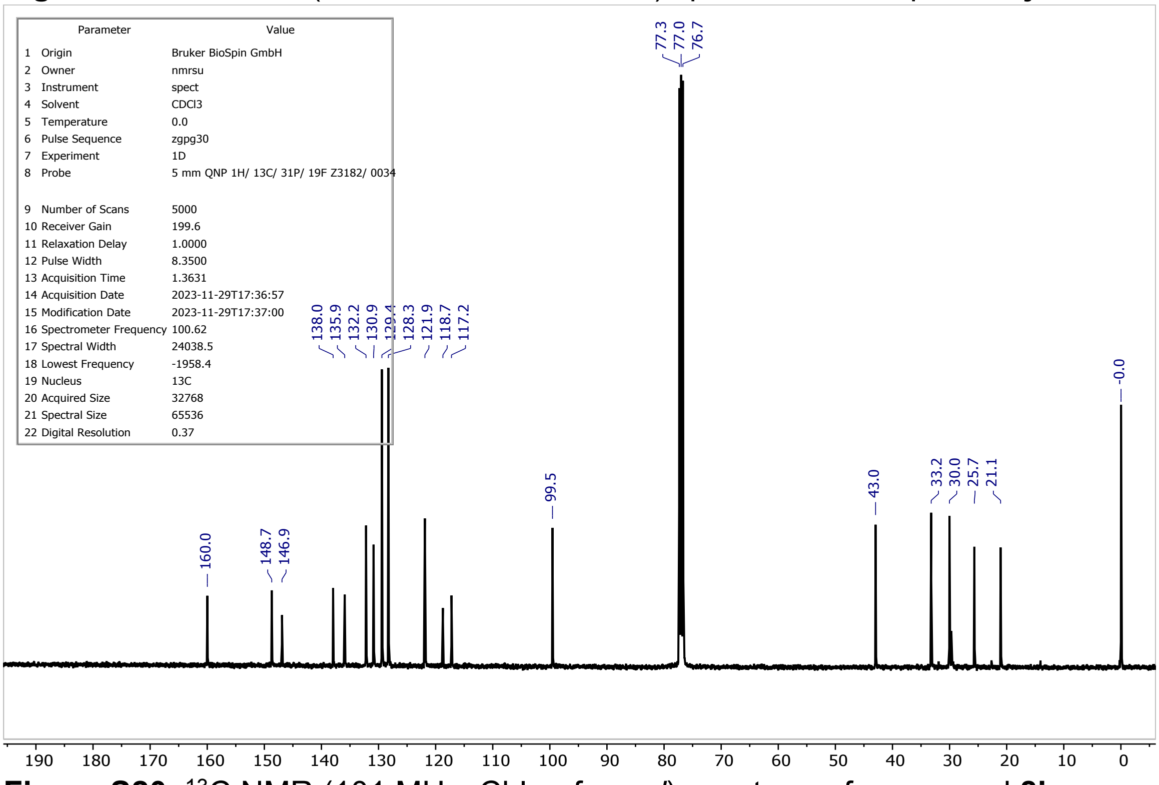

Figure S20. ^{13}C NMR (101 MHz, Chloroform-*d*) spectrum of compound **8j**.

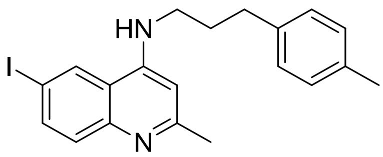

6-Iodo-2-methyl-*N*-(3-(*p*-tolyl)propyl)quinolin-4-amine (**8k**)

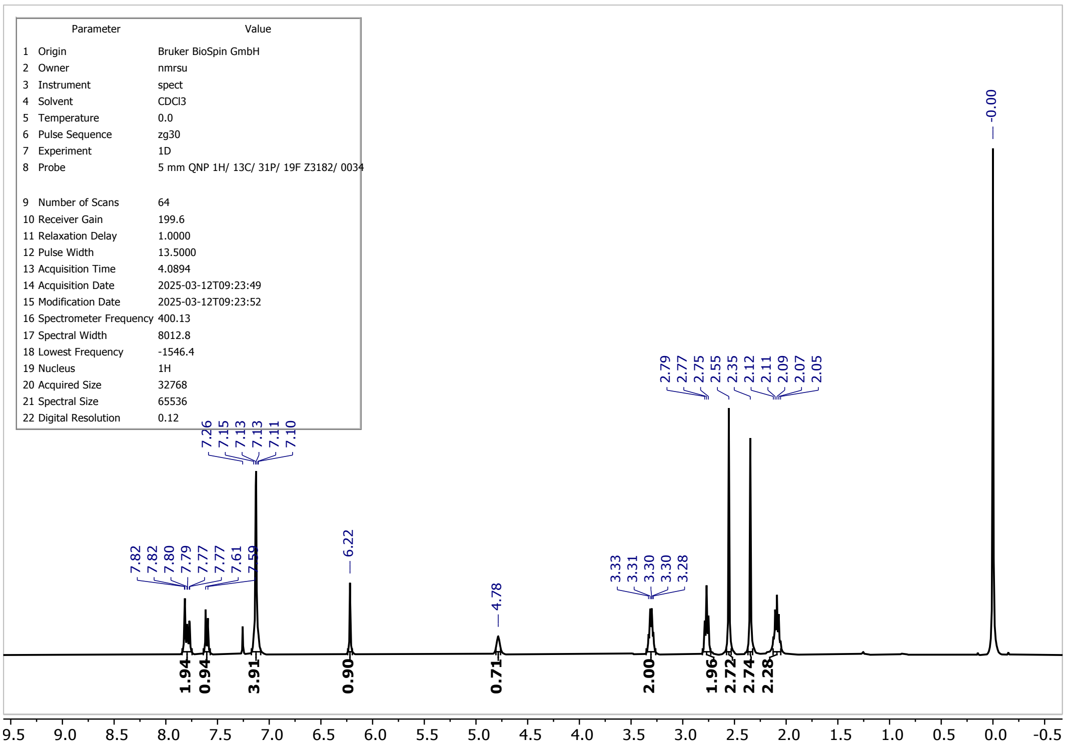

Figure S21. ^{1}H NMR (400 MHz, Chloroform-*d*) spectrum of compound **8k**.

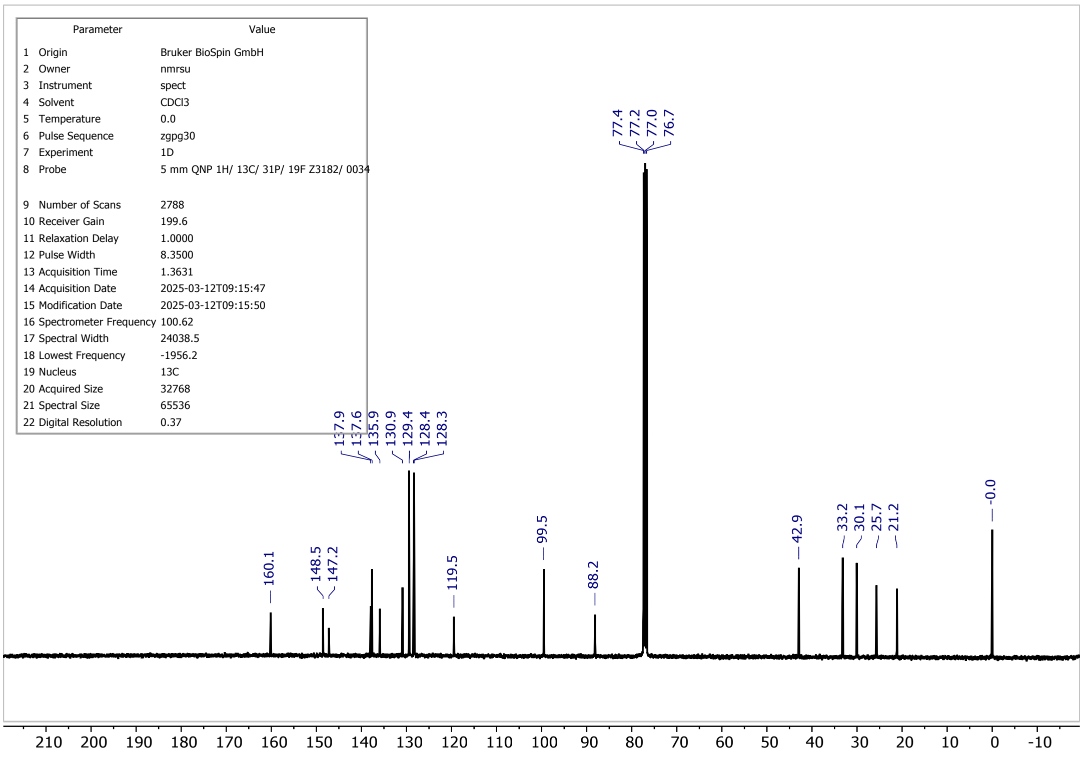

Figure S22. ^{13}C NMR (101 MHz, Chloroform-*d*) spectrum of compound **8k**.

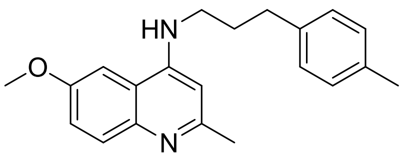

6-Methoxy-2-methyl-*N*-(3-(*p*-tolyl)propyl)quinolin-4-amine (**8l**)

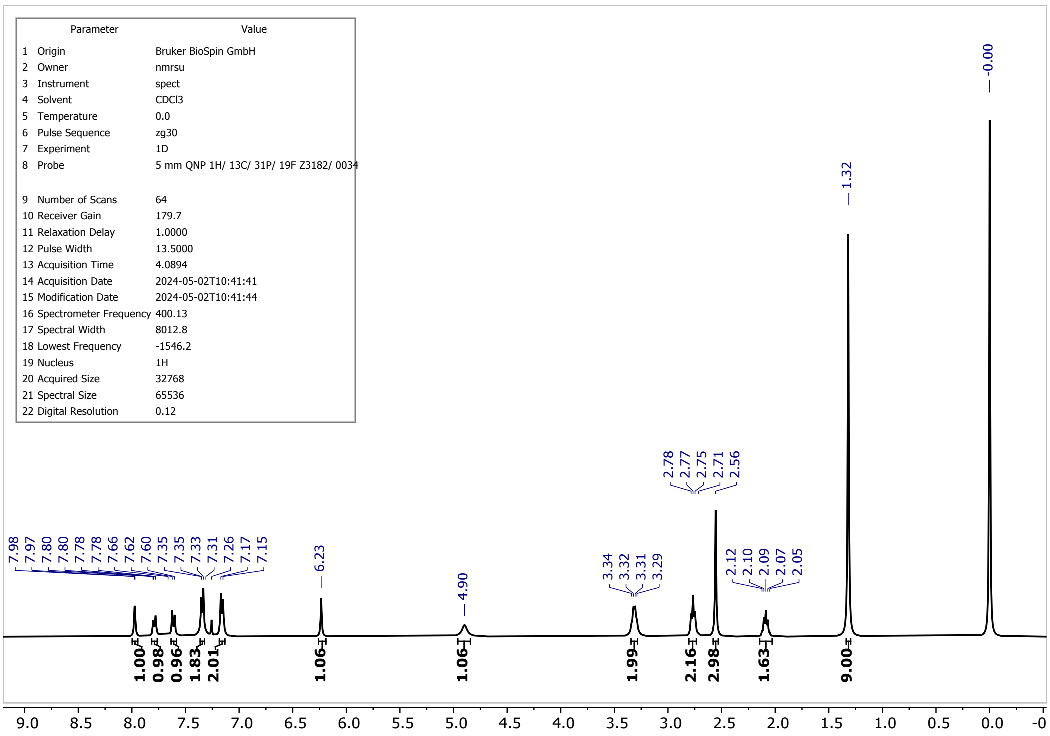

Figure S23. ^{1}H NMR (400 MHz, Chloroform-*d*) spectrum of compound **8l**.

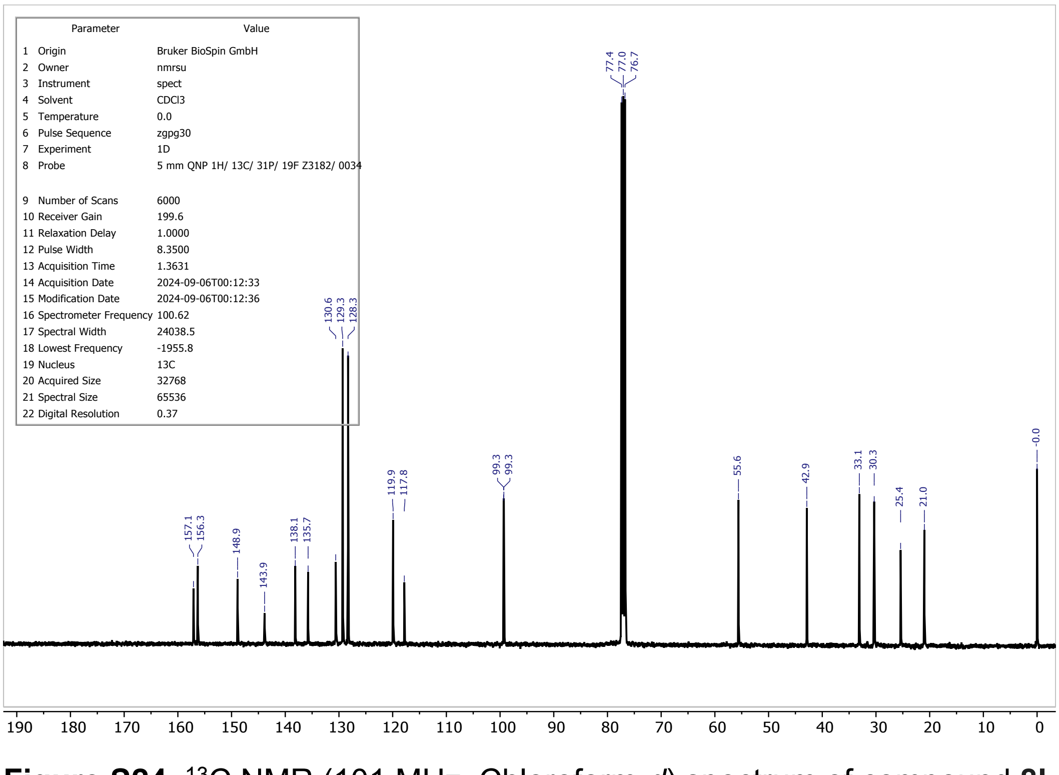

Figure S24. ^{13}C NMR (101 MHz, Chloroform-*d*) spectrum of compound **8l**.

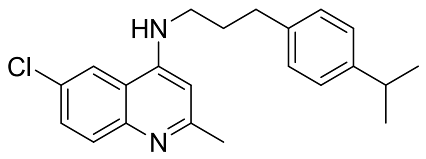

6-Chloro-*N*-(3-(4-isopropylphenyl)propyl)-2-methylquinolin-4-amine (**8m**)

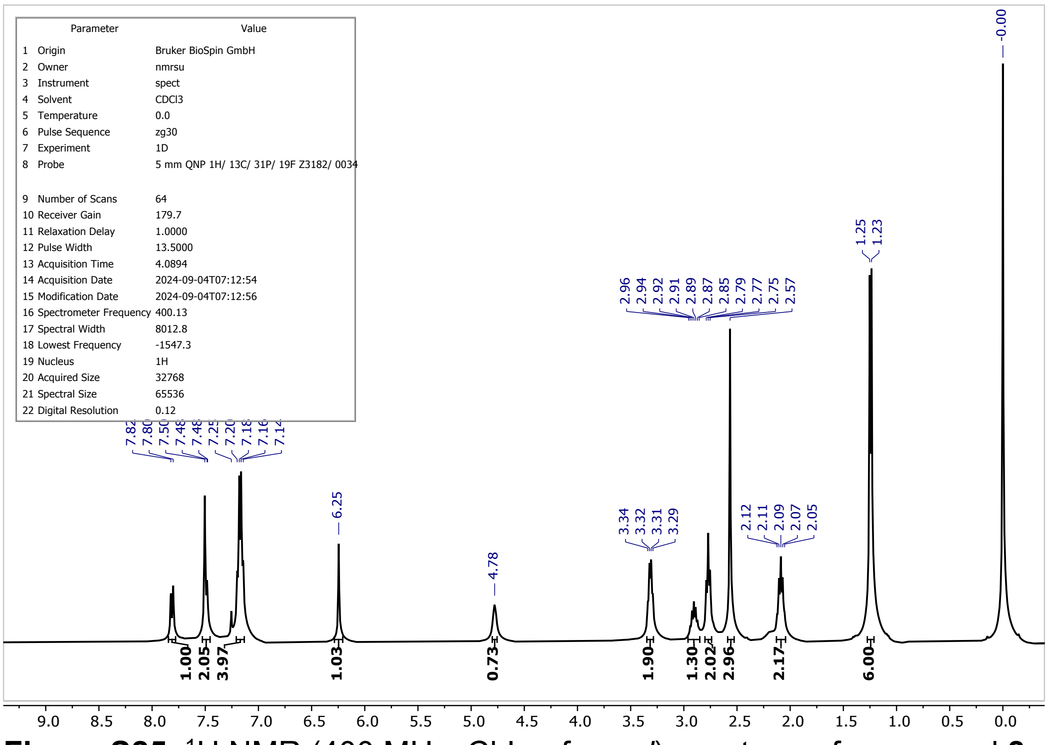

Figure S25. ^{1}H NMR (400 MHz, Chloroform-*d*) spectrum of compound **8m**.

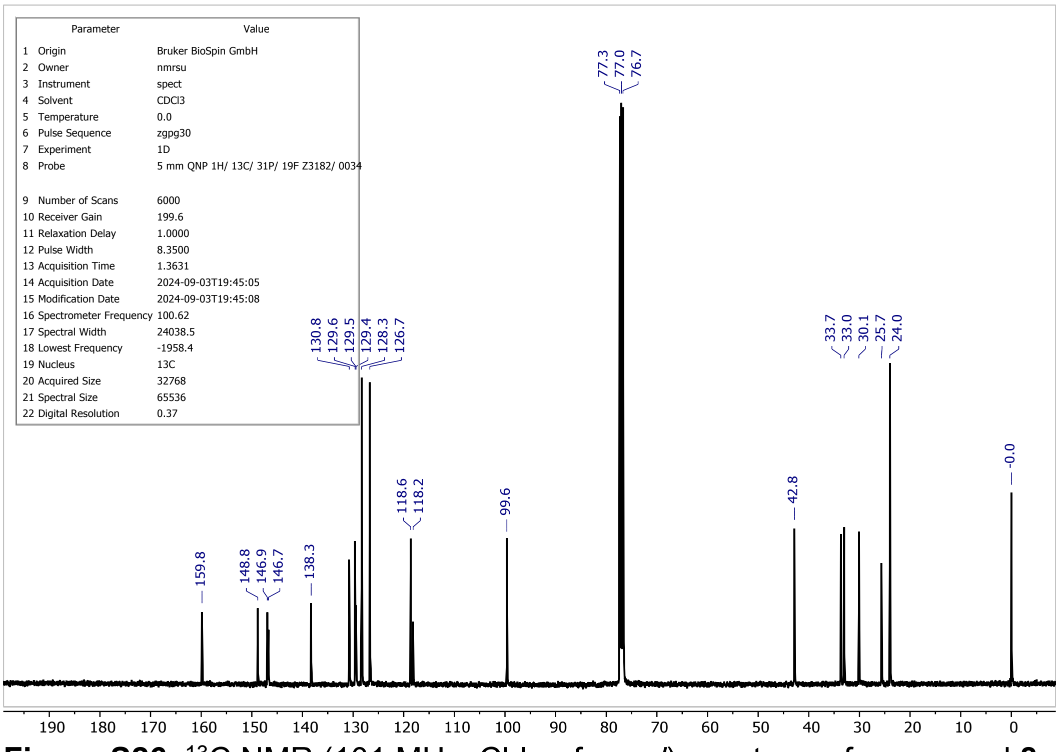

Figure S26. ^{13}C NMR (101 MHz, Chloroform-*d*) spectrum of compound **8m**.

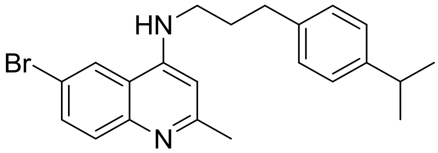

6-Bromo-*N*-(3-(4-isopropylphenyl)propyl)-2-methylquinolin-4-amine (**8n**)

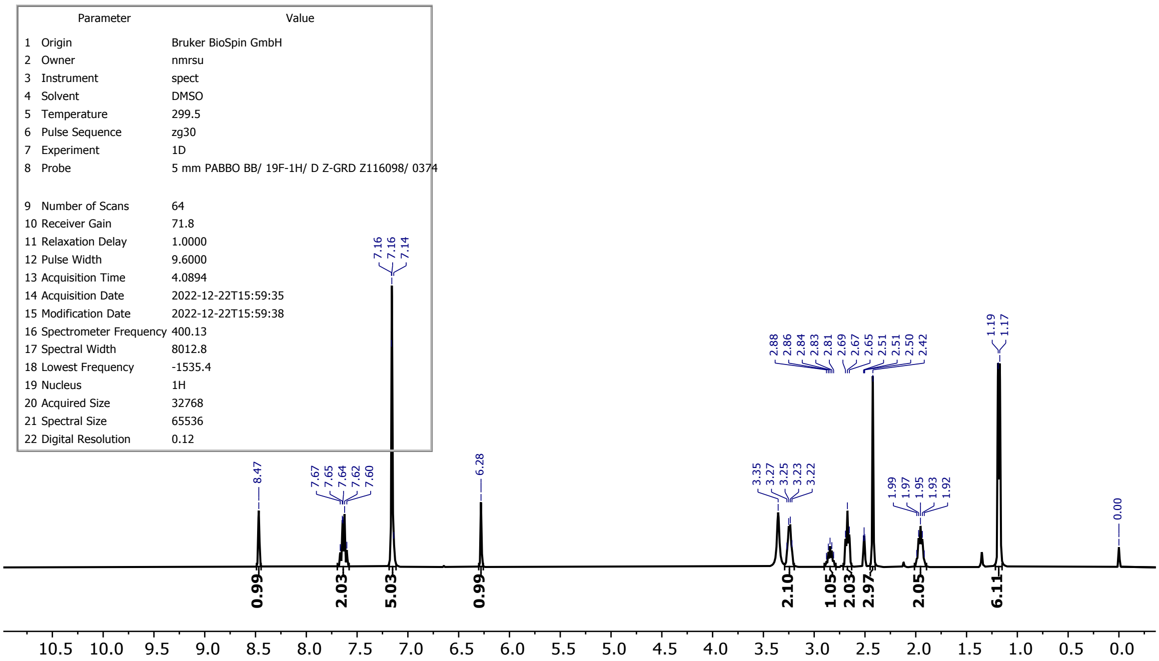

Figure S27. ^{1}H NMR (400 MHz, DMSO-d_6) spectrum of compound **8n**.

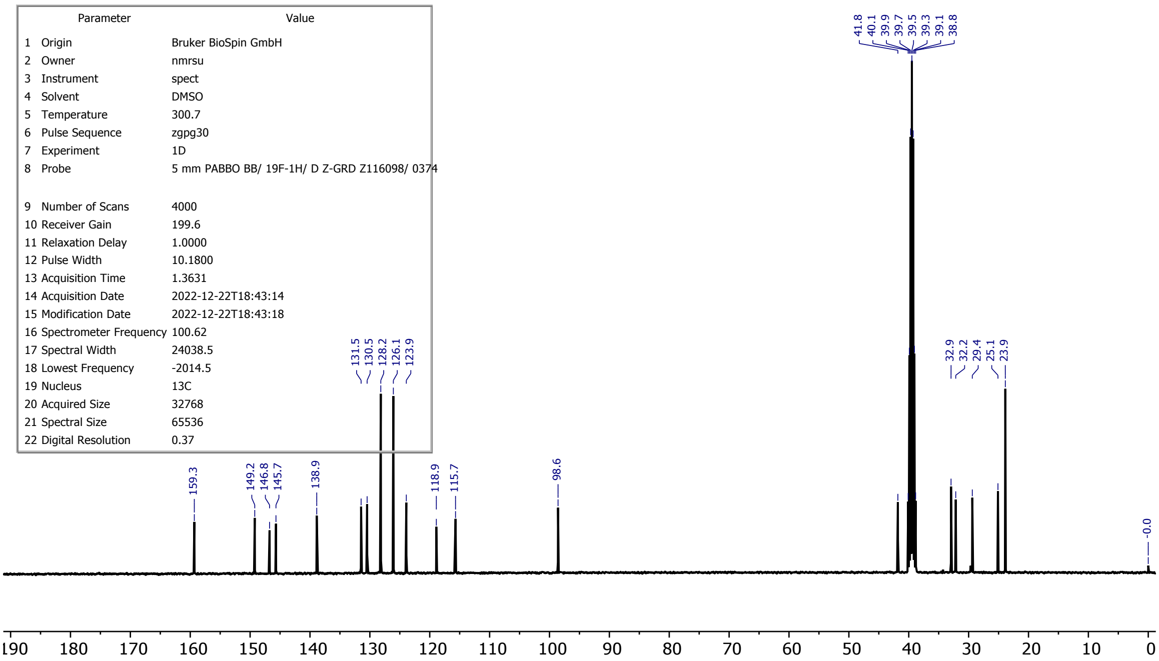

Figure S28. ^{13}C NMR (101 MHz, DMSO-d_6) spectrum of compound **8n**.

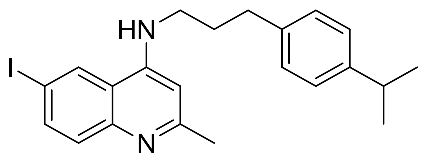

6-Iodo-*N*-(3-(4-isopropylphenyl)propyl)-2-methylquinolin-4-amine (**8o**)

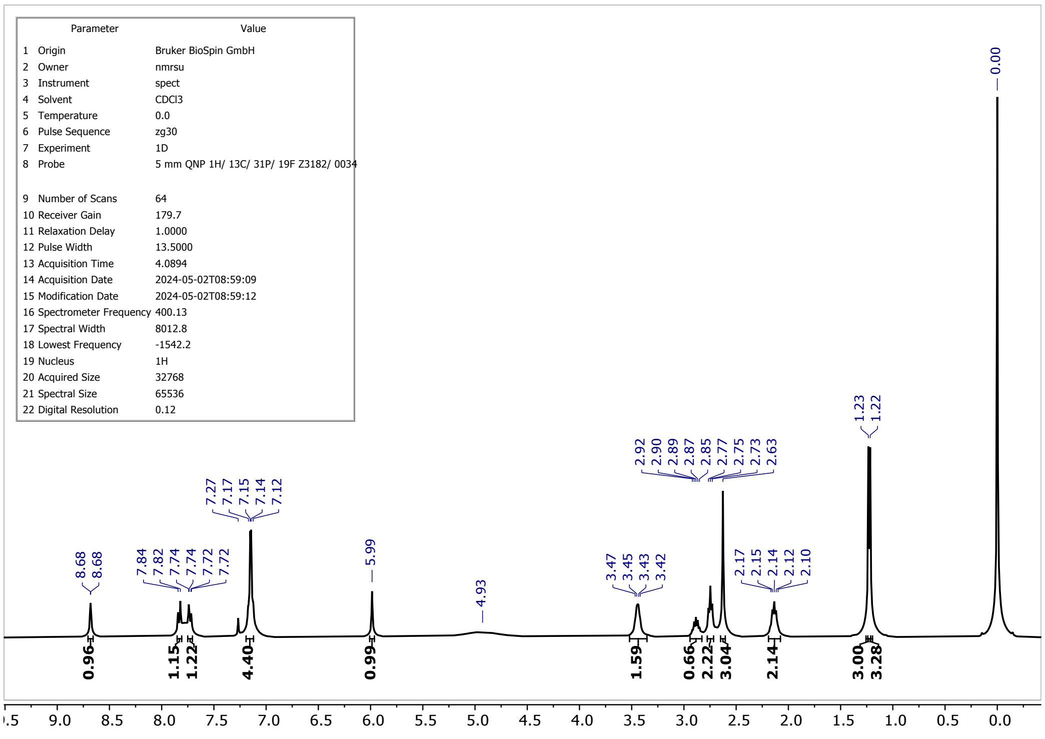

Figure S29. ^{1}H NMR (400 MHz, Chloroform-*d*) spectrum of compound **8o**.

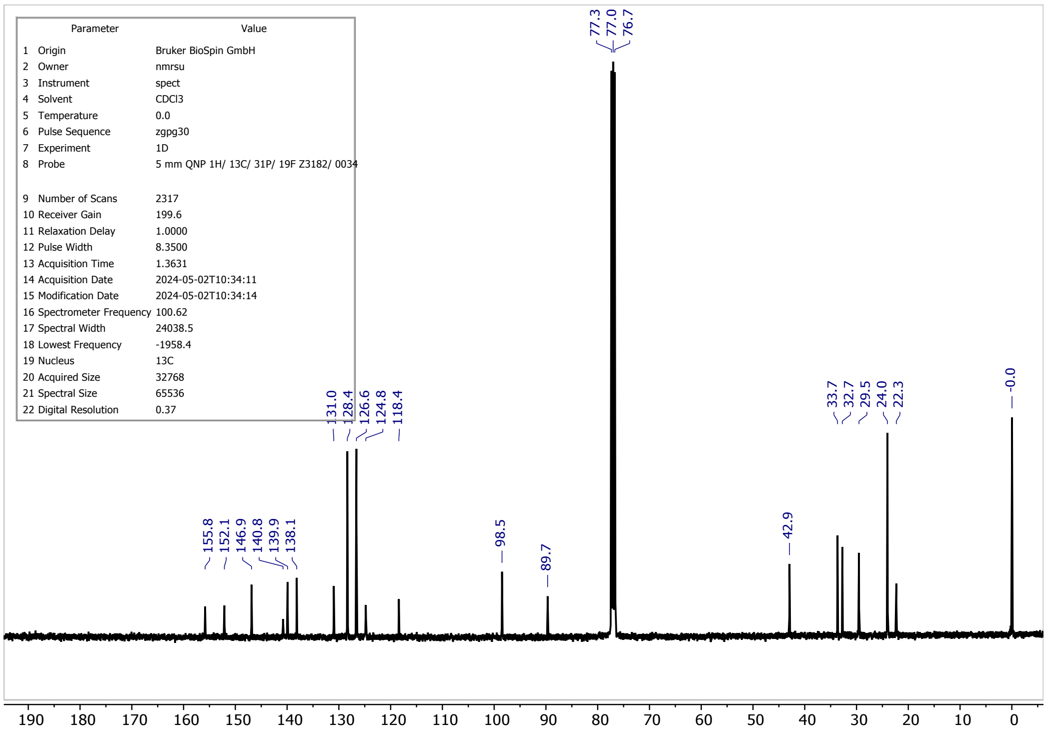

Figure S30. ^{13}C NMR (101 MHz, Chloroform-*d*) spectrum of compound **8o**.

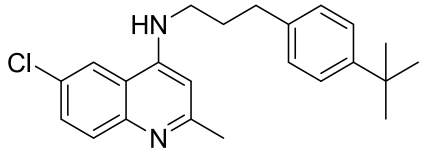

N-(3-(4-(Tert-butyl)phenyl)propyl)-6-chloro-2-methylquinolin-4-amine (**8p**)

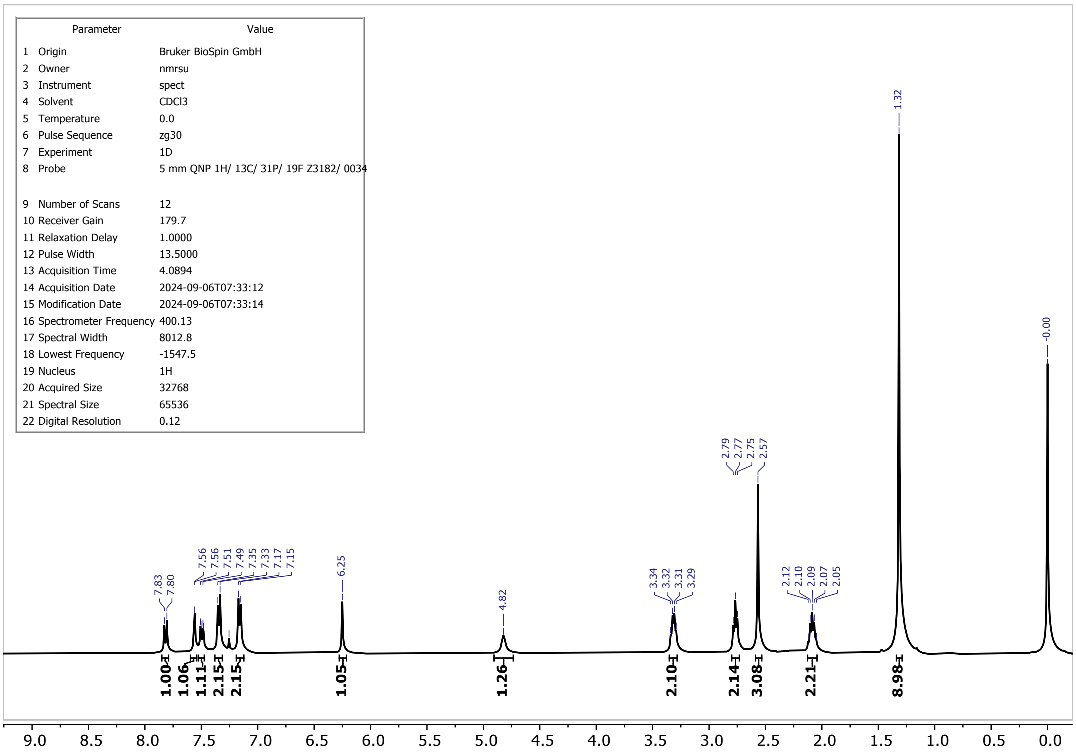

Figure S31. ^{1}H NMR (400 MHz, Chloroform-*d*) spectrum of compound **8p**.

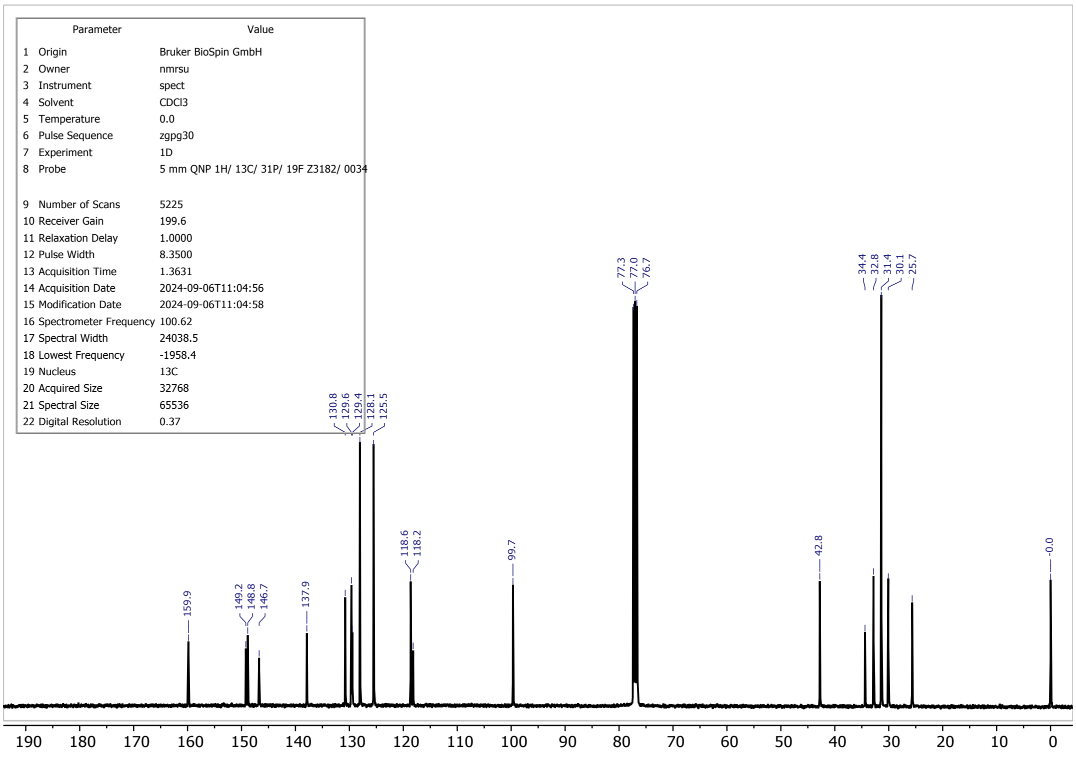

Figure S32. ^{13}C NMR (101 MHz, Chloroform-*d*) spectrum of compound **8p**.

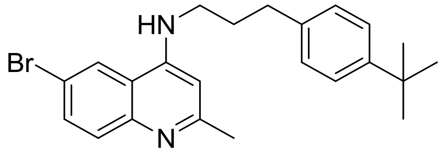

6-Bromo-*N*-(3-(4-(tert-butyl)phenyl)propyl)-2-methylquinolin-4-amine (**8q**)

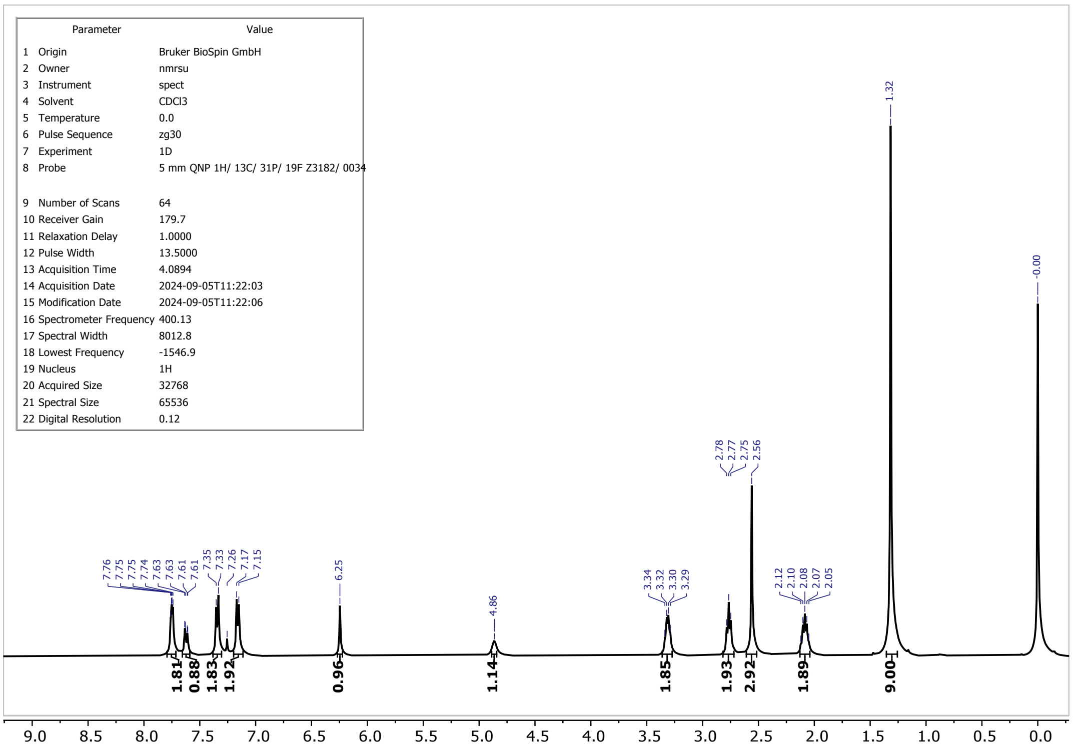

Figure S33. ^{1}H NMR (400 MHz, Chloroform-*d*) spectrum of compound **8q**.

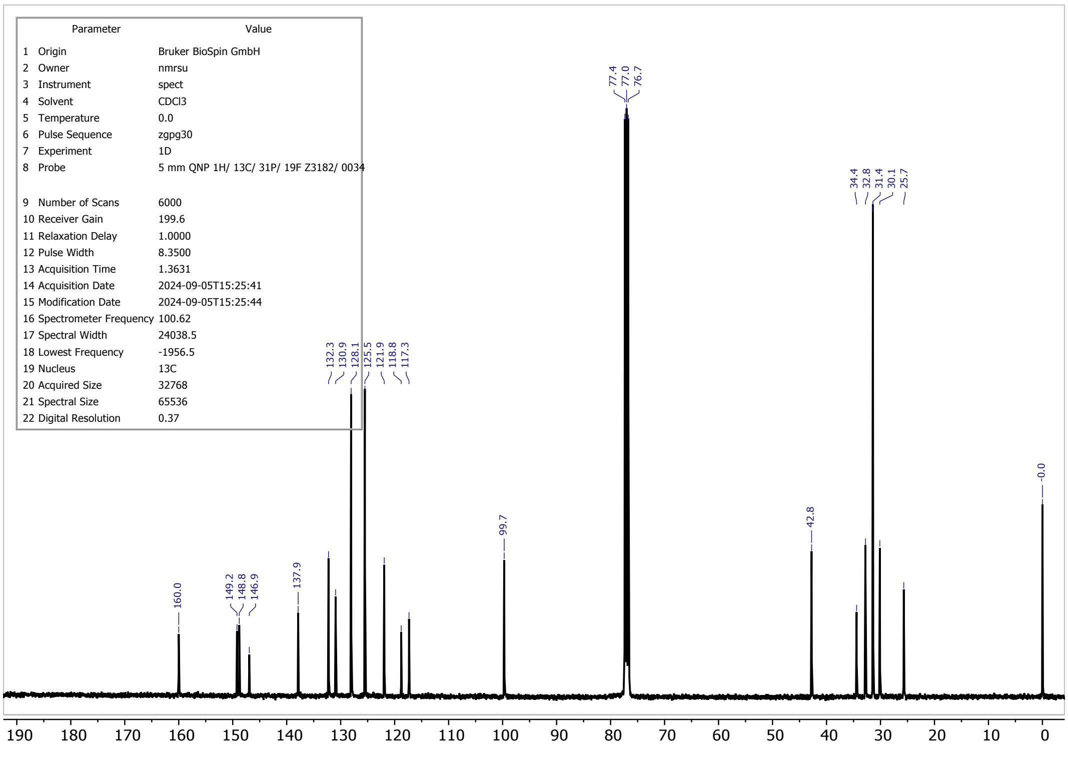

Figure S34. ^{13}C NMR (101 MHz, Chloroform-*d*) spectrum of compound **8q**.

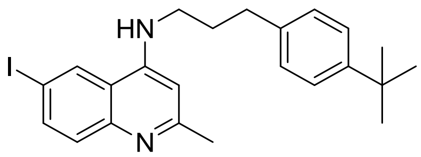

N -(3-(4-(tert-butyl)phenyl)propyl)-6-iodo-2-methylquinolin-4-amine (**8r**)

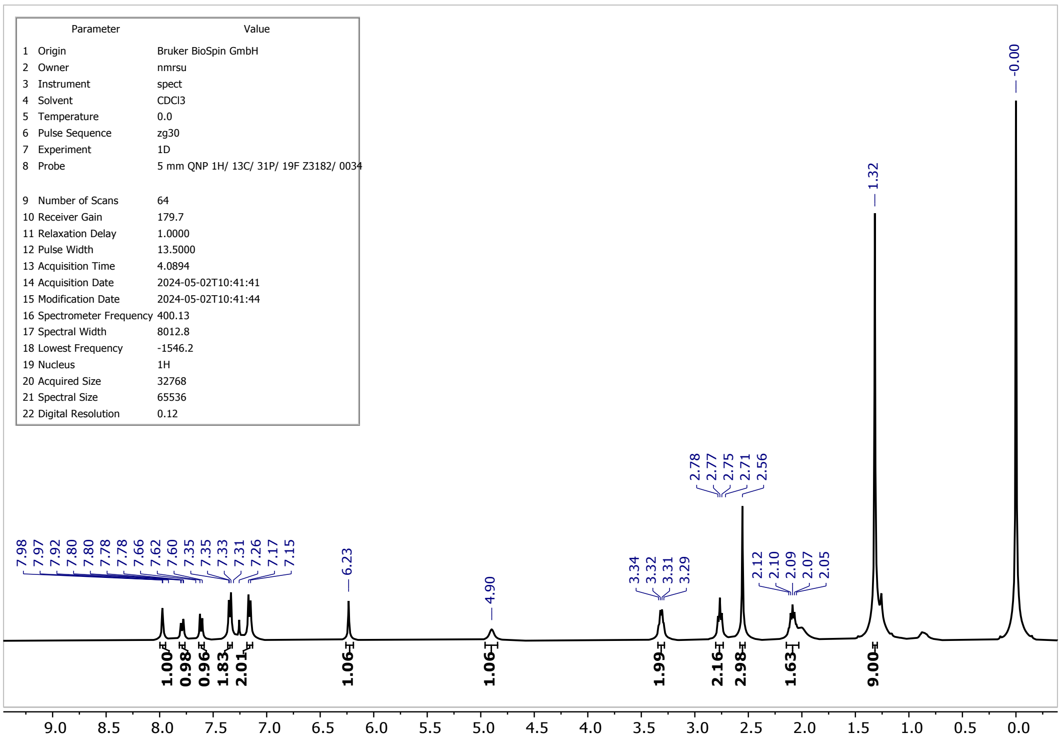

Figure S35. ^{1}H NMR (400 MHz, Chloroform-*d*) spectrum of compound **8r**.

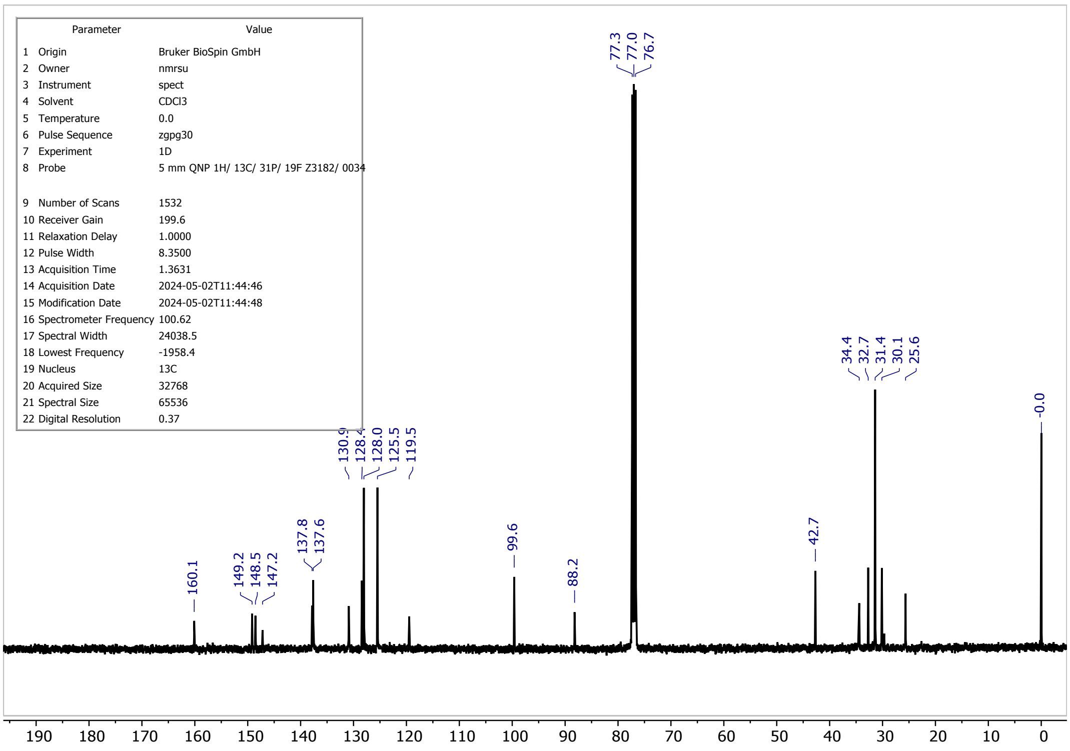

Figure S36. ^{13}C NMR (101 MHz, Chloroform-*d*) spectrum of compound **8r**.

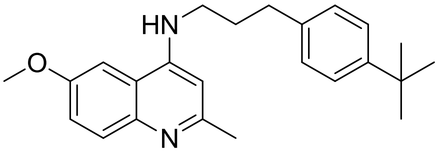

N-(3-(4-(Tert-butyl)phenyl)propyl)-6-methoxy-2-methylquinolin-4-amine (**8s**)

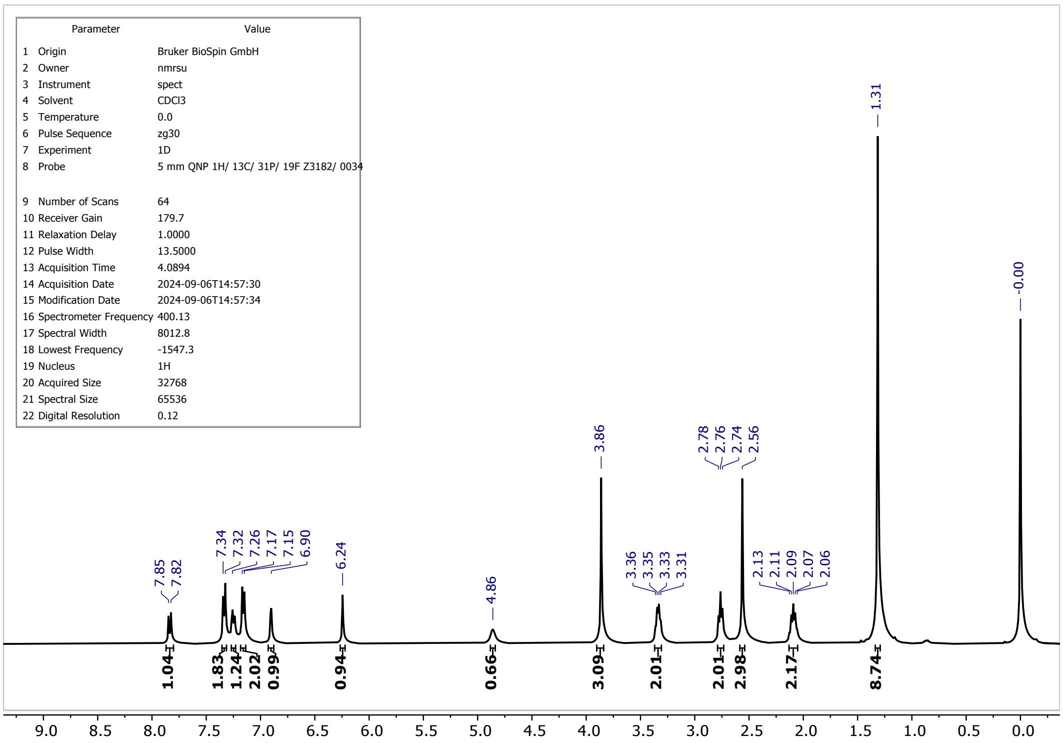

Figure S37. ^{1}H NMR (400 MHz, Chloroform-*d*) spectrum of compound **8s**.

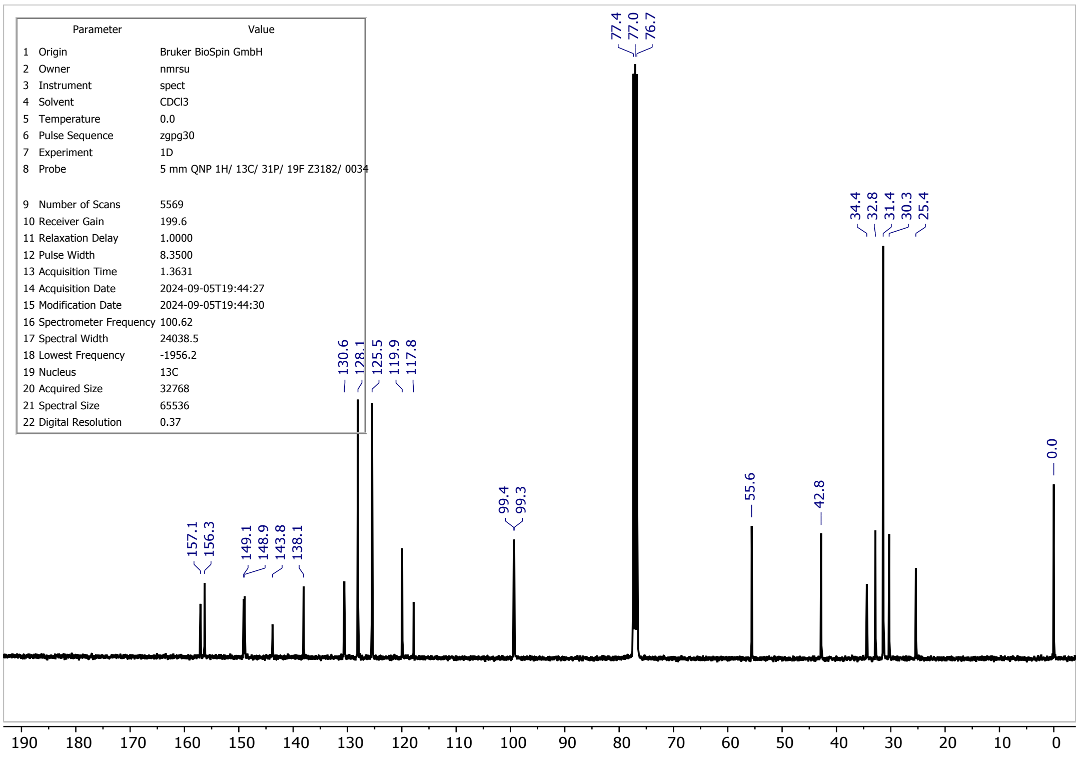

Figure S38. ^{13}C NMR (101 MHz, Chloroform-*d*) spectrum of compound **8s**.

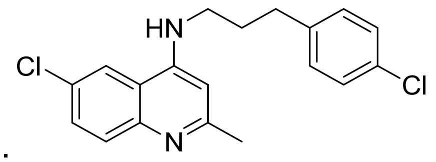

6-Chloro-*N*-(3-(4-chlorophenyl)propyl)-2-methylquinolin-4-amine (**8t**)

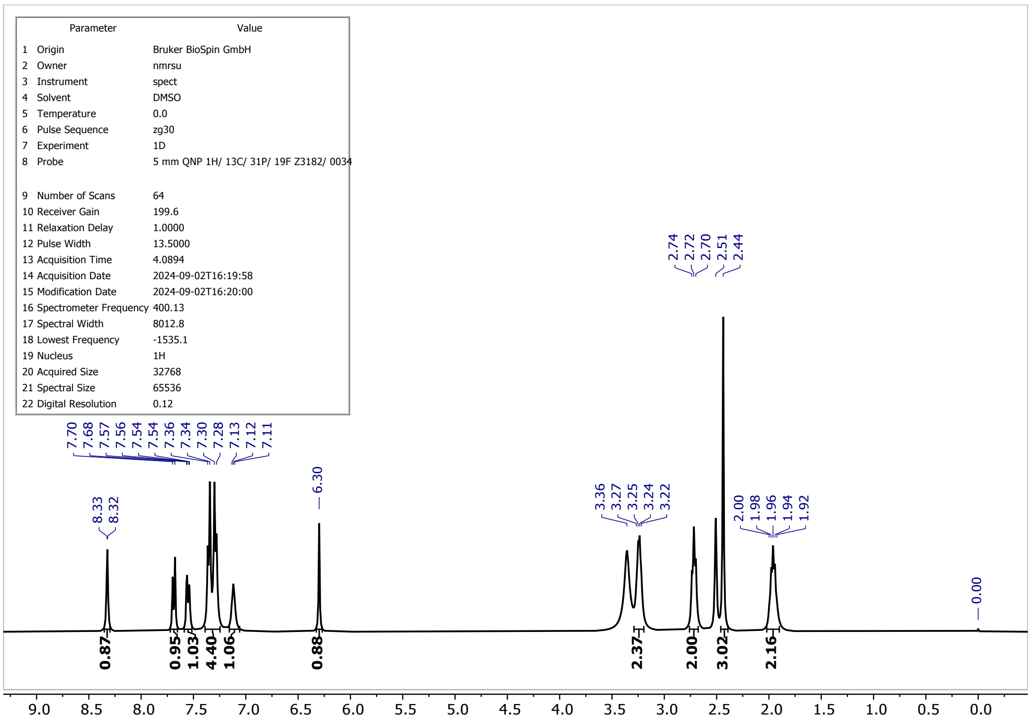

Figure S39. ^{1}H NMR (400 MHz, DMSO-d_6) spectrum of compound **8t**.

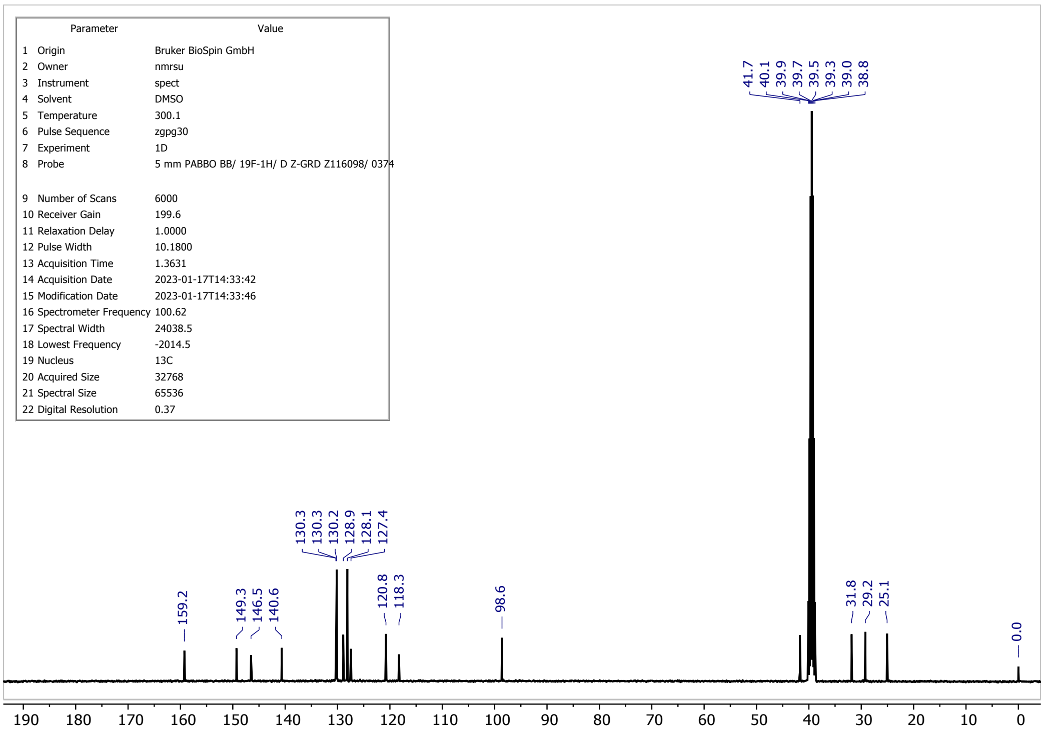

Figure S40. ^{13}C NMR (101 MHz, DMSO-d_6) spectrum of compound **8t**.

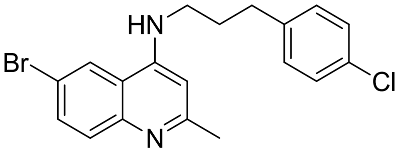

6-Bromo-*N*-(3-(4-chlorophenyl)propyl)-2-methylquinolin-4-amine (**8u**)

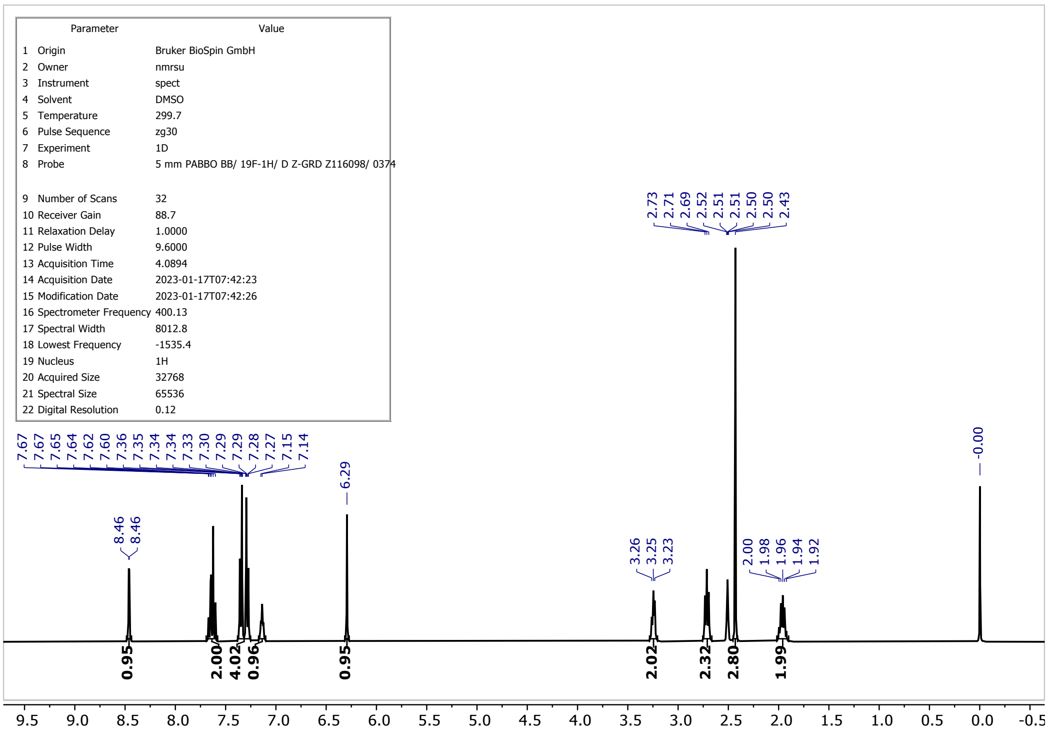

Figure S41. ^{1}H NMR (400 MHz, DMSO-d_6) spectrum of compound **8u**.

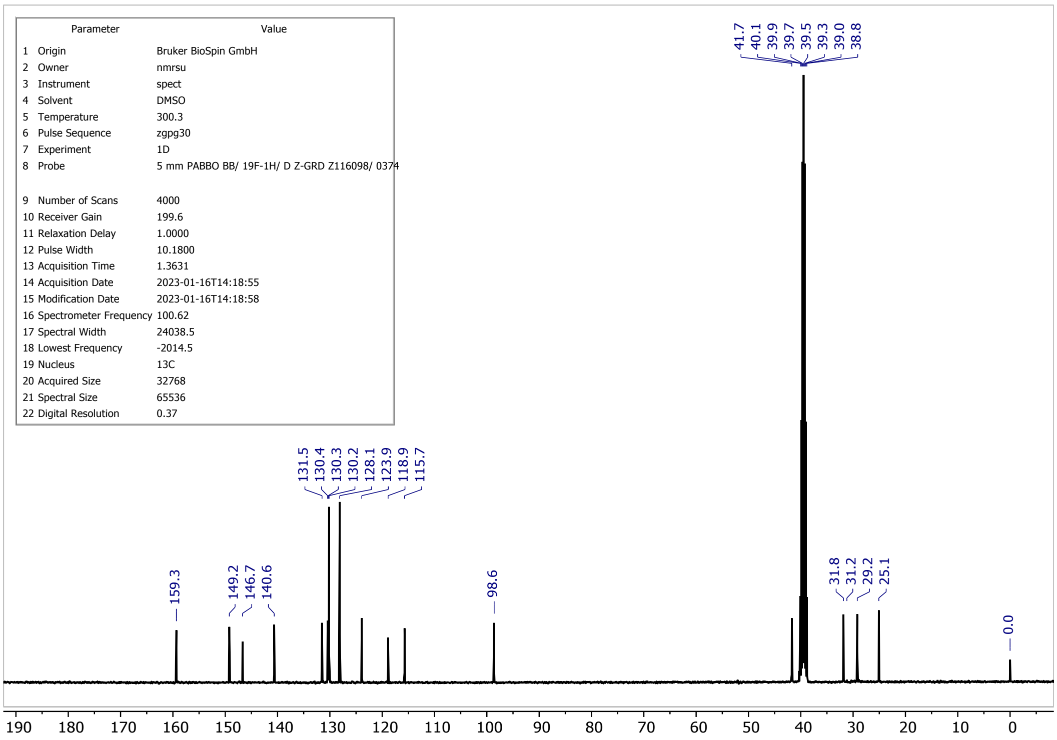

Figure S42. ^{13}C NMR (101 MHz, DMSO-d_6) spectrum of compound **8u**.

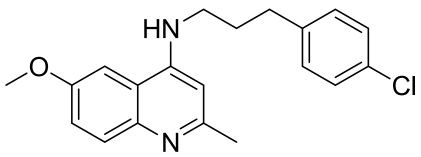

N-(3-(4-chlorophenyl)propyl)-6-methoxy-2-methylquinolin-4-amine (**8v**)

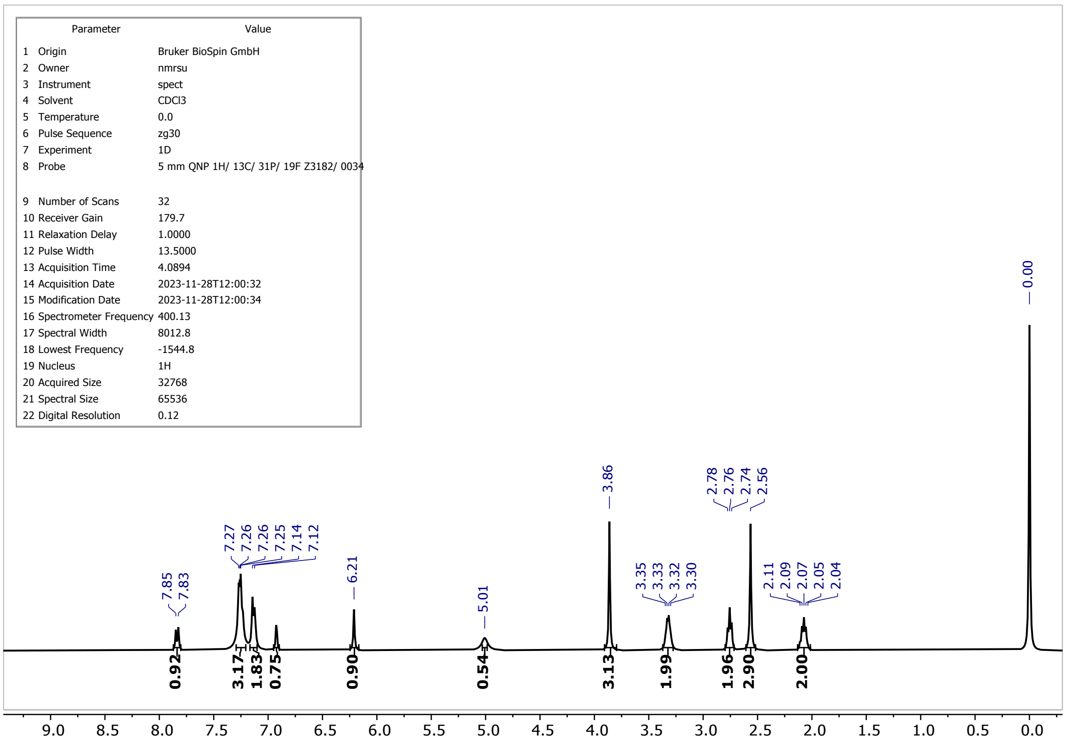

Figure S43. ^{1}H NMR (400 MHz, Chloroform-*d*) spectrum of compound **8v**.

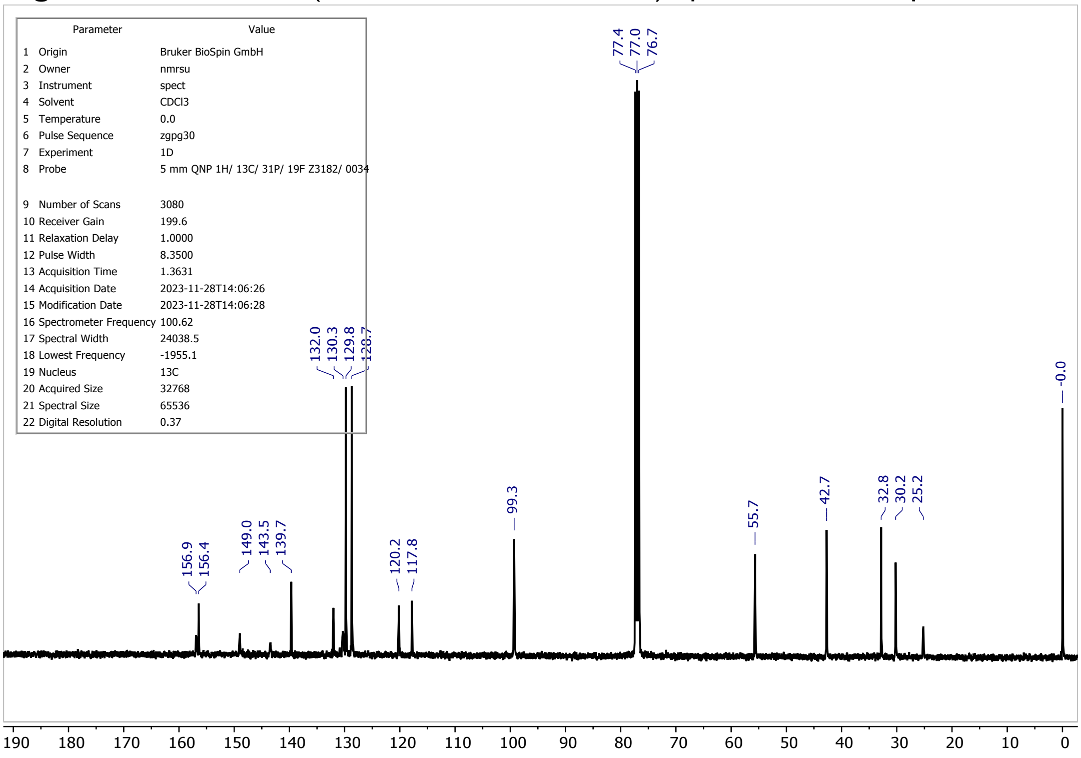

Figure S44. ^{13}C NMR (101 MHz, Chloroform-*d*) spectrum of compound **8v**.

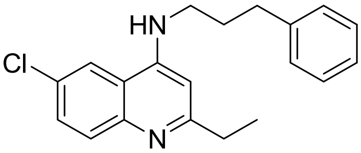

6-Chloro-2-ethyl-*N*-(3-phenylpropyl)quinolin-4-amine (**8x**)

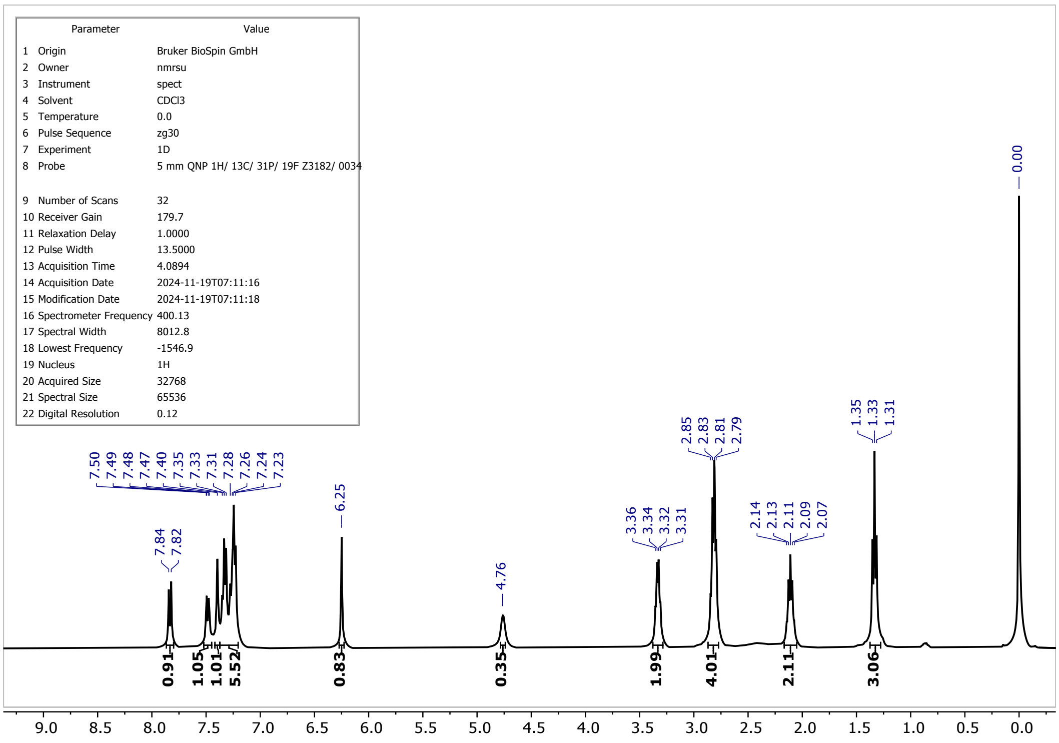

Figure S45. ^{1}H NMR (400 MHz, Chloroform-d) spectrum of compound **8x**.

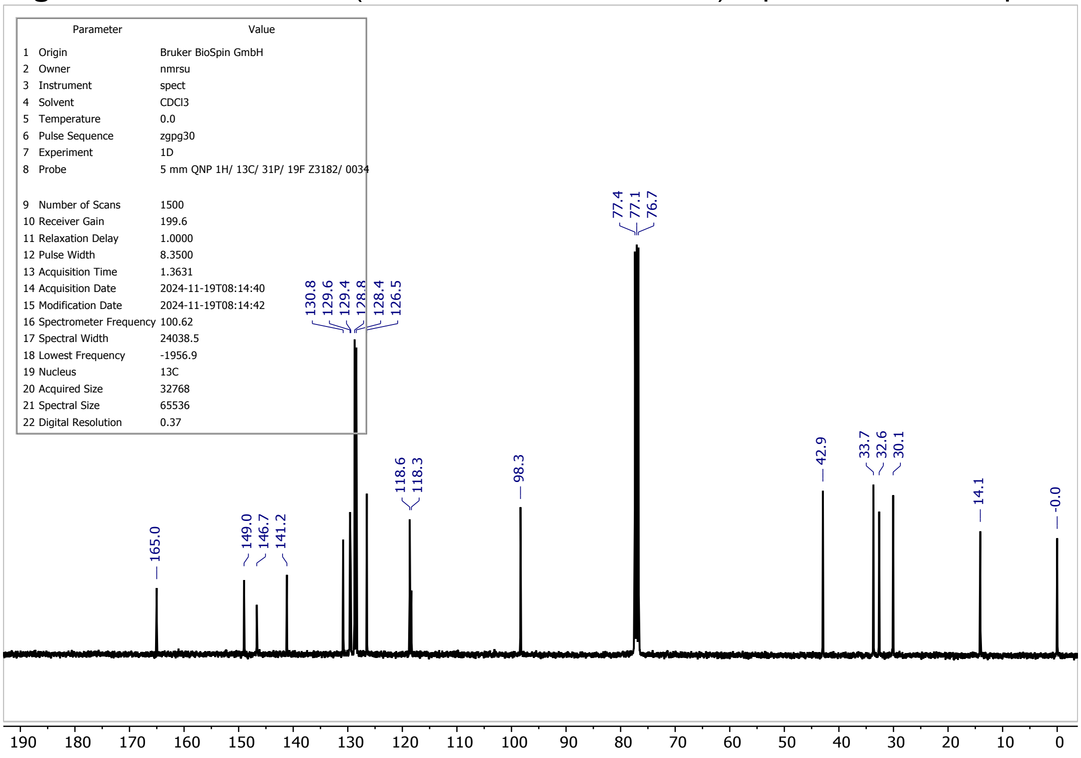

Figure S46. ^{13}C NMR (101 MHz, Chloroform-*d*) spectrum of compound **8x**.

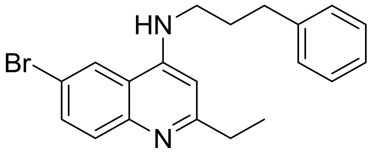

6-Bromo-2-ethyl-*N*-(3-phenylpropyl)quinolin-4-amine (**8y**)

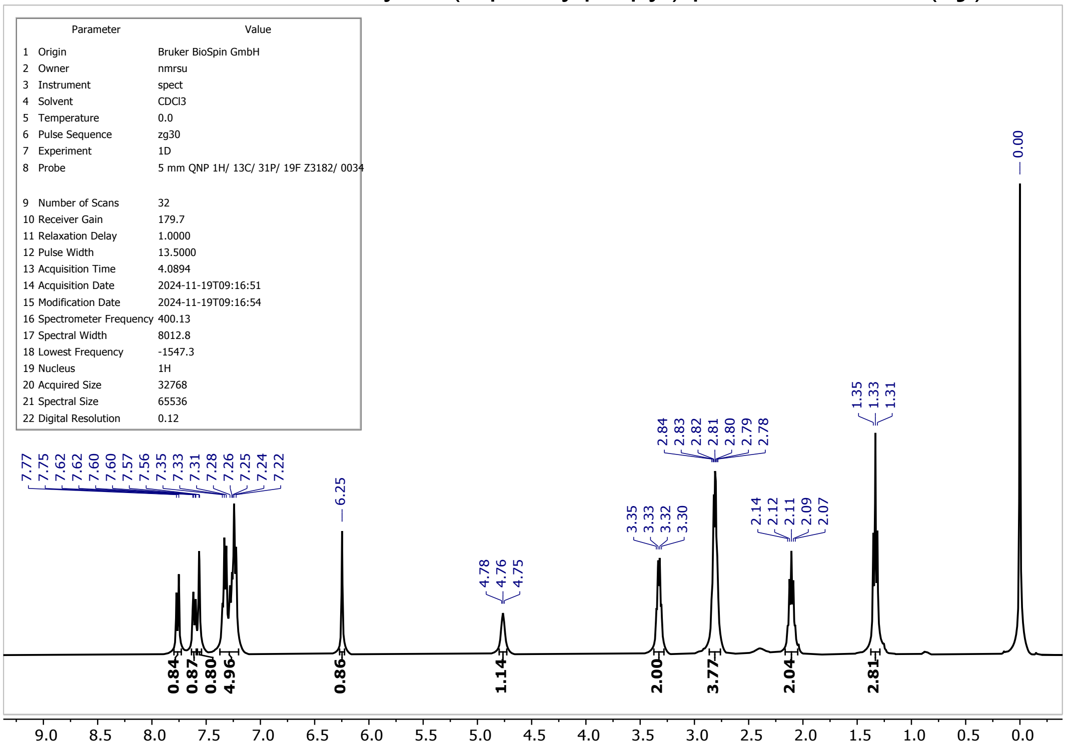

Figure S47. ^{1}H NMR (400 MHz, Chloroform-*d*) spectrum of compound **8y**.

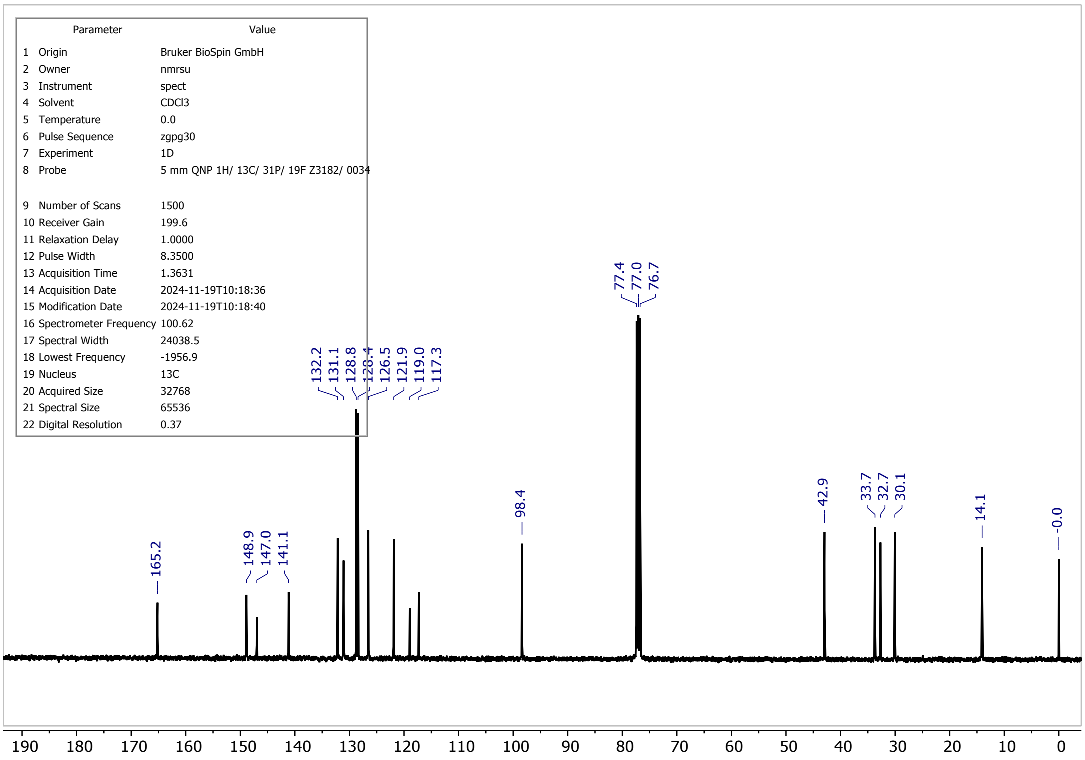

Figure S48. ^{13}C NMR (101 MHz, Chloroform-*d*) spectrum of compound **8y**.

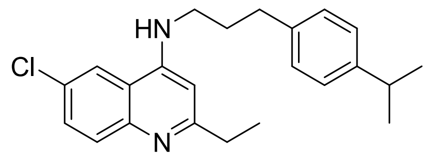

6-Chloro-2-ethyl-*N*-(3-(4-isopropylphenyl)propyl)quinolin-4-amine (**8w**)

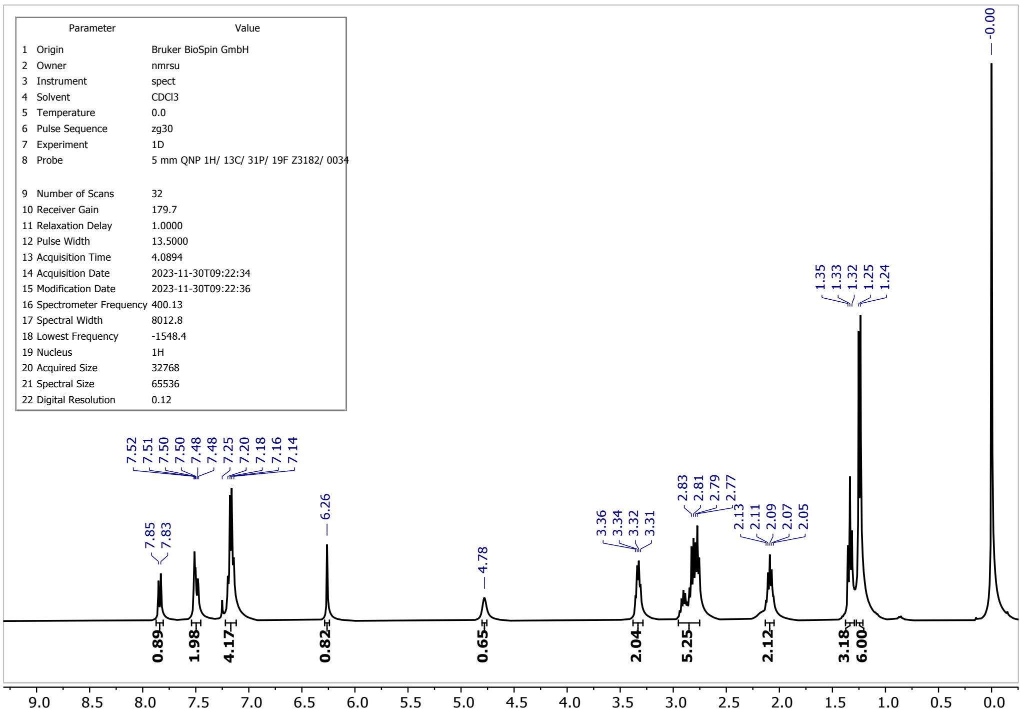

Figure S49. ^{1}H NMR (400 MHz, Chloroform-*d*) spectrum of compound **8w**.

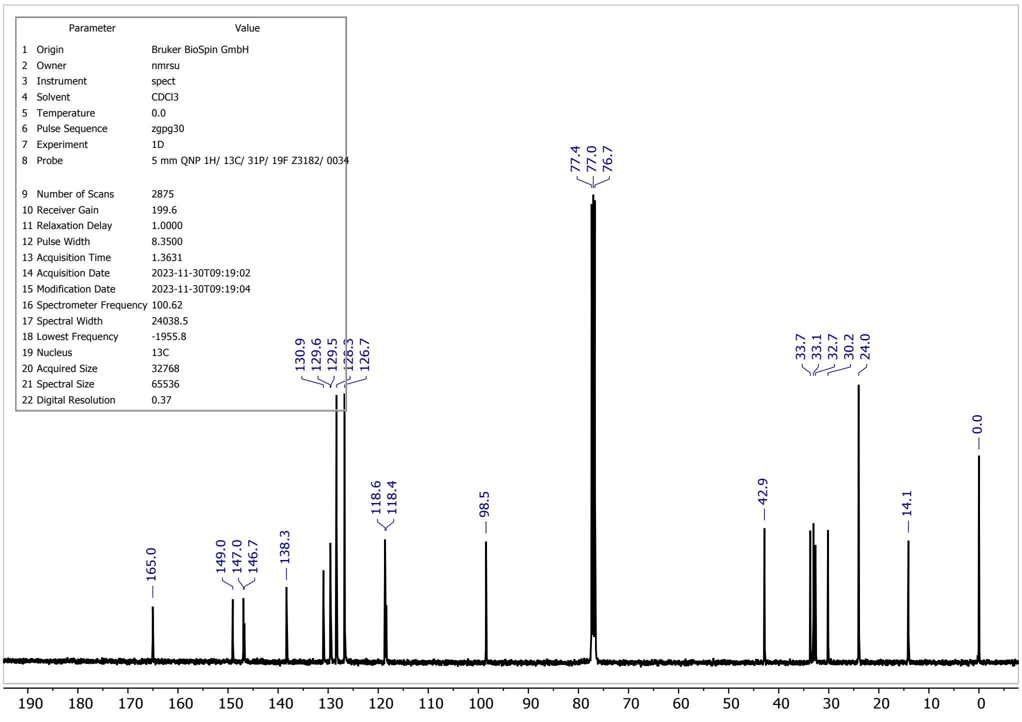

Figure S50. ^{13}C NMR (101 MHz, Chloroform-*d*) spectrum of compound **8w**.

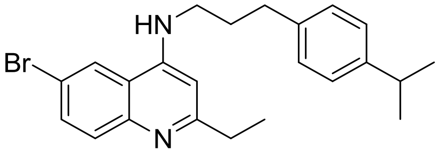

6- Bromo-2-ethyl-*N*-(3-(4-isopropylphenyl)propyl)quinolin-4-amine (**8z**)

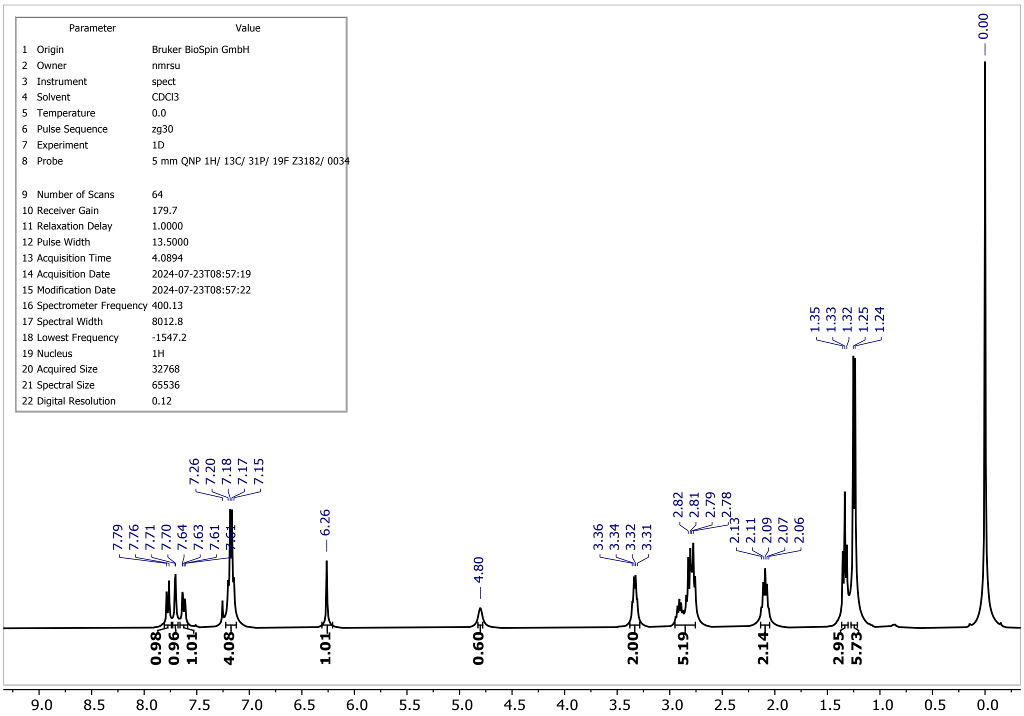

Figure S51. ^{1}H NMR (400 MHz, Chloroform-*d*) spectrum of compound **8z**.

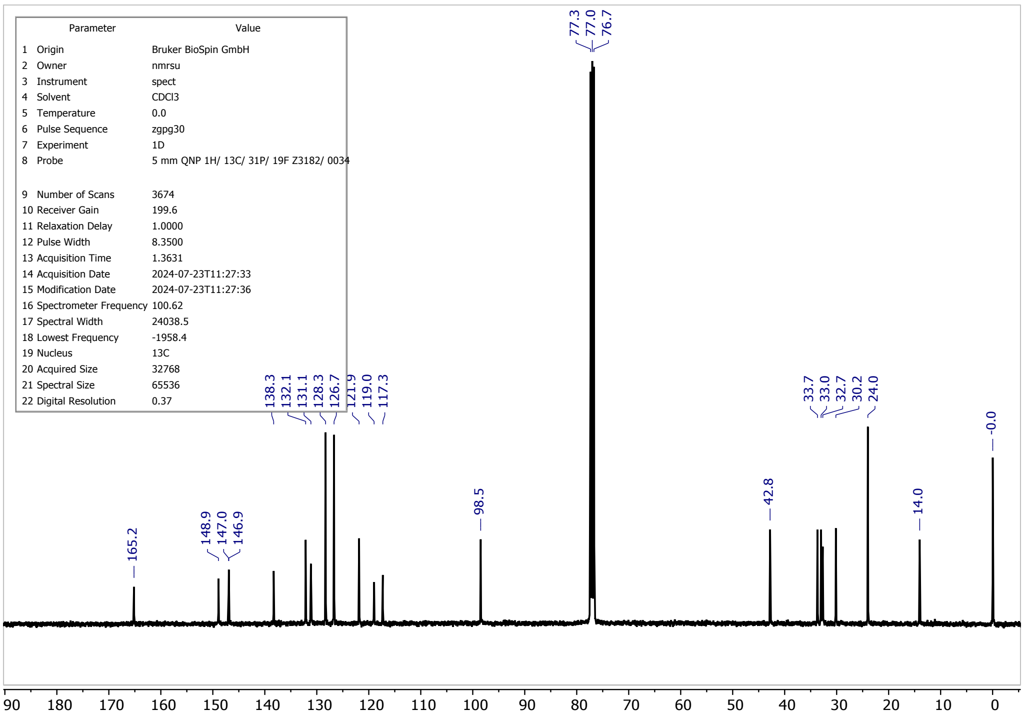

Figure S52. ^{13}C NMR (101 MHz, Chloroform-*d*) spectrum of compound **8z**.

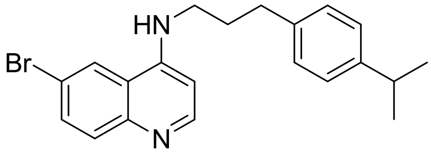

6-Bromo-*N*-(3-(4-isopropylphenyl)propyl)quinolin-4-amine (**10a**)

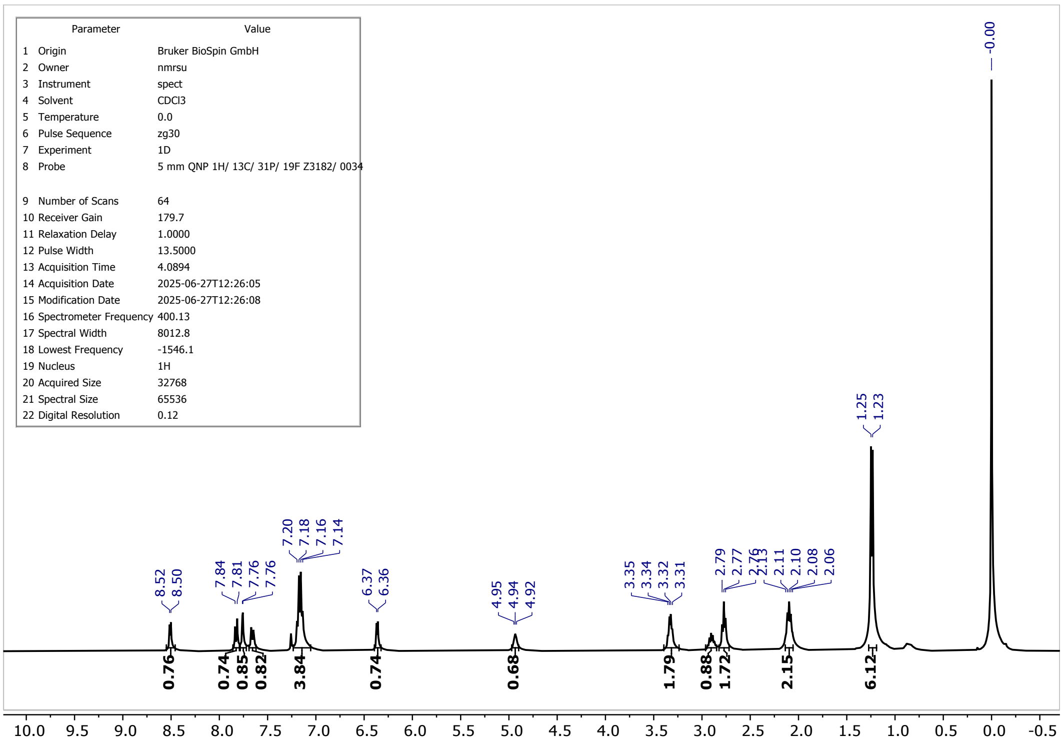

Figure S53. ^{1}H NMR (400 MHz, Chloroform-*d*) spectrum of compound **10a**.

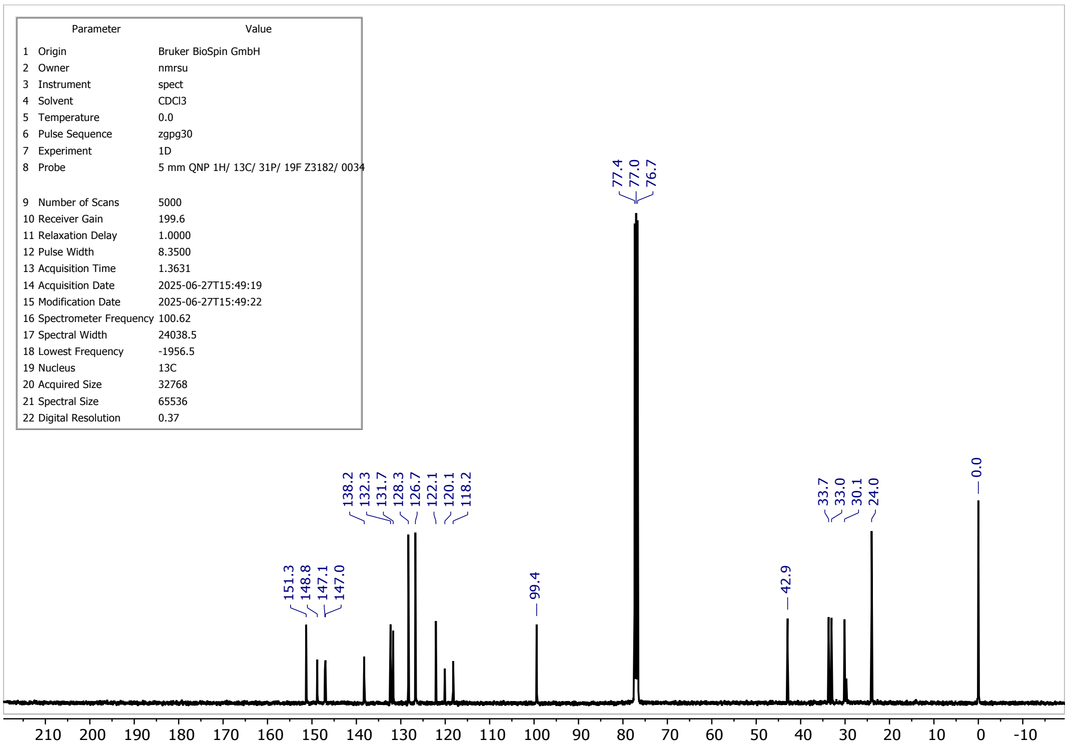

Figure S54. ^{13}C NMR (101 MHz, Chloroform-*d*) spectrum of compound **10a**.

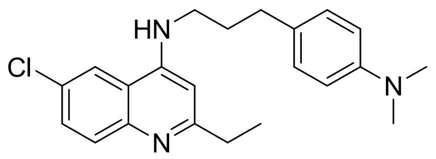

6-Chloro-*N*-(3-(4-(dimethylamino)phenyl)propyl)-2-ethylquinolin-4-amine (**10b**)

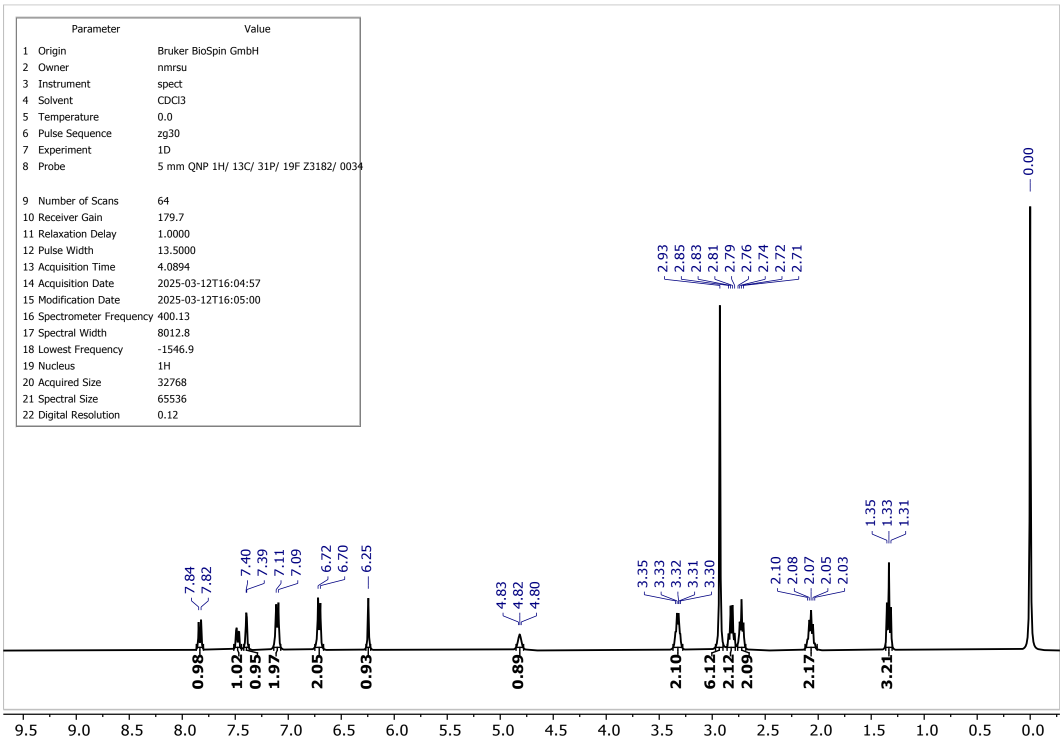

Figure S55. ^{1}H NMR (400 MHz, Chloroform-*d*) spectrum of compound **10b**.

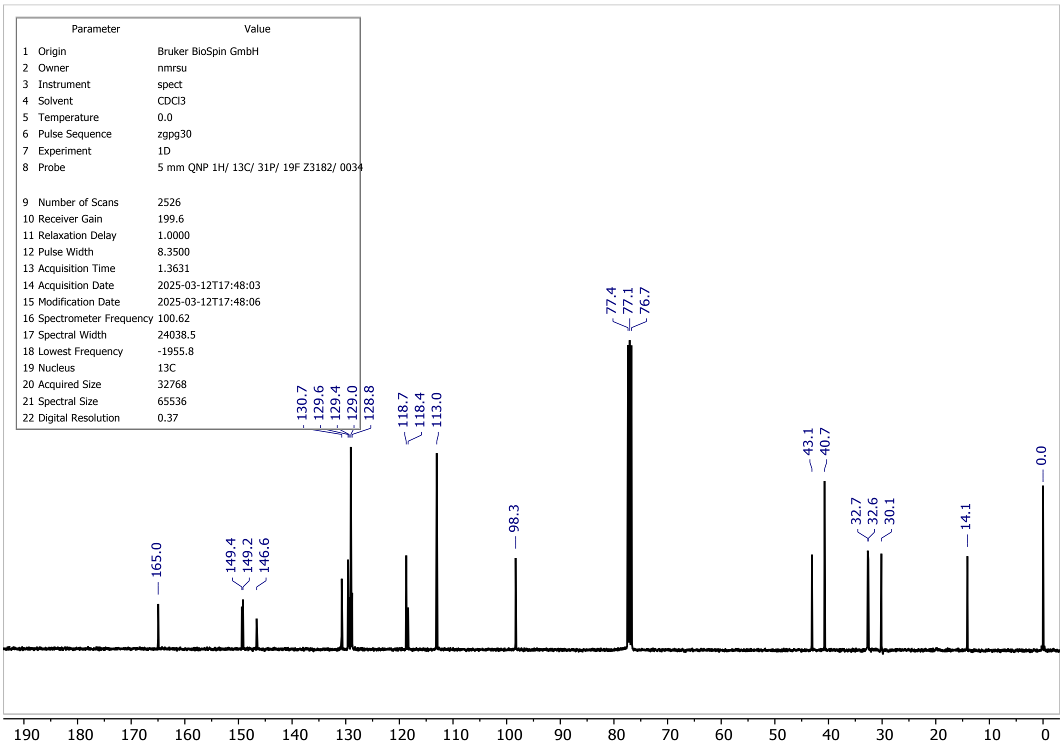

Figure S56. ^{13}C NMR (101 MHz, Chloroform-*d*) spectrum of compound **10b**.

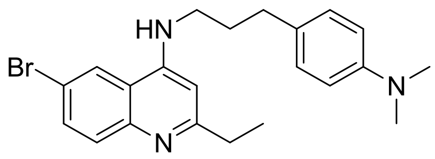

6-Bromo-*N*-(3-(4-(dimethylamino)phenyl)propyl)-2-ethylquinolin-4-amine (**10c**)

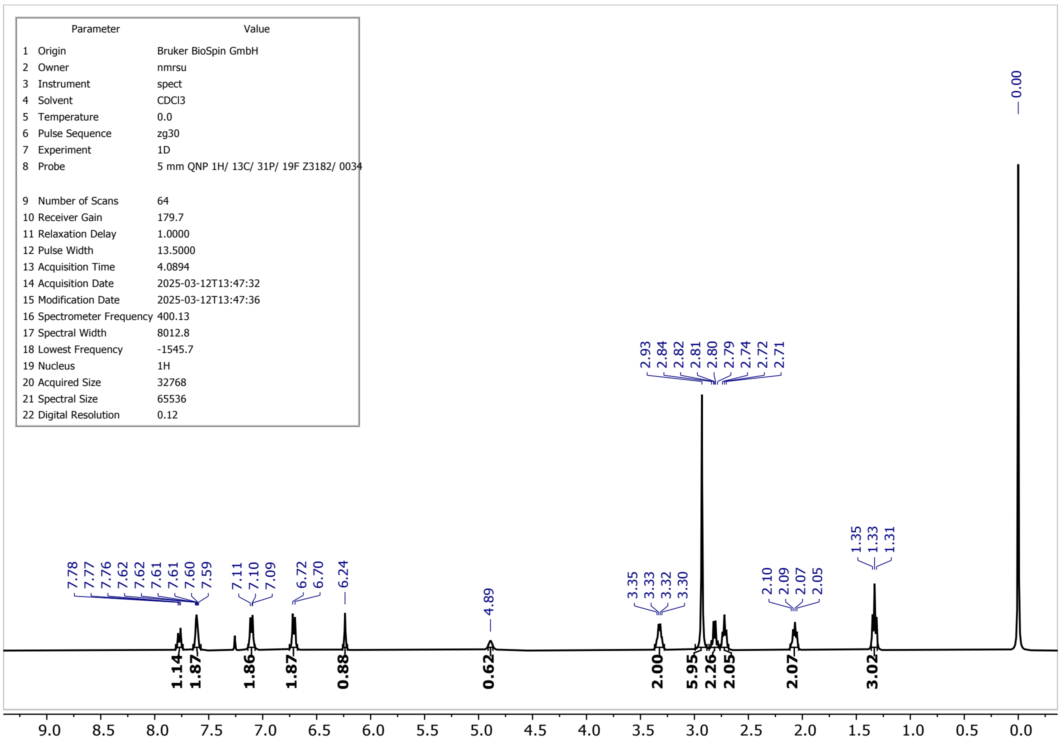

Figure S57. ^{1}H NMR (400 MHz, Chloroform-*d*) spectrum of compound **10c**.

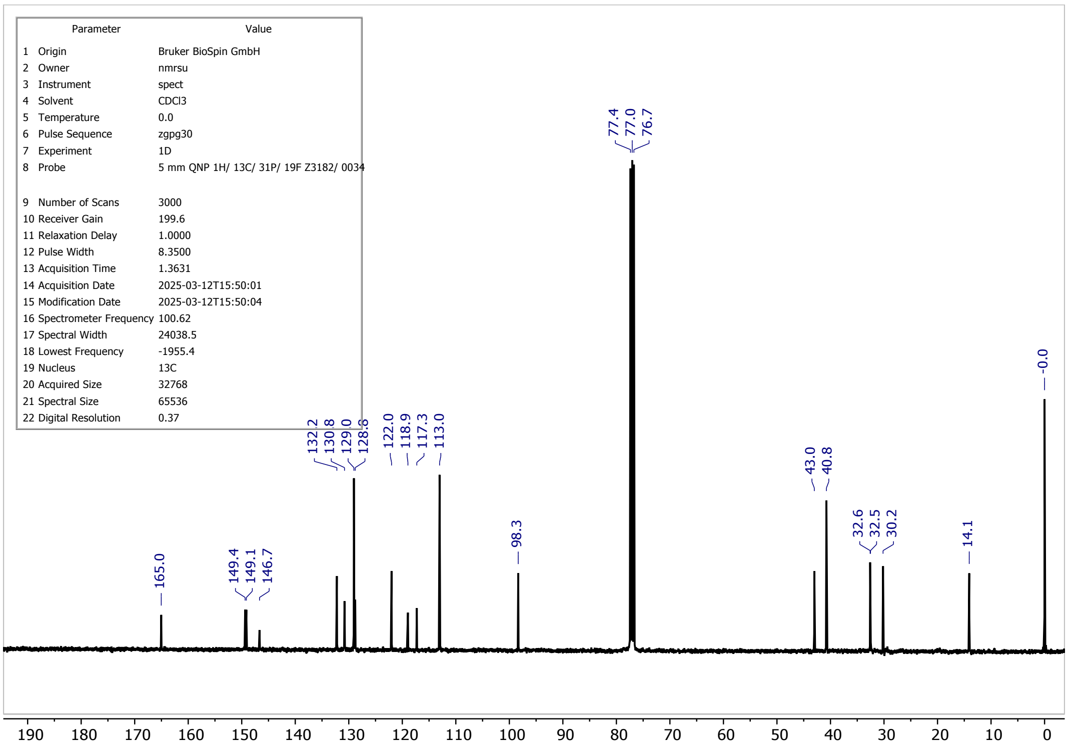

Figure S58. ^{13}C NMR (101 MHz, Chloroform-*d*) spectrum of compound **10c**.

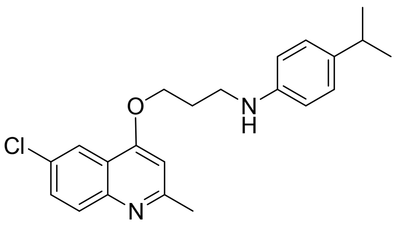

N-(3-((6-chloro-2-methylquinolin-4-yl)oxy)propyl)-4-isopropylaniline (**11a**)

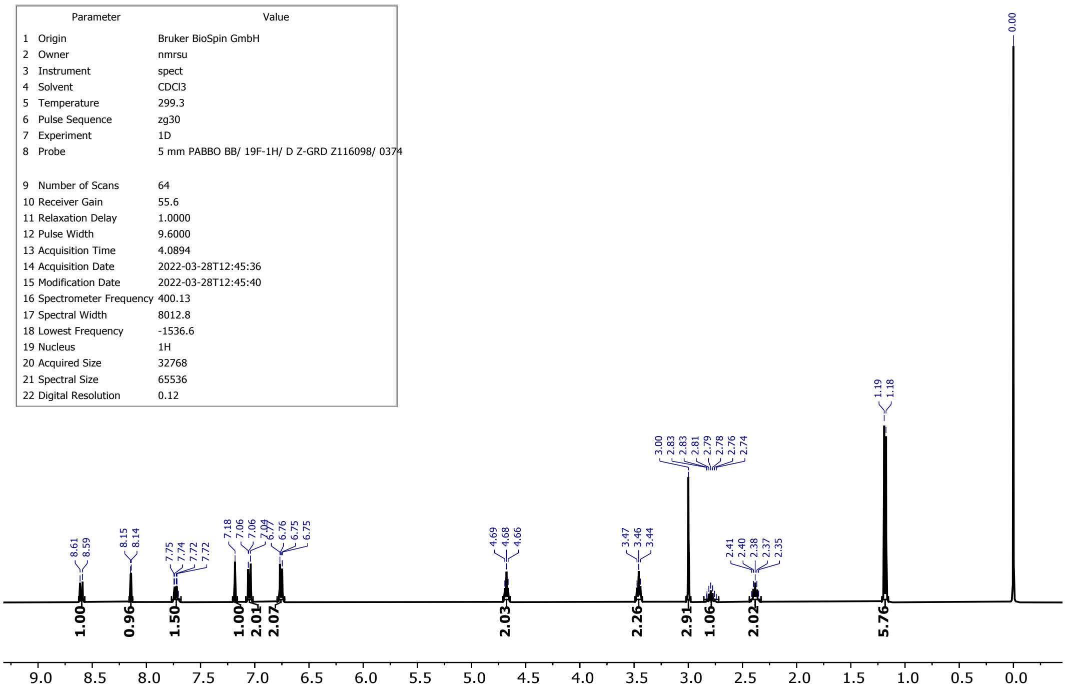

Figure S59. ^{1}H NMR (400 MHz, Chloroform-*d*) spectrum of compound **11a**.

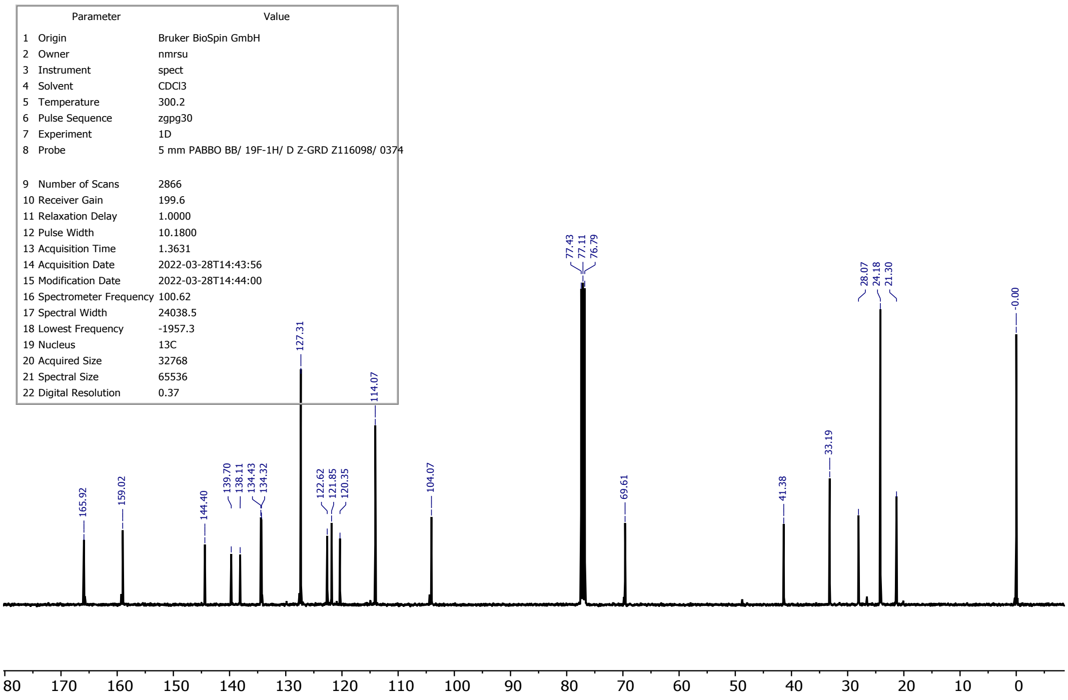

Figure S60. ^{13}C NMR (101 MHz, Chloroform-*d*) spectrum of compound **11a**.

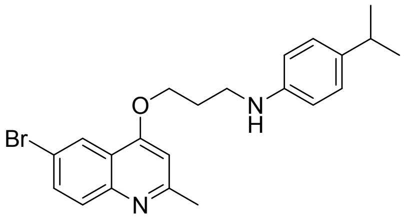

N-(3-((6-bromo-2-methylquinolin-4-yl)oxy)propyl)-4-isopropylaniline (**11b**)

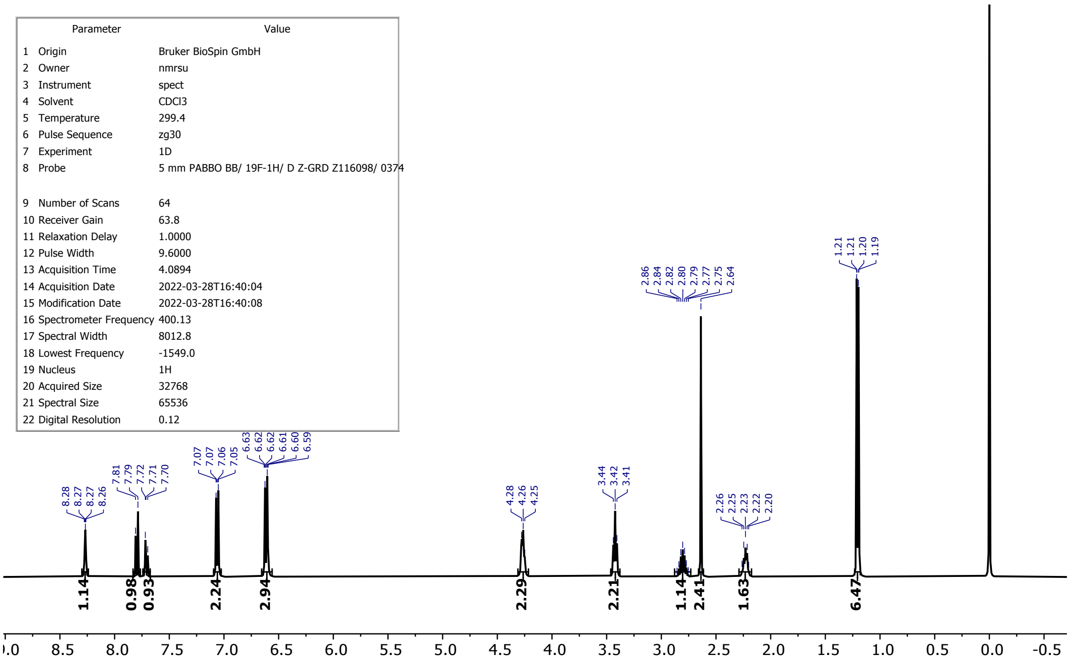

Figure S61. ^{1}H NMR (400 MHz, Chloroform-*d*) spectrum of compound **11b**.

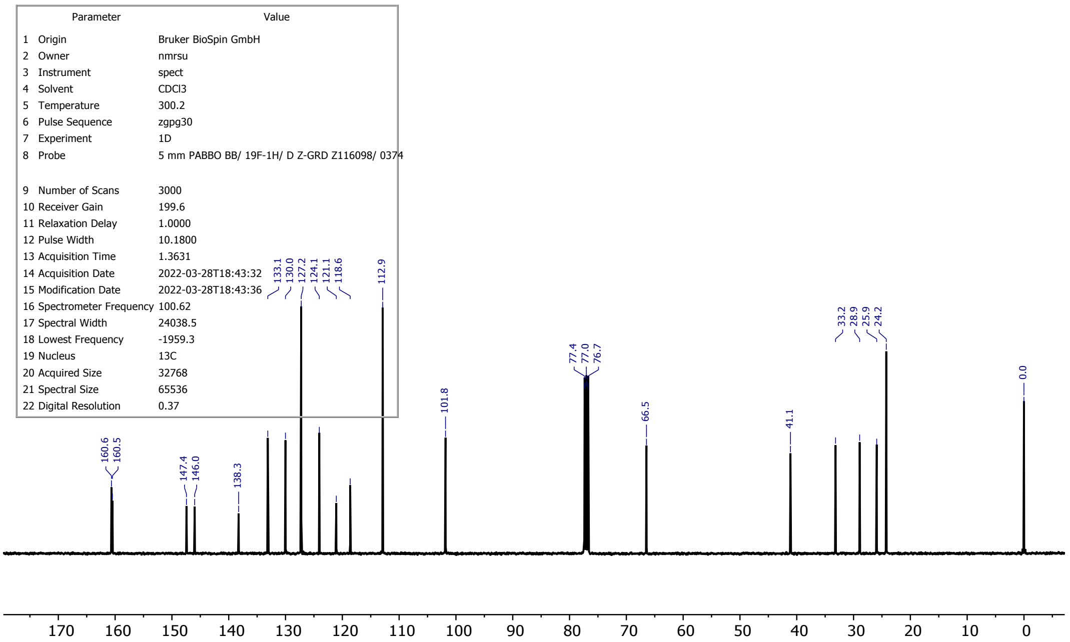

Figure S62. ^{13}C NMR (101 MHz, Chloroform-*d*) spectrum of compound **11b**.

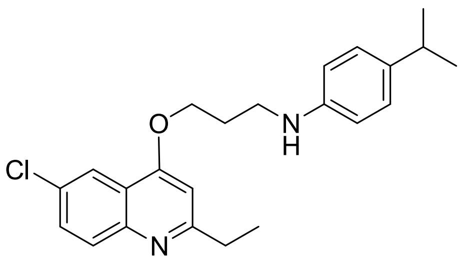

N-(3-((6-chloro-2-ethylquinolin-4-yl)oxy)propyl)-4-isopropylaniline (**11c**)

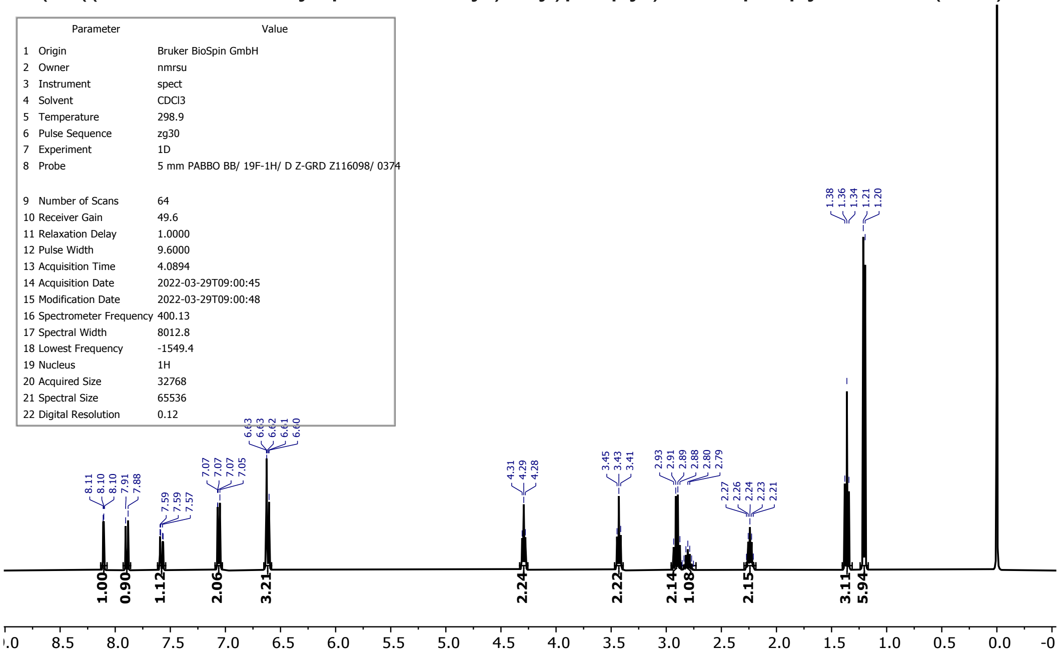

Figure S63. ^{1}H NMR (400 MHz, Chloroform-*d*) spectrum of compound **11c**.

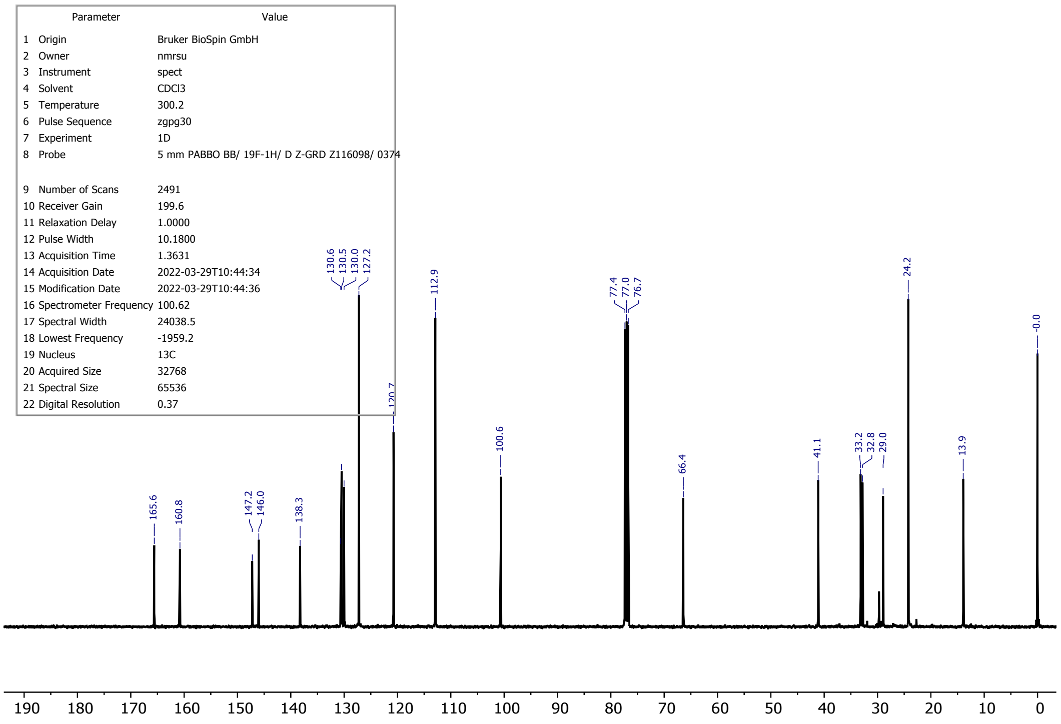

Figure S64. ^{13}C NMR (101 MHz, Chloroform-*d*) spectrum of compound **11c**.

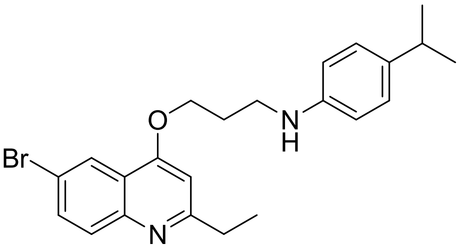

N-(3-((6-bromo-2-ethylquinolin-4-yl)oxy)propyl)-4-isopropylaniline (**11d**)

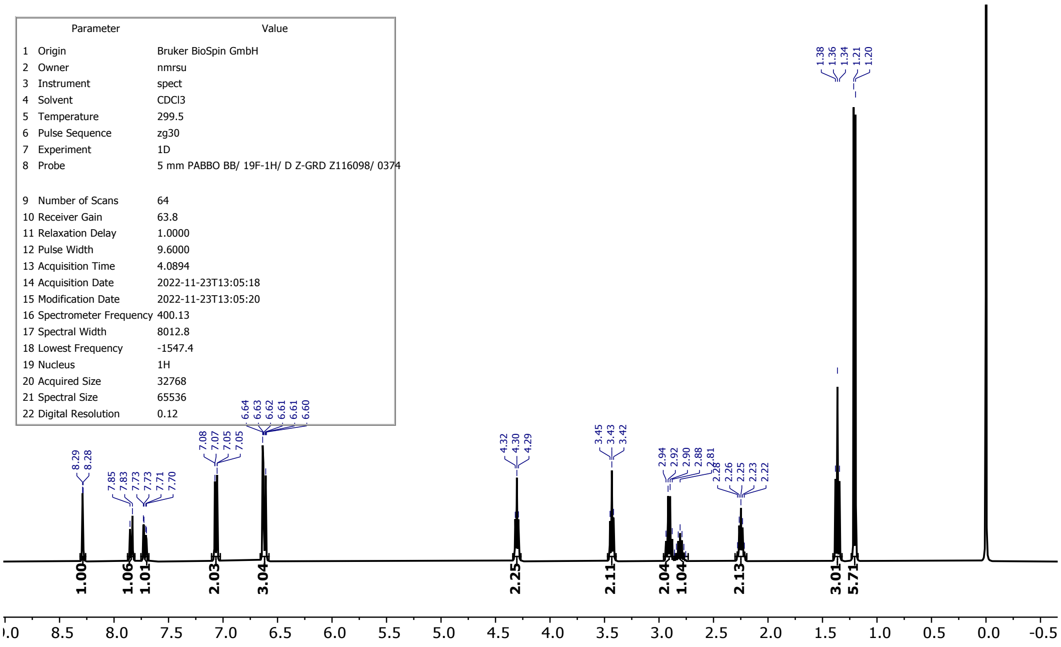

Figure S65. ^{1}H NMR (400 MHz, Chloroform-*d*) spectrum of compound **11d**.

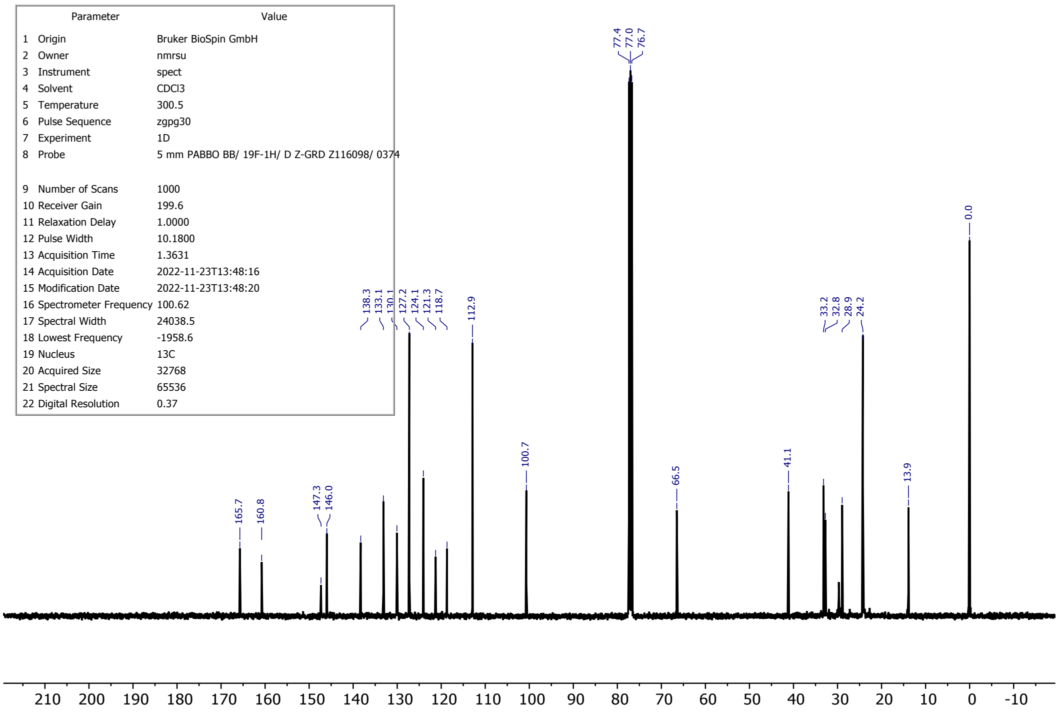

Figure S66. ^{13}C NMR (101 MHz, Chloroform-*d*) spectrum of compound **11d.**

9. Crystallographic data for **8z**

Single crystals of **8z** were obtained by recrystallisation from hexane. A red plate-shaped crystal with dimensions 0.37×0.24×0.14 mm^3 was mounted on a suitable support. Data were collected using an XtaLAB Synergy, Dualflex, HyPix diffractometer operating at T = 99.97(16) K.

Data were measured using ω scans of 0.5° per frame for 36.2 s using Mo K_α radiation. The diffraction pattern was indexed and the total number of runs and images was based on the strategy calculation from the program CrysAlisPro (Rigaku) The maximum resolution that was achieved was Θ = 27.100° (0.78 Å).

The diffraction pattern was indexed The diffraction pattern was indexed and the total number of runs and images was based on the strategy calculation from the program CrysAlisPro (Rigaku) and the unit cell was refined using CrysAlisPro (Rigaku, V1.171.44.91a, 2025) on 9651 reflections, 75% of the observed reflections.

Data reduction, scaling and absorption corrections were performed using CrysAlisPro (Rigaku, V1.171.44.91a, 2025). The final completeness is 99.80% out to 27.100° in Θ. A gaussian absorption correction was performed using CrysAlisPro 1.171.44.91a (Rigaku Oxford Diffraction, 2025). Numerical absorption correction based on gaussian integration over a multifaceted crystal model. Empirical absorption correction using spherical harmonics, implemented in SCALE3 ABSPACK scaling algorithm. The absorption coefficient μ of this material is 2.014 mm^{-1} at this wavelength (λ = 0.711Å) and the minimum and maximum transmissions are 0.291 and 1.000.

The structure was solved and the space group *Fdd*2 (# 43) determined by the ShelXT (Sheldrick, 2015) structure solution program using Intrinsic Phasing and refined by Least Squares using version 2019/2 of ShelXL 2019/2 (Sheldrick, 2015). All non-hydrogen atoms were refined anisotropically. Hydrogen atom positions were calculated geometrically and refined using the riding model. Hydrogen atom positions were calculated geometrically and refined using the riding model. **CCDC number 2540064** contains the supplementary crystallographic data for compound reported in this paper. These data can be obtained free of charge via https://www.ccdc.cam.ac.uk/.

Table S1: Fractional Atomic Coordinates (×10^4) and Equivalent Isotropic Displacement Parameters (Å^2×10^3) for **8z**. *Ueq* is defined as 1/3 of the trace of the orthogonalised *Uij*.

Atom	x	y	z	U_{eq}
Br1	2847.7(2)	8414.9(2)	5098.7(5)	40.48(16)
N2	3860.4(11)	6414.5(13)	4097(3)	26.1(7)
C6	3708.2(11)	7388.9(15)	3601(4)	19.5(7)
C4	3173.8(11)	7796.7(17)	4786(4)	26.4(8)
C1	3642.3(11)	6878.9(15)	4278(4)	21.9(8)
C16	5107.5(11)	6868.5(18)	61(5)	32.3(8)
C5	3470.8(11)	7847.5(15)	3864(4)	22.4(7)
N1	4148.2(19)	7923(3)	2202(7)	19.4(13)
C8	4253.3(12)	6940.1(15)	2547(5)	30.5(8)
C15	5212.8(12)	7415.2(18)	247(5)	32.2(9)
C3	3102.0(13)	7301.9(19)	5476(4)	33.0(9)
C19	5752.3(14)	7099(3)	1555(5)	45.4(13)
C7	4030.2(12)	7415.5(16)	2679(5)	31.1(8)
C2	3333.2(12)	6854.4(17)	5234(4)	30.4(8)
C9	4157.6(12)	6455.7(16)	3250(4)	25.8(8)
C18	5643.2(15)	6552(2)	1412(6)	47.2(14)
Atom	x	y	z	U_{eq}
C25	4403.1(15)	5945.6(16)	3019(5)	32.5(9)
C17	5319.9(14)	6443.1(19)	637(6)	42.0(12)
C20	5542.5(14)	7519(2)	991(5)	38.7(11)
C26	4318.8(18)	5670(2)	1667(5)	46.1(12)
C23	6136(5)	5806(7)	1360(20)	34(3)
C22	5641(4)	5801(8)	3204(15)	30(3)
C14	4937(2)	7814(3)	-527(7)	22.9(13)
C12	4505(2)	8010(3)	1504(6)	19.2(8)
C13	4525.8(18)	7801(2)	30(7)	19.2(8)
C21	5895(2)	6165(3)	2310(7)	26.1(14)
C13A	4693(4)	8104(5)	1119(13)	33(2)
C12A	4325(3)	7865(4)	589(11)	28(2)
C14A	5035(3)	7996(5)	79(15)	31(2)
N1A	4034(3)	7832(4)	1690(12)	24(2)
C21A	5804(4)	5925(4)	1577(12)	37(3)
C23A	6229(7)	5913(12)	1200(40)	43(7)
C22A	5768(8)	5811(14)	3120(20)	45(7)

Table S2: Anisotropic Displacement Parameters (×10^4) **8z**. The anisotropic displacement factor exponent takes the form: $-2\pi^2[h^2a^{*2} \times U_{11} + ... +2hka^* \times b^* \times U_{12}]$

Atom	U_{11}	U_{22}	U_{33}	U_{23}	U_{13}	U_{12}
Br1	33.4(2)	41.2(2)	46.9(3)	2.8(2)	16.2(2)	8.29(19)
N2	34.6(19)	19.2(15)	24.4(16)	5.7(12)	-8.6(13)	-4.3(14)
C6	18.9(17)	19.2(17)	20.4(16)	4.0(13)	-0.8(13)	-1.6(14)
C4	22.0(18)	28.9(19)	28(2)	2.1(14)	0.6(14)	1.4(15)
C1	24.9(19)	19.3(17)	21.5(17)	6.2(13)	-6.0(14)	-6.7(15)
C16	25.2(18)	48(2)	23.3(17)	-8(2)	-1.0(17)	-3.4(17)
C5	22.7(18)	21.2(17)	23.4(17)	5.1(14)	0.6(14)	-1.1(15)
N1	16(3)	17(3)	25(3)	3(2)	-2(2)	-2(2)
C8	29.3(19)	23.7(17)	38(2)	7.4(17)	7.7(18)	4.7(15)
C15	28.7(19)	37(2)	31(2)	11.7(18)	14.3(17)	10.5(17)
C3	30(2)	39(2)	29(2)	9.1(16)	9.2(15)	-7.3(18)

C19	24(2)	83(4)	29(2)	2(2)	-2.4(17)	-11(2)
C7	30.0(19)	22.8(17)	40(2)	11.2(18)	10.2(19)	4.9(15)
C2	36(2)	26.2(18)	30(2)	11.9(17)	1.5(17)	-8.7(16)
C9	32(2)	20.9(17)	24.4(19)	2.2(14)	-7.6(15)	1.2(16)
C18	27(2)	57(3)	58(3)	31(3)	9(2)	15(2)
C25	39(2)	21.2(19)	37(2)	2.3(16)	-9.7(17)	8.7(17)
C17	37(3)	24(2)	66(3)	-13(2)	18(2)	-1.5(19)
C20	37(2)	36(2)	43(2)	-15.2(19)	16(2)	-15(2)
C26	64(4)	29(2)	45(3)	-6.6(19)	-11(2)	12(2)
C23	32(7)	28(6)	42(6)	4(5)	2(5)	10(6)
C22	33(8)	31(5)	27(5)	2(3)	-6(4)	10(6)
C14	24(3)	28(3)	17(3)	4(3)	-2(3)	1(3)
C12	16(2)	23(2)	19(2)	2.9(17)	5.3(19)	0.8(17)
C13	16(2)	23(2)	19(2)	2.9(17)	5.3(19)	0.8(17)
C21	30(3)	24(3)	24(3)	-3(3)	-10(3)	7(3)
C13A	34(6)	29(5)	36(6)	-7(4)	1(5)	8(5)
C12A	36(6)	21(4)	27(5)	7(4)	-1(5)	1(4)
C14A	21(5)	39(6)	33(6)	4(6)	8(5)	1(4)
N1A	15(5)	24(5)	33(6)	8(4)	9(4)	2(4)
C21A	44(8)	31(6)	35(6)	-2(5)	-6(5)	17(5)
C23A	36(12)	42(13)	50(12)	3(9)	0(8)	7(9)
C22A	47(17)	44(11)	44(10)	-7(7)	-9(9)	21(12)

Table S3: Bond Lengths in Å for **8z**.

Atom	Atom	Length/Å
Br1	C4	1.894(4)
N2	C1	1.362(5)
N2	C9	1.327(6)
C4	C5	1.376(5)
C4	C3	1.395(6)
C1	C2	1.425(6)
C16	C15	1.380(6)
C16	C17	1.383(7)
N1	C7	1.372(7)
N1	C12	1.427(10)
C8	C7	1.388(5)
C8	C9	1.397(5)
C15	C20	1.378(7)
C15	C14	1.554(7)
C15	C14A	1.538(10)
C3	C2	1.364(7)
C19	C18	1.378(8)
C19	C20	1.366(8)

Atom	Atom	Length/Å
C6	C1	1.417(5)
C6	C5	1.401(5)
C6	C7	1.437(5)
C7	N1A	1.398(10)
C9	C25	1.512(6)
C18	C17	1.379(8)
C18	C21	1.551(7)
C18	C21A	1.620(11)
C25	C26	1.515(7)
C23	C21	1.523(15)
C22	C21	1.521(14)
C14	C13	1.523(9)
C12	C13	1.535(9)
C13A	C12A	1.489(15)
C13A	C14A	1.587(16)
C12A	N1A	1.480(15)
C21A	C23A	1.52(2)
C21A	C22A	1.55(2)

Table S4: Bond Angles in ° for **8z**.

Atom	Atom	Atom	Angle/°
C9	N2	C1	116.8(3)
C1	C6	C7	117.4(3)
C5	C6	C1	120.2(3)
C5	C6	C7	122.4(3)
C5	C4	Br1	118.8(3)
C5	C4	C3	122.0(4)
C3	C4	Br1	119.2(3)
N2	C1	C6	124.2(3)
N2	C1	C2	117.9(3)
C6	C1	C2	117.8(4)
C15	C16	C17	120.9(4)
C4	C5	C6	119.3(3)
C7	N1	C12	123.5(6)
C7	C8	C9	120.8(4)
C16	C15	C14	111.3(5)
C16	C15	C14A	138.6(6)
C20	C15	C16	117.6(4)
C20	C15	C14	131.0(5)
C20	C15	C14A	102.8(7)
C2	C3	C4	119.1(4)
C20	C19	C18	121.6(5)
N1	C7	C6	119.1(4)
N1	C7	C8	122.6(4)
C8	C7	C6	117.0(4)
C8	C7	N1A	121.5(5)
N1A	C7	C6	118.6(5)
C3	C2	C1	121.5(3)
N2	C9	C8	123.8(4)
N2	C9	C25	118.0(4)
C8	C9	C25	118.2(4)
C19	C18	C17	117.4(4)
C19	C18	C21	111.3(5)
C19	C18	C21A	142.0(7)
C17	C18	C21	131.0(5)
C17	C18	C21A	99.0(6)
C9	C25	C26	112.4(4)
C18	C17	C16	121.1(4)
C19	C20	C15	121.3(4)
C13	C14	C15	112.7(5)
N1	C12	C13	116.5(6)
C14	C13	C12	112.1(5)
C23	C21	C18	107.4(9)
C22	C21	C18	110.6(7)
C22	C21	C23	110.1(11)
C12A	C13A	C14A	110.4(10)
N1A	C12A	C13A	110.2(10)
C15	C14A	C13A	112.2(8)
C7	N1A	C12A	123.7(8)
C23A	C21A	C18	109.0(14)
C23A	C21A	C22A	108.4(18)
C22A	C21A	C18	103.6(14)

Table S5: Hydrogen Fractional Atomic Coordinates ($\times 10^4$) and Equivalent Isotropic Displacement Parameters (Å$^2\times 10^3$) for **8z**. *Ueq* is defined as 1/3 of the trace of the orthogonalised *Uij*.

Atom	x	y	z	U_{eq}
H16	4885.94	6783.13	-469.73	39
H5	3514.49	8189.8	3410.45	27
H1	3995.76	8210.16	2332.4	23
H8	4473.76	6944.26	1971.28	37
H3	2894.7	7276.73	6106.95	40
H19	5978.84	7184.56	2058.89	54
H2	3287.02	6518.69	5712.84	37
H25A	4355.3	5676.97	3762.11	39
H25B	4679.39	6051.71	3051.69	39
H17	5241.82	6069.78	495.52	50
**Atom**	**x**	**y**	**z**	U_{eq}
H20	5625.48	7891.76	1113.35	46
H26A	4052.51	5531.23	1663.65	69
H26B	4497.64	5359.22	1531.09	69
H26C	4351.83	5939.7	930.94	69
H23A	5964.63	5594.78	759.03	51
H23B	6294.04	5549.65	1896.54	51
H23C	6305.07	6043.64	809.74	51
H22A	5513.95	6031.19	3894.51	45

H22B	5800.98	5520.09	3652.87	45
H22C	5444.95	5618.75	2642.2	45
H14A	5038.14	8196.58	-461.39	28
H14B	4931.7	7709.11	-1500.47	28
H12A	4712.68	7825.8	2029.85	23
H12B	4560.49	8412.31	1503.74	23
H13A	4426.33	7416.29	-10.24	23
H13B	4358.09	8035.13	-549.11	23
H21	6069.65	6394.11	2895.1	31
H13C	4755.5	7932.35	2006.8	40
H13D	4660.92	8507.81	1260.93	40
H12C	4225.49	8100.79	-157.7	33
H12D	4374.32	7489.97	218.26	33
H14C	4937.15	8036.86	-861.21	38
H14D	5238.95	8278.73	220.93	38
H1A	3853.78	8088.63	1723.3	29
H21A	5650.62	5654.13	1026.85	44
H23D	6256.45	5954.82	213.01	64
H23E	6342.56	5559.58	1485.4	64
H23F	6363.26	6218.93	1658.56	64
H22D	5900.06	6104.81	3627.66	67
H22E	5885.49	5452.17	3335	67
H22F	5493.77	5804.74	3375.3	67

Table S6: Atomic Occupancies for all atoms that are not fully occupied in **8z**.

Atom	Occupancy	Atom	Occupancy	Atom	Occupancy	Atom	Occupancy
N1	0.6	H14A	0.6	H13C	0.4	H21A	0.4
H1	0.6	H14B	0.6	H13D	0.4	C23A	0.4
C23	0.6	C12	0.6	C12A	0.4	H23D	0.4
H23A	0.6	H12A	0.6	H12C	0.4	H23E	0.4
H23B	0.6	H12B	0.6	H12D	0.4	H23F	0.4
H23C	0.6	C13	0.6	C14A	0.4	C22A	0.4
C22	0.6	H13A	0.6	H14C	0.4	H22D	0.4
H22A	0.6	H13B	0.6	H14D	0.4	H22E	0.4
H22B	0.6	C21	0.6	N1A	0.4	H22F	0.4
H22C	0.6	H21	0.6	H1A	0.4		
C14	0.6	C13A	0.4	C21A	0.4		

References:

(1) Grams, E. S.; Silva Ramos, A.; Neves Muniz, M.; Rambo, R. S.; Alberton Perelló, M.; Sperotto, N.; Calle González, L.; Duarte, L. S.; Galina, L.; Silva Dadda, A.; Arraché Gonçalves, G.; Valim Bizarro, C.; Basso, L. A.; Machado, P. Synthesis and Antimycobacterial Evaluation of N-(4-(Benzyloxy)Benzyl)-4-Aminoquinolines. *Molecules* **2022**, *27* (8), 2556. https://doi.org/10.3390/molecules27082556.

(2) Becherer, J. D.; Boros, E. E.; Carpenter, T. Y.; Cowan, D. J.; Deaton, D. N.; Haffner, C. D.; Jeune, M. R.; Kaldor, I. W.; Poole, J. C.; Preugschat, F.; Rheault, T. R.; Schulte, C. A.; Shearer, B. G.; Shearer, T. W.; Shewchuk, L. M.; Smalley, T. L.; Stewart, E. L.; Stuart, J. D.; Ulrich, J. C. Discovery of 4-Amino-8-Quinoline Carboxamides as Novel, Submicromolar Inhibitors of NAD-Hydrolyzing Enzyme CD38. *J Med Chem* **2015**, *58* (17), 7021–7056. https://doi.org/10.1021/acs.jmedchem.5b00992.

(3) da Silva, F. F.; Paz, J. D.; Rambo, R. S.; Gonçalves, G. A.; Muniz, M. N.; de Matos Czeczot, A.; Perelló, M. A.; Berger, A.; González, L. C.; Duarte, L. S.; da Silva, A. B.; Ferreira, C. A. S.; de Oliveira, S. D.; Moura, S.; Bizarro, C. V.; Basso, L. A.; Machado, P. Unveiling the Antimycobacterial Potential of Novel 4-Alkoxyquinolines: Insights into Selectivity, Mechanism of Action, and In Vivo Exposure. *J. Med. Chem.* **2024**, *67* (24), 21781–21794. https://doi.org/10.1021/acs.jmedchem.4c01302.

(4) Czeczot, A. de M.; Muniz ,Mauro Neves; Perelló ,Marcia Alberton; Silva ,Éverton Edésio Dinis; Timmers ,Luís Fernando Saraiva Macedo; Berger ,Andresa; Gonzalez ,Laura Calle; Arraché Gonçalves ,Guilherme; Moura ,Sidnei; Machado ,Pablo; Bizarro ,Cristiano Valim; and Basso, L. A. Crystal Structure of Dihydroneopterin Aldolase from Mycobacterium Tuberculosis Associated with 8-Mercaptoguanine, and Development of Novel S8-Functionalized Analogues as Inhibitors: Synthesis, Enzyme Inhibition, in Vitro Toxicity and Antitubercular Activity. *Journal of Enzyme Inhibition and Medicinal Chemistry* **2024**, *39* (1), 2388207. https://doi.org/10.1080/14756366.2024.2388207.

(5) Perdigão, J.; Silva, H.; Machado, D.; Macedo, R.; Maltez, F.; Silva, C.; Jordao, L.; Couto, I.; Mallard, K.; Coll, F.; Hill-Cawthorne, G. A.; McNerney, R.; Pain, A.; Clark, T. G.; Viveiros, M.; Portugal, I. Unraveling Mycobacterium Tuberculosis Genomic Diversity and Evolution in Lisbon, Portugal, a Highly Drug Resistant Setting. *BMC Genomics* **2014**, *15* (1), 991. https://doi.org/10.1186/1471-2164-15-991.

(6) Carroll, P.; Muwanguzi-Karugaba, J.; Parish, T. Codon-Optimized DsRed Fluorescent Protein for Use in Mycobacterium Tuberculosis. *BMC Res. Notes* **2018**, *11* (1), 685. https://doi.org/10.1186/s13104-018-3798-3.

(7) *MIC Determination*. https://www.eucast.org/bacteria/methodology-and-instructions/mic-determination/ (accessed 2026-03-19).

(8) van Meerloo, J.; Kaspers, G. J. L.; Cloos, J. Cell Sensitivity Assays: The MTT Assay. In *Cancer Cell Culture: Methods and Protocols*; Cree, I. A., Ed.; Humana Press: Totowa, NJ, **2011**; pp 237–245. https://doi.org/10.1007/978-1-61779-080-5_20.

(9) Repetto, G.; del Peso, A.; Zurita, J. L. Neutral Red Uptake Assay for the Estimation of Cell Viability/Cytotoxicity. *Nat Protoc* **2008**, *3* (7), 1125–1131. https://doi.org/10.1038/nprot.2008.75.

(10) Moure, A. L.; Narula, G.; Sorrentino, F.; Bojang, A.; Tsui, C. K. M.; Sao Emani, C.; Porras-De Francisco, E.; Díaz, B.; Rebollo-López, M. J.; Torres-Gómez, P. A.; López-Román, E. M.; Camino, I.; Casado Castro, P.; Guijarro López, L.; Ortega, F.; Ballell, L.; Barros-Aguirre, D.; Remuiñán Blanco, M.; Av-Gay, Y. MymA Bioactivated Thioalkylbenzoxazole Prodrug Family Active against Mycobacterium Tuberculosis. *J. Med. Chem.* **2020**, *63* (9), 4732–4748. https://doi.org/10.1021/acs.jmedchem.0c00003.

(11) Westerfield M. *The Zebrafish Book. A Guide for the Laboratory Use of Zebrafish (Danio Rerio).*; University of Oregon Press: Eugene, 2000.

(12) Altenhofen, S.; Nabinger, D. D.; Wiprich, M. T.; Pereira, T. C. B.; Bogo, M. R.; Bonan, C. D. Tebuconazole Alters Morphological, Behavioral and Neurochemical Parameters in Larvae and Adult Zebrafish (Danio Rerio). *Chemosphere* **2017**, *180*, 483–490. https://doi.org/10.1016/j.chemosphere.2017.04.029.

(13) Nabinger, D. D.; Altenhofen, S.; Bitencourt, P. E. R.; Nery, L. R.; Leite, C. E.; Vianna, M. R. M. R.; Bonan, C. D. Nickel Exposure Alters Behavioral Parameters in Larval and Adult Zebrafish. *Sci Total Environ* **2018**, *624*, 1623–1633. https://doi.org/10.1016/j.scitotenv.2017.10.057.

(14) Martinelli, L. K. B.; Rotta, M.; Villela, A. D.; Rodrigues-Junior, V. S.; Abbadi, B. L.; Trindade, R. V.; Petersen, G. O.; Danesi, G. M.; Nery, L. R.; Pauli, I.; Campos, M. M.; Bonan, C. D.; de Souza, O. N.; Basso, L. A.; Santos, D. S. Functional, Thermodynamics, Structural and Biological Studies of in Silico-Identified Inhibitors of Mycobacterium Tuberculosis Enoyl-ACP(CoA) Reductase Enzyme. *Sci Rep* **2017**, *7* (1), 46696. https://doi.org/10.1038/srep46696.
